# Estimation of the cancer risk induced by therapies targeting stem cell replication and treatment recommendations

**DOI:** 10.1038/s41598-018-29967-6

**Published:** 2018-08-06

**Authors:** Michael Meyer-Hermann

**Affiliations:** 1 Department of Systems Immunology and Braunschweig Integrated Centre of Systems Biology, Helmholtz Centre for Infection Research, Rebenring 56, 38106 Braunschweig, Germany; 2 Centre for Individualised Infection Medicine (CIIM), Hannover, Germany; 3 Institute for Biochemistry, Biotechnology and Bioinformatics, Technische Universität Braunschweig, Braunschweig, Germany

## Abstract

Rejuvenation of stem cell activity might increase life expectancy by prolonging functionality of organs. Higher stem cell replication rates also bear the risk of cancer. The extent of this risk is not known. While it is difficult to evaluate this cancer risk in experiments, it can be estimated using a mathematical model for tissue homeostasis by stem cell replication and associated cancer risk. The model recapitulates the observation that treatments targeting stem cell replication can induce a substantial delay of organ failure. The model predicts that the cancer risk is minor under particular conditions. It depends on the assumed implications for cell damage repair during treatment. The benefit of rejuvenation therapy and its impact on cancer risk depend on the biological age at the time of treatment and on the overall cell turnover rate of the organs. Different organs have to be considered separately in the planning of systemic treatments. In recent years, the transfer of blood from young to old individuals was shown to bear the potential of rejuvenation of stem cell activity. In this context, the model predicts that the treatment schedule is critical for success and that schedules successful in animal experiments are not transferable to humans. Guidelines for successful protocols are proposed. The model presented here may be used as a guidance for the development of stem cell rejuvenation treatment protocols and the identification of critical parameters for cancer risk.

## Introduction

Biological organisms like humans accumulate DNA damage in replicating cells inducing many processes summarised under the general term of ageing. Without DNA damage, stem cell activity could be maintained on a level that guarantees better homeostasis of the organism. However, stem cell function declines over time and is associated with ageing^1^. Therapies targeting stem cell replication have the potential to delay ageing. For example, there is increasing evidence that the decline of stem cell function can be reverted by transfer of blood from younger individuals in liver^2^ and brain^3^. This was shown with heterochronic parabiosis mouse models^4,5^, in which young and old mice share their circulatory system. However, a long-term study with repeated injections of plasma from young to old mice did not show a significant effect^6^.

It is becoming increasingly clear that not the cellular components of young blood but soluble factors mediate the positive effect on stem cells in various organs including brain, liver, and muscles^7^. The determination of those systemic factors responsible for reactivation of stem cells is ongoing^8^, and it has been proposed that it is not the systemic factors in young blood themselves, but the dilution of inhibitory factors in old blood that would be responsible for the effect^9^.

Irrespective of the actual mechanism that induce s improved tissue homeostasis and the method used to rejuvenate stem cell activity, reactivation of damaged and silenced stem cells might well induce a higher risk of developing age-related diseases like cancer^10^. While it is impossible to quantify the degree to which stem cell reactivation would increase the risk of cancer in experimental settings, it is possible based on theoretical methods and this is the main objective of the present research. With a minimum set of assumptions, the presented analysis predicts the price of a stem cell rejuvenation therapy in terms of the impact of the therapy on cancer risk.

## Results

### A simple and generic model of organ homeostasis and cancer development

In order to ensure predictive power of the mathematical model it has to be constructed on the basis of rather generic assumptions. The model complexity is chosen such that the driving question, whether cancer risk is under control during rejuvenation therapy or not, can be addressed, avoiding speculations on molecular mechanisms. The model (see Methods: *Model equations and analytical solution*) distinguishes stem (*S*) and tissue cells (*T*). It further distinguishes two reasons for death: organ failure and cancer. More specifically, the model is based on the following set of assumptions:

- Tissue cells (*T*) have a natural life time (death rate *δ*) and are replenished by stem cell (*S*) division (proliferation rate *p*).
- The proliferation rate *p* is either constant or age-dependent according to Eq. (15).
- During treatment, the division rate is changed and reverted back to its normal value at the end of treatment sessions.
- The total population of stem cells (*σ*_0_) remains constant, ignoring both, stem cell loss and homeostatic symmetric division.
- The ratio of stem to tissue cells is 1:1000.
- The life time (Θ) is 80 years in humans and 2 years in mice.
- Death is induced by organ failure, which happens when the total number of tissue cells reaches a fraction (*f*) of 50%.
- Both, tissue and stem cells accumulate damage with rate *γ*. The kind of damage is not distinguished and only the number of accumulated damage events is considered.
- Damaged stem cells pass their damage state to tissue progeny in the course of homeostatic division.
- The damage rate *γ* is either constant, or age-dependent (Eq. (16)), or age- and division-dependent (Eq. (17)).
- Cancer is induced at a critical number of damage events per cell (*c*).
- Cancer risk at the age of 80 is *ξ* = 30%.

An important source of cell damage is mutations upon cell division. The rate of point mutations in human adult stem cells was measured in the range of 40 mutations per year^11^, where an approximately linear relationship of the number of mutations with age was found. This supports a constant damage rate (*γ*). While the number of stem cell mutations correlates with cancer incidence^12^, this absolute frequency of mutations is still difficult to relate to cancer risk because not all of these mutations happen in oncogenic regions of the genome and induce cell damage. Furthermore, the immune system is removing pathogenic cells, which makes the absolute number of mutations associated with cancer unclear. In order to keep the model simple, a linear accumulation of damage with age was used initially. However, non-linear age- and division-dependent damage rates were also considered. The cancer-inducing number of cell damage events was fixed to *c* = 10 per cell. This choice can be considered as a worst case scenario for the cancer risk induced by rejuvenation therapy because, on the one hand, cancer is expected to be induced by more than 10 accumulated damage events, and, on the other hand, with $c > 10$ the cancer risk induced by the therapy is getting smaller in the model, as was revealed by a robustness analysis (see Methods: *Robustness of the statements*).

As the division (*p*) and the cell damage rate (*γ*) are determined by the life expectancy (Θ; see Methods: *Determination of the division rate*) and the cancer risk (*ξ*; see Methods: *Determination of the damage rate*) at this age, respectively, this model has only a single free parameter: The cell death rate *δ*, which controls the overall turnover rate of tissue cells. The turnover rate is a tissue specific property and different values are considered in the following. Parameter sets for *low, intermediate*, and *high turnover* are defined in Table 1 and used as reference parameter sets.

**Table 1:** Reference model parameter sets. The rates *p*, *p*_max_, *δ*, *γ*, and *γ*_max_ are given per year. Life expectancy Θ and half times *K*_p,*γ*_, are in years. Note that *p* (Methods: *Determination of the division rate*), pmax (Methods: *Determination of the division rate of perfect homeostasis*), and max (Methods: *Determination of the damage rate*) are calculated from side conditions, thus, are not free parameters.

turnover	*σ*_0_	*τ*0	Θ	*f*	*p*	*δ*	*γ*	*c*	*ξ*
low	10^5^	10^8^	80	0.5	3.41	0.014	0.0091	10	0.3
intermediate	10^5^	10^8^	80	0.5	27.2	0.055	0.0091	10	0.3
high	10^5^	10^8^	80	0.5	68.5	0.14	0.0091	10	0.3
mouse	10^5^	10^8^	2	0.5	105	0.5	0.36	10	0.1
	**Age-dependent** ***p***			**Age-dependent** ***γ***			**Age- and** ***p*** **-dependent** ***γ***		
	***p*** **m** ***ax***	***K*** **p**	***n*** **p**	***γ*** **m** ***ax***	***Kγ***	***nγ***	***γ*** **m** ***ax***	***Kγ***	***nγ***
low	13.7	19.7	2	0.025	50	2	0.053	50	2
intermediate	54.8	60.4	2	0.025	50	2	0.031	50	2
high	137	72.4	2	0.025	50	2	0.029	50	2
mouse	500	0.4	2	0.99	1.25	2	2.44	1.25	2

The turnover rate is controlled by the cell death rate *δ*, where *low, intermediate*, and *high* turnover is used for simulations of humans. In runs with age-dependent *p*(*t*), the value for *p* is replaced by the three values pmax, *K*_p_, and *n*_p_ (see Eq. (15)), where *P*_max_ and *K*_p_ are calculated from side conditions (see Methods: *Age-dependent stem cell division rate*). In runs with age- and/or *p*-dependent *γ*(*t*), the value for *γ* is replaced by the three values *γ*_max_, *K*_*γ*_, and n_*γ*_, where *γ*_max_ is calculated from side conditions (see Methods: *Age-dependent cell damage rate*). Deviations from these sets are explicitly mentioned in the text. The robustness of the results against parameter variation is discussed in Methods: *Robustness of the statements*.

**Figure 1:**
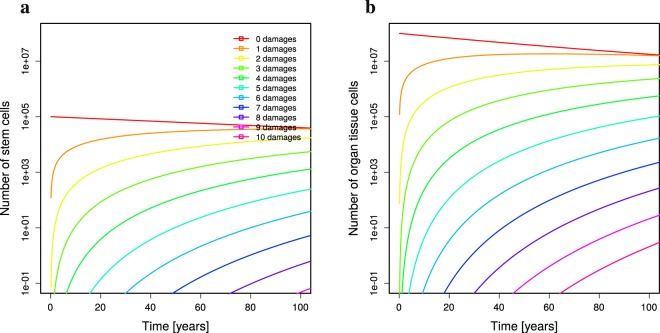
Damage dynamics in stem and tissue cells. Only undamaged stem (**a**) and tissue cells (**b**) exist in the beginning (initial conditions are *σ*_*i*_ = *τ*_*i*_ = 0 for $i > 0$ in Eq. (2)). Parameters of *low turnover* in Table 1 were used. The number of cells with different numbers of cell damage events over the life time is shown with different colours. The line with 10 damage events recollects all cells with *c* = 10 or more damage.

Starting from a young individual without any damaged cell, the time course of cell damage over age is depicted in Fig. 1. In this setting, the age of death is determined by the time, at which the total number of cells constituting the virtual organ reaches the fraction *f* = 50%. This time will be denoted as *age of organ failure* in the following. The age of cancer onset is determined by the time when the sum of all cells with *c* = 10 or more damage events exceeds the limit of 1 cell (purple lines in Fig. 1). Assuming ergodicity, it can be stated that a number of cells with 10 or more damage events below 1 reflects the cancer risk at a particular age. In the following, the *age of cancer risk ξ* will be discussed, which denotes the age at which the number of cells with *c* = 10 or more damage reaches *ξ* = 0.3.

### Organ failure is more sensitive to constant stem cell replication rates than cancer risk

**Figure 2:**
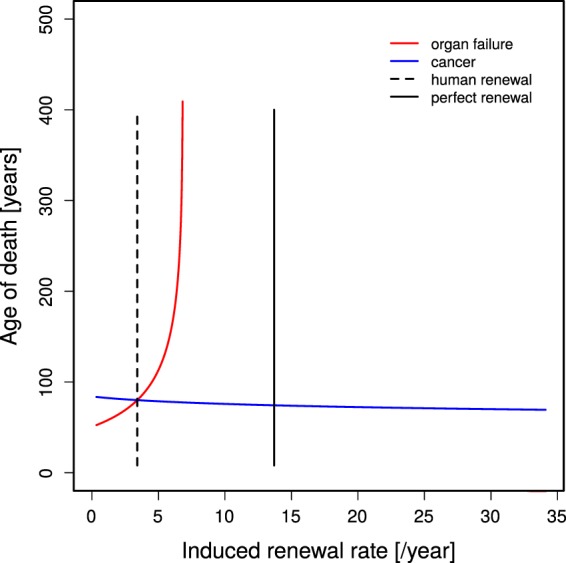
Ages of organ failure or cancer risk *ξ* in dependence on the cell renewal rate. The age of organ failure (red line, Eq. (14)) is highly sensitive to changes in the cell renewal rate (*p*) while the age of cancer risk *ξ* (blue line, Eq. (12)) remains comparably stable. Unmodified stem cell renewal *p*_normal_ (see Eq. (6)) and perfect cell renewal *p*_juvenile_ (see Eq. (11)) are marked by the vertical dashed and full lines, respectively. Between the red and the vertical black line, Eq. (14) has no solution, which means that organ failure never happens. Parameter set for low turnover in Table 1.

For life long fixed stem cell replication and damage rates, the model can be solved analytically (Eq. (2)). In order to determine the sensitivity of organ failure and cancer risk to changes in the division rate, the constant stem cell replication rate *p* is varied. Figure 2 shows how the ages of organ failure Eq. (14) and cancer risk *ξ* Eq. (12) depend on the assumed division rate. The normal division rate determined in Eq. (6) (vertical dashed line), by construction, induces organ failure and a cancer risk of *ξ* = 0.3 at the age of Θ = 80 years. The division rate for perfect tissue homeostasis determined in Eq. (11) prohibits death by organ failure. The result shows that the age of organ failure is highly sensitive to the division rate (red line in Fig. 2) while the age of cancer risk *ξ* is hardly changing (blue line in Fig. 2). For a stem cell replication rate inducing organ failure at the age of 400 (this is achieved by doubling the stem cell replication rate), the age at which the cancer risk reaches 30% is shifted from 80 to 78. Note that this result relies on life long fixed stem cell replication and damage rates.

### Organ failure is delayed with limited cancer risk

**Figure 3:**
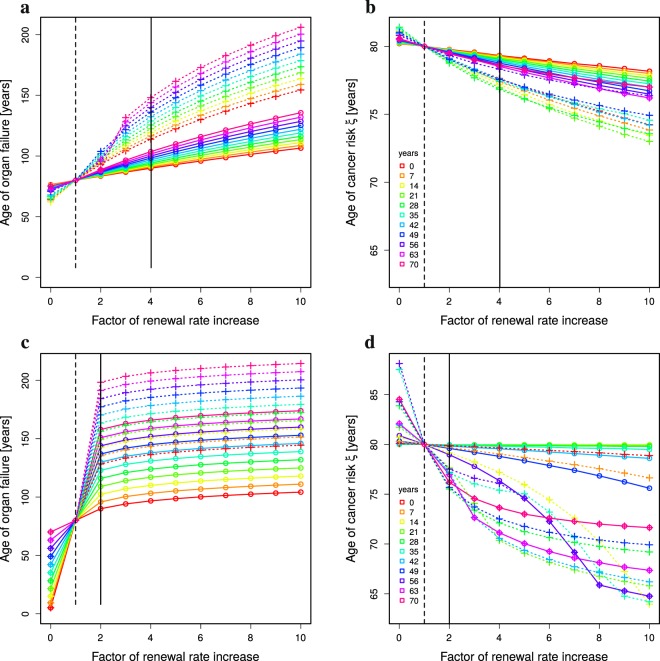
Organ failure and cancer risk with time-limited rejuvenation treatment. The analytical solution Eq. (2) together with 10 year treatments (Methods: *Treatment at particular ages*) is analysed for ages of organ failure (**a**,**c**) and cancer risk *ξ* (**b**,**d**) in dependence on the age of first treatment (colors) and with different induced renewal rate *p* (horizontal axes). A single treatment (full lines, circles) is compared to three treatments with interruptions of 10 years (dashed lines, crosses). Vertical lines denote the renewal rate of humans with a life expectancy of 80 years (dashed black line) and with perfect tissue homeostasis (full black line). Parameter set of low (**a**,**b**) and high (**c**,**d**) turnover in Table 1. Compare Supplementary Figure 1 for intermediate turnover rates.

Rejuvenation of stem cell activity is reflected in the model by changing the stem cell division rate *p* for the time of treatment. Assuming a treatment limited to a duration of 10 years starting at different ages (see Methods: *Treatment at particular ages*), a similar picture in terms of sensitivity of the ages of organ failure and cancer risk emerges (full lines in Fig. 3). The overall effect on the age of organ failures is smaller than for life long treatments (Fig. 3a,b). With an induced division rate of perfect tissue homeostasis Eq. (11), organ failure happens at ages between 85 and 100 years, and the cancer risk of *ξ* = 30% is reached at age 79 ± 1 years. When the same treatment is repeated three times every 10 years with pausing intervals of 10 years (dotted lines, Fig. 3a,b), organ failure happens at an age between 110 and 150 years and the cancer risk of 30% is reached at the age of 78 ± 1 years. While the absolute numbers have to be interpreted carefully, the difference in the sensitivity of both read-outs is a stable finding.

Organ failure is delayed the more the later the treatment is started. Cancer risk is increased correspondingly. Surprisingly, when the first of the three treatments is started at an age of 42 or later, cancer risk is getting lower when the treatment is started later (see Fig. 3a,b; blue and shorter wavelength curves). In this regime, rejuvenation treatment has a strong effect on the delay of organ failure with a comparably small increased risk of cancer. However, overall organ functionality is lower when treatment is started later. The treated individual faces a physiological state that is kept above but close to the organ failure threshold, which implies a lower life quality compared to treatments started at younger age.

### High-turnover organs are more sensitive to rejuvenation treatment but less stable

The investigation was built on organs with a low turnover rate (see Table 1, *low*). For high turnover organs (Fig. 3c,d), the sensitivity of the age of organ failure to a single rejuvenation treatment is even higher. With an induced division rate of perfect tissue homeostasis Eq. (11), organ failure happens at an age between 90 and 160 years, while the age of cancer risk *ξ* is kept between 76 and 80 years. With three treatments of 10 years, the age of organ failure can be delayed to ages of 130 to 200 years, while the cancer risk is not further increased. Importantly, for high turnover organs, a single rejuvenation treatment can delay the age of organ failure to 120 years, while inducing no major change in the cancer risk (Fig. 3c,d; full green and longer wavelength lines).

**Figure 4:**
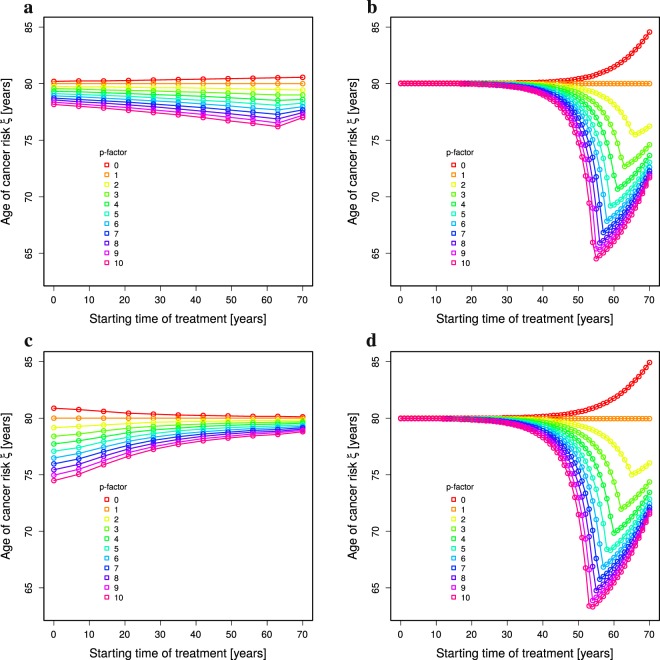
Optimal treatment age to minimise cancer risk. Cancer risk of single 10 year treatments depending on the age of treatment (horizontal axes). Constant (**a**,**b**) and age-dependent division rate (**c**,**d**) are compared for low (**a**,**c**) and high (**b**,**d**) turnover organs (see Table 1). The colors correspond to the strength of treatment as the factor of the division rate *p*. Same data as in Fig. 3 a,b (**a**), c,d (**b**), Fig. 5 c,d (**c**), and Supplementary Figure 3c,d (**d**).

Failure of high turnover organs cannot be further delayed by higher than perfect homeostasis *p*_juvenile_ stem cell replication rates. However, cancer risk enters a non-linear regime at higher replication rates (Fig. 3d). Organs with higher turnover rates react with higher or lower cancer risks than for low turnover organs depending on how and when the treatment is applied. There is hardly any increased cancer risk when the treatment is done at young age, irrespective of the target replication rate of the treatment. There is a transition to a strong impact on cancer risk at the age of 35 to 42. Cancer risk is highest for treatments started at ages around 60 and decreases again at higher ages (Fig. 4a,b).

**Figure 5:**
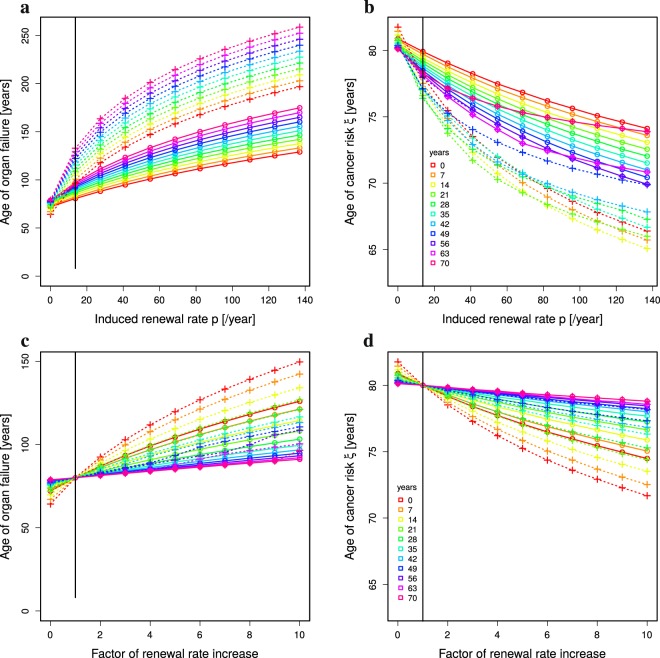
Absolute versus relative impact of rejuvenation treatment in a model with age-dependent loss of stem-cell replication. The numerical solution of Eq. (1) together with 10 year treatments (Methods: *Treatment*) is analysed for ages of organ failure (**a**,**c**) and cancer risk *ξ* (**b**,**d**) in dependence on the age of first treatment (colors) and on the strength of treatment (horizontal axes). Absolute (**a**,**b**) or relative improvements (**c**,**d**) of the renewal rate are distinguished. A single treatment (full lines, circles) is compared to three treatments with interruptions of 10 years (dashed lines, crosses). Vertical lines denote the renewal rate *p*_j*uvenile*_ inducing perfect tissue homeostasis (full black line). Parameter of low turnover in Table 1 with age-dependent stem-cell replication rate *p* in Eq. (15). For higher turnover rates compare Supplementary Figure 2 (absolute renewal rates) and Supplementary Figure 3 (relative renewal rates).

As the treatment is applied systemically, it will always also affect high turnover organs. The presented results illustrate that the timing and the dosage have to be planned quantitatively in order to avoid adverse effects.

### Limited cancer risk also in a model with age-dependent loss of stem-cell replication

A general tendency of rejuvenation treatment is that the later the treatment is started, the more organ failure is delayed and the more cancer risk is increased (Fig. 3). However, this result might rely on the assumption that stem cell replication is constant over the life time but for the periods of treatment. While this assumption is a good approximation for skin tissue, in which stem cell replication is widely conserved at all ages^13^, other organs exhibit a decrease of the stem cell replication rate with age^1^. For example, hematopoietic stem cells lose power in the course of ageing^14,15^. The degree of this reduction is again organ specific. Assuming a Hill-function for the dependence of *p* on age (see Methods: *Age-dependent stem cell division rate*), the set of differential equations Eq. (1) cannot be solved analytically anymore and is solved numerically.

Induction of higher stem cell replication rates in low turnover organs exhibits a qualitatively similar result as with constant *p* (Fig. 5a,b). An increase of the replication rate to an absolute level has a stronger effect the later in life the treatment is done. The sensitivity of the age of organ failure to treatment is still substantially higher than the sensitivity of the age of cancer risk *ξ*.

### The mechanism of rejuvenation therapy impacts on the hierarchy of best treatment age

Treatments increasing stem cell activity at a particular age can either relatively improve the remaining stem cell replication activity or induce an absolute replication level. In the particular case of young blood transfer, the mechanism of improvements in stem cell replication is not known. The aforementioned results (Fig. 5a,b), which were based on absolute renewal rates, are compared to results from a model with relative improvements of the renewal rate.

When a rejuvenation treatment induces an improved replication relative to the level of stem cell replication at the time of treatment, the situation changes in organs with low turnover rates (Fig. 5c,d). First, the overall impact on the ages of organ failure and cancer risk *ξ* is smaller. Secondly, the advantageous age of treatment is different: stem cell replication increased by a factor of ten at young age induces a substantial retardation of organ failure. However, the later the treatment is done, the weaker the effect, thus, inverting the result found with either constant stem cell replication (Fig. 3) or age-dependent stem cell replication treated to adopt absolute replication levels (Fig. 5a,b). 10 times no replication is still a weak replication. At advanced age, a treatment with a relative improvement of stem cell replication has weak effects on both, the age of organ failure (Fig. 5c) and the shift of cancer risk (Fig. 4c).

For organs with intermediate or high turnover (Supplementary Figure 3), the original sequence as found in (Figs 3 and 5a,b) is restored, i.e. the later the treatment, the more organ failure is retarded. Cancer risk is highest at ages around 60 years as it was for age-independent stem-cell replication (compare Fig. 4b,d). Whether treatment of young or old individuals is more advantageous in terms of retardation of organ failure depends on the organ turnover level with a switch-like behaviour. Hence, a turnover rate may exist, at which the treatment effect becomes widely independent of the age of treatment. This is, indeed, the case (Supplementary Figure 4) and holds true in all subsequently described model variants (Supplementary Figures 15, 27 and 39). This result suggests that, provided a treatment induces a relative increase of stem cell activity, the best age for treatment is different for different organs.

### Increasing cell damage rate with age reduces cancer risk

Similar to the division rate, the damage rate is considered age-dependent. Implicitly assuming weaker repair mechanisms at higher age, the damage rate is increased with age (see Eq. (16)). The cancer risk induced by treatments increasing stem cell replication is predicted even smaller than with age-dependent division rates alone (see Supplementary Figures 9 to 20). The impact on organ failure is not altered (Supplementary Figures 11a,c, 13a,c and 14a,c).

### Division-dependent cell damage may induce high cancer risk during treatment

Next, the cell damage rate was envisaged dependent on the division frequency of stem cells according to Eq. (17). This corresponds to the view, that cell mutations associated with cell division would be the dominant cue of cancer pathogenesis. In this setting, the amount of damage per cell might be identified with the number of oncogenic mutations.

**Figure 6:**
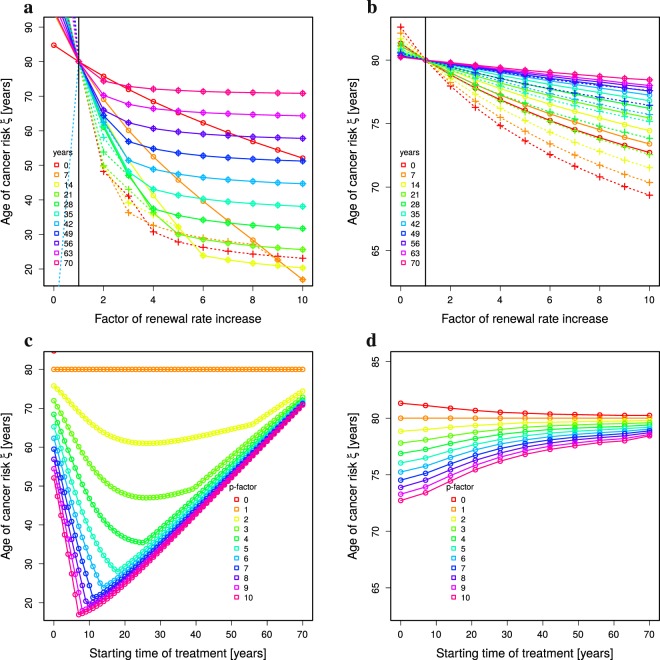
Cancer risk with age- and division-dependent cell damage. The numerical solution of Eq. (1) together with 10 year treatments (Methods: *Treatment at particular ages*) is analysed for cancer risk *ξ* in dependence on the strength of treatment by relative increase of the division rate (**a**,**b**; colors reflect the age of first treatment), and on the age of first treatment (**c**,**d**; colors reflect the strength of treatment). (**a**,**c**) The damage rate adapts to the increased division rate during treatment. (**b**,**d**) The damage rate is calculated based on the age-specific division and damage rates, also during treatment. Parameter of low turnover in Table 1 with age-dependent stem-cell replication rate *p* in Eq. (15). For absolute renewal rates compare Supplementary Figures 23 and 35, panels a,b in each. For higher turnover rates: Supplementary Figures 25 and 37 (absolute renewal rates) and Supplementary Figures 26 and 38 (relative renewal rates).

In a model with age- and division-dependent damage rates, treatments increasing stem cell replication lead to a delay of organ failure similar to constant damage (Supplementary Figures 23a,c, 25a,c and 26a,c). The impact on cancer risk highly depends on how the increased division rate impacts on the cell damage rate during treatment. If it is linearly translated into a higher damage rate, the cancer risk dramatically increases (Fig. 6a). Repeated sessions of strong treatment (factors of 4 and beyond) did not further increase the cancer risk (Fig. 6a) (dotted lines) despite a substantial delay in organ failure (Supplementary Figure 23a,c). At young ages between 10 and 20, the cancer risk is increased most and gets milder with increasing age of treatment (Fig. 6c). This cancer risk analysis also holds true for higher turnover organs (Supplementary Figures 22b, 25b,d, and 26b,d).

If the cell damage rate during treatment is derived from the division rate associated with the age of the treated *in silico* individual, the delay of organ failure remains as effective as with constant damage rates (Supplementary Figures 35a,c, 37a,c and 38a,c). The cancer risk is hardly increased (Fig. 6b) relative to a division-independent damage rate. The highest cancer risk is found for individuals at young age (Fig. 6d). In contrast, for high turnover organs, the worst age of treatment is around the age of 60 years (Supplementary Figure 34b). This basically restores the situation as found for age-dependent division and constant damage rate (Fig. 4c,d).

Taking these results together, most of the treatment induced cancer risk is related to the treatment phase, because the simulations were identical during the non-treatment phases. The impact of treatments increasing stem cell division and tissue regeneration onto cell damage and repair is critical to ascertain the harmlessness of a rejuvenation treatment. Furthermore, it can flip the most advantageous age of treatment from young to old individuals.

### Not all rejuvenation treatment reported in mice can be transfered to men

In C57BL/6 mice with a mutation associated with the development of Alzheimer’s disease, parabiosis of young and old mice for 5 weeks could ameliorate cognitive deficits developed in the old mice^2,3^. The mathematical model was challenged by testing whether an effect on the age of organ failure would be found in this setting. The life expectancy of C57BL/6 mice is in the range of 2 years (see Methods: *Comparison of mouse and human*). The model imposes a higher turnover rate (${\delta }_{{\rm{mouse}}} > 0.4$ per year) such that organ failure can happen at the age of two years (Table 1). Judging from the wound healing dynamics, the turnover rate of mouse organs might really be higher than in humans. However, the imposed higher turnover rate has to be interpreted as a phenomenological parameter recollecting all factors associated with faster ageing of mice the model does not contain. In the model, the only way of making mice age faster is a higher turnover rate.

**Figure 7:**
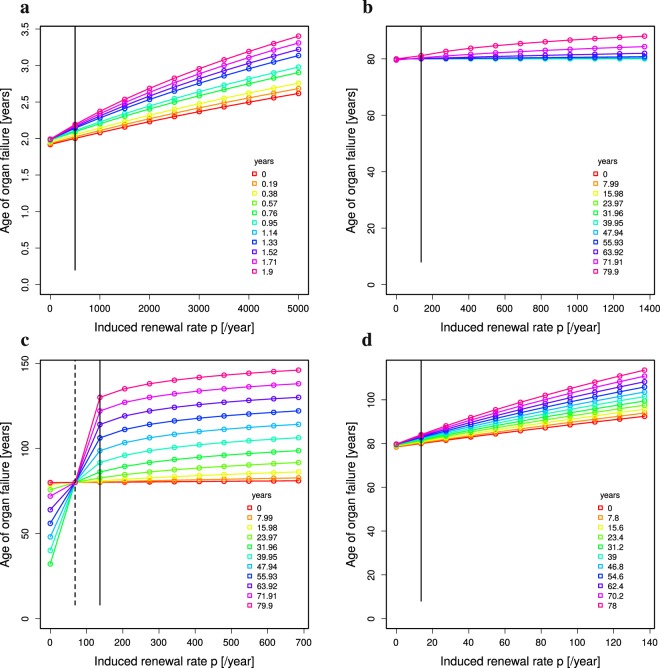
Rejuvenation treatment in mouse and human. (**a**) 5-weeks treatment of mice with life expectancy of 2 years in a model with age-dependent degradation of stem cell replication. (**b**) 5-weeks treatment of humans assuming age-dependent degradation of stem cell replication in a high turnover organ. (**c**) 5-weeks treatment of humans with age-independent stem cell replication (dashed vertical line) in a high turnover organ. The perfect homeostasis replication rate is induced during treatment (full vertical line). (**d**) 2-years treatment of humans with age-dependent degradation of stem cell replication in a low turnover organ. The parameters are taken from Table 1. The treatment was assumed to induce absolute renewal rates. For relative improvement see Supplementary Figure 5.

In the model with age-dependent loss of stem cell division, a 5 weeks rejuvenation treatment induces a substantial effect on organ failure (Fig. 7a). In contrast, a 5 weeks treatment of humans had only weak effects even if applied to high turnover organs (Fig. 7b). This remains true when applied to a human organ with even higher and unphysiological turnover rates. This implies that the positive results found in preclinical trials in mice^3^ cannot be expected in humans when the same treatment protocol is applied.

The next question was under which conditions an effect of the treatment is expected to be detectable in humans. Assuming a lifelong constant stem cell replication in high turnover organs, a 5 weeks rejuvenation treatment (inducing *p*_juvenile_ in Eq. (11)) leads to a substantial improvement (Fig. 7c). This implies that organs with high turnover and with slow ageing like the skin would benefit from short therapies. In contrast, low turnover organs with ageing stem cells like the brain will only exhibit an effect comparable to the one seen in mice when the treatment is prolonged to the scale of years (Fig. 7d). Both statement remain true, if a relative instead of an absolute improvement of stem cell replication is assumed (Supplementary Figure 5). Consistent with the results in Fig. 5, the most advantageous age of treatment in terms of cancer risk is altered in this case. Both statements also remain true, when age-dependent or age- and division-dependent replication rates are assumed (Supplementary Figures 12 and 24 for absolute, and Supplementary Figures 16 and 28 for relative improvements of stem cell replication).

## Discussion

The mathematical analysis of the impact of rejuvenation treatments improving stem cell replication and tissue regeneration predicts that organ failure is delayed at the cost of an increased risk of developing cancer. The degree of increased cancer risk depends on how the treatment modulates cell damage rates. The details of improved life time depend on the age of treatment, the turnover rate of the organ, and the mechanism of action of rejuvenation treatment.

The models with age-dependent and age-independent stem cell replication rate have many properties in common. The larger the turnover rate of the considered organ, the more efficiently organ failure is retarded by small improvements in stem cell replication. However, organ failure cannot be further delayed by stronger treatment, i.e. there is a natural limit of retardation of organ failure. In organs with higher turnover rates, the cancer risk is stable in response to moderate treatments performed at early age. This opens an opportunity to really delay organ failure without major risk to induce cancer. However, a conservative dosage of the treatment and a subtle analysis of the biological age of the individual are required. At some biological ages, the treatment can induce an unwanted increase of cancer risk.

Limited cancer risk is not found when the damage rate relied directly on the division rate. In this case, a substantially higher cancer risk is found irrespective of the turnover rate of the organs and the highest risk of cancer is found for treatments at young instead of old ages. A tolerable increase of cancer risk from 80 to 70 years of age is found for a doubling of the renewal rate, limiting possible treatment intensities. This situation corresponds to cancer pathogenesis being exclusively driven by the accumulation of mutations during stem cell division. As mutations by division are an important cause of cancer development, this is a relevant limitation. However, the increased cancer risk also relies on the assumption that the rejuvenation treatment does not alter the efficiency of DNA repair mechanisms. If the treatment not only increases tissue regeneration by stem cell replication but also rejuvenates the efficiency of repair mechanisms, cancer risk is limited as for division-independent damage rates. This implies, that for each therapy targeting stem cell activity, understanding its impact on repair mechanisms is pivotal for the estimation of the associated cancer risk.

Further, the treatment strategy has to consider the different turnover rates of the different organs. If the treatment is provided systemically, as in the case of young blood transfer, all organs are equally affected. The optimum of treatment dosage and treatment age, however, turned out to depend on the organ turnover rate. As a consequence, the treatment has to be chosen in a rather conservative way such that none of the organs bears an increased cancer risk, but as many of the organs as possible benefit from delayed organ failure.

It is important to note that the model predictions rely on averaged human data and a rather simple model of cancer pathogenesis. It only distinguishes cancer types developed from environmental damage or from division-associated mutations. The reality of cancer pathogenesis is rather variable. Different stem cell populations might be considered in future model extensions. In the current model, all results apply to the mean of these potential paths to cancer. An individual at particular risk for a specific type of cancer might be at higher or lower risk for developing cancer in response to rejuvenation treatment than estimated by the model. Therefore, the model can be used to estimate the impact of treatments on cancer risk on a population level but not as a tool for individualized therapy recommendations.

The model suggests that the best possible treatment strategy also depends on the mechanistic details of rejuvenation treatment. If the treatment induces an absolute replication rate of stem cells, the age-dependence is similar for all organ turnover rates. However, if the treatment induces an improvement of the replication rate relative to the replication potential of the stem cells at the age of treatment, treatment of older individuals can be without major effect on organ failure in low turnover organs. Furthermore, it is not clear whether treatments improve stem cell activity only during the treatment or keep the increased activity after treatment. The estimated effects would be enhanced if a treatment memory existed. Therefore, there is an urgent need to clarify the mechanistic details of the effects observed upon rejuvenation therapy, which, in particular, applies to young blood treatments^16^.

In the context of young blood rejuvenation therapy, improved tissue homeostasis was found in a mouse parabiosis model in response to 5-weeks treatments^4,5^. If the simple transfer of blood had an impact on tissue homeostasis, which is controversial^6,9^, this would open the possibility to apply young blood therapy to humans. According to the model results, the treatment schedule identified in mice with positive effect on tissue homeostasis cannot be transferred to the human case and would not induce the same degree of rejuvenation, in particular, in low turnover organs like the brain. Longer treatments in the range of years are required, which shall be considered in the interpretation of the outcome of ongoing clinical trials. A negative outcome would not necessarily imply that rejuvenation of human individuals is not possible by blood derived systemic factors. The code developed here may be used to plan clinical trials and to adapt the treatment schedule to the individual state of organs.

The strength of the presented analysis relies on the simplicity of the model based exclusively on generic assumptions and parameters determined by plausible side conditions. The analysis suggests that treatments targeting stem cell activity have a realistic chance to increase quality of life in the future. However, the treatment exhibits an age- and organ turnover rate-dependent effect and requires a decent planning. In particular, better knowledge on the induced rejuvenation mechanisms and the effect on DNA repair mechanisms are critical to avoid adverse effects.

## Methods

### Model equations and analytical solution

The model of tissue homeostasis distinguishes stem cells *S* and tissue cells *T*. Each cell type is classified by the number of accumulated damage with an index *i* for both quantities, i.e. *S*_i_ and *T*_i_. Model assumptions are described in the main text. The set of ordinary differential equations reads

1 $$\begin{array}{lll}\frac{d{S}_{0}}{dt} & = & -\,\gamma {S}_{0}\\ \frac{d{S}_{i}}{dt} & = & \gamma ({S}_{i-1}-{S}_{i})\,{\rm{for}}\,\,i > 0\\ \frac{d{T}_{0}}{dt} & = & p{S}_{0}-\delta {T}_{0}-\gamma {T}_{0}\\ \frac{d{T}_{i}}{dt} & = & p{S}_{i}-\delta {T}_{i}+\gamma ({T}_{i-1}-{T}_{i})\,{\rm{for}}\,\,i > 0\end{array}$$

with initial values ${S}_{i}({t}_{0})={\sigma }_{i}$ and ${T}_{i}({t}_{0})={\tau }_{i}$. *p* is the stem cell replication rate, *γ* is the cell damage rate, and *δ* is the death rate of tissue cells, which corresponds to the turnover rate of the considered organ.

The analytical solution of the model Eq. (1)

2 $$\begin{array}{lll}{S}_{0}(t) & = & {\sigma }_{0}{e}^{-\gamma (t-{t}_{0})}\\ {S}_{i}(t) & = & \sum _{k=0}^{i}{\sigma }_{i-k}\frac{{\gamma }^{k}}{k!}{(t-{t}_{0})}^{k}{e}^{-\gamma (t-{t}_{0})}\,\,\,{\rm{f}}{\rm{o}}{\rm{r}}\,\,i\in \,[0,\,m]\\ {T}_{0}(t) & = & ({\tau }_{0}-\frac{p{\sigma }_{0}}{\delta }){e}^{-(\delta +\gamma )(t-{t}_{0})}+\frac{p{\sigma }_{0}}{\delta }{e}^{-\gamma (t-{t}_{0})}\\ {T}_{i}(t) & = & \sum _{k=0}^{i}({\tau }_{i-k}-\frac{p{\sigma }_{i-k}}{\delta })\frac{{\gamma }^{k}}{k!}{(t-{t}_{0})}^{k}{e}^{-(\delta +\gamma )(t-{t}_{0})}\\  &  & +\sum _{k=0}^{i}\frac{p{\sigma }_{i-k}}{\delta }\frac{{\gamma }^{k}}{k!}{(t-{t}_{0})}^{k}{e}^{-\gamma (t-{t}_{0})}\,\,\,{\rm{f}}{\rm{o}}{\rm{r}}\,\,i\in \,[0,\,m]\end{array}$$

can be proven by complete induction (see Methods: *Proof of the analytical solution*). In the limit of no damage by mutations or other means (i.e. *γ* = 0), the division rate *p* can be determined by the condition of organ failure at the age of Θ = 80 years (see Methods: *Determination of the division rate*). The damage rate *γ* is determined by the side condition that the risk of cancer is *ξ* = 30% at the age of Θ = 80 years (see Methods: *Determination of the damage rate*).

*m* is the maximum number of damage events explicitly calculated, with $m\gg c$. Stem and tissue cells with *c* or more accumulated damage are recollected for presentation as

3 $$\begin{array}{c}{S}_{\ge c}(t)=\sum _{j=c}^{m}{S}_{j}(t)\\ {T}_{\ge c}(t)=\sum _{j=c}^{m}{T}_{j}(t),\end{array}$$

where *m* = 3*c* is chosen sufficiently large such that the results are independent of *m*.

### Determination of the division rate

The division rate *p* can be fixed by the condition that organ failure happens at the age of Θ. Organ failure is assumed to happen when the initial number of tissue cells drops below a fraction *f* = 0.5. It is assumed independent of the number of damage events like mutations, thus, Eq. (1) can be solved in the limit of no damage, i.e. *γ* = 0, which reduces all equations to those indexed with 0. The differential equations for stem and tissue cells read:

4 $$\begin{array}{rcl}\frac{dS}{dt} & = & 0\\ \frac{dT}{dt} & = & p{\sigma }_{0}-\delta T,\end{array}$$

where *S* = *σ*_0_ = *const*. Separation of variables, integration and incorporation of the death condition yields

5 $$\begin{array}{ccc}{\int }_{{\tau }_{0}}^{f{\tau }_{0}}\frac{dT}{{p}_{{\rm{n}}{\rm{o}}{\rm{r}}{\rm{m}}{\rm{a}}{\rm{l}}}{\sigma }_{0}-\delta T} & = & -\frac{1}{\delta }{\rm{l}}{\rm{n}}\,{({p}_{{\rm{n}}{\rm{o}}{\rm{r}}{\rm{m}}{\rm{a}}{\rm{l}}}{\sigma }_{0}-\delta T)|}_{{\tau }_{0}}^{f{\tau }_{0}}\\  & = & {\int }_{0}^{{\rm{\Theta }}}dt={\rm{\Theta }}\end{array}$$

for the unknown division rate *p*_normal_. This can be solved for the division rate *p*_normal_ resulting in

6 $${p}_{{\rm{normal}}}=\frac{\delta {\tau }_{0}}{{\sigma }_{0}}\,\frac{1-f{e}^{-\delta {\rm{\Theta }}}}{1-{e}^{-\delta {\rm{\Theta }}}}.$$

The division rate determined by Eq. (6) is used to set up the dynamics of stem cell division and tissue renewal in normal life without any treatment.

### Determination of the damage rate

The damage rate *γ* is determined by the cancer risk *ξ* at the age of life expectancy Θ, where cancer is induced at *c* damage events. Assuming no damage at birth and ignoring more than *c* damage events, this condition translates to

7 $${T}_{c}({\rm{\Theta }})=\xi .$$

This can be reformulated as

8 $$\begin{array}{lll}L & = & K{\gamma }^{c}{e}^{-\gamma {\rm{\Theta }}}\,{\rm{w}}{\rm{i}}{\rm{t}}{\rm{h}}\,\\ K & = & ({\tau }_{0}-\frac{p{\sigma }_{0}}{\delta })\,{e}^{-\delta {\rm{\Theta }}}+\frac{p{\sigma }_{0}}{\delta }\\ L & = & \frac{c!\xi }{{{\rm{\Theta }}}^{c}}\end{array}$$

An implicit equation for *γ* of this type is solved by a Lambert W-function as

9 $$\gamma =-\frac{c}{{\rm{\Theta }}}\,W(-\frac{{\rm{\Theta }}}{c}{(\frac{L}{K})}^{\frac{1}{c}}).$$

This solution is just an upper bound for *γ* because all contributions of cells with more than *c* damage events were ignored. The condition Eq. (7) with all contributions from cells with *c* or more damage reads

10 $$\begin{array}{ccc}\xi  & = & \sum _{k=c}^{m}{T}_{k}({\rm{\Theta }})\\  & = & K\sum _{k=c}^{m}\frac{{{\rm{\Theta }}}^{k}}{k!}{\gamma }^{k}{e}^{-\gamma {\rm{\Theta }}}.\end{array}$$

This equation is numerically solved for *γ* where Eq. (9) is used as an initial guess and upper bound.

### Determination of the division rate of perfect homeostasis

Perfect homeostasis implies that any loss of tissue cells by the term *δT*_*i*_ in Eq. (1) is compensated by stem cell division with the term *pS*_*i*_. As this has to happen for all damage levels *i*, it is possible to just consider the sum of all cells, which leads to the condition

11 $${p}_{{\rm{j}}{\rm{u}}{\rm{v}}{\rm{e}}{\rm{n}}{\rm{i}}{\rm{l}}{\rm{e}}}=\frac{\delta {\tau }_{0}}{{\sigma }_{0}}.$$

When *p* = *p*_juvenile_ for the whole life, organ failure never happens.

### Calculation of the age of cancer risk *ξ*

Given the cancer risk *ξ* and the constant division rate *p*, it is informative to calculate the age Θ_*c*_(*p*), at which the cancer risk reaches *ξ*. This age can be determined from the condition

12 $${T}_{c}({{\rm{\Theta }}}_{c}(p))=\xi \,=\,\sum _{k=c}^{m}\frac{1}{k!}{\gamma }^{k}{{\rm{\Theta }}}_{c}{(p)}^{k}\{({\tau }_{0}-\frac{p{\sigma }_{0}}{\delta }){e}^{-(\delta +\gamma ){{\rm{\Theta }}}_{c}(p)}+\frac{p{\sigma }_{0}}{\delta }{e}^{-\gamma {{\rm{\Theta }}}_{c}(p)}\}.$$

This condition has to be solved for Θ_*c*_(*p*), which was done by numerical approximation of the zero of Eq. (12). The relationship is shown in Fig. 2 (blue line).

### Calculation of the age of organ failure

Given the division rate *p* and the initial stem and tissue cell numbers *σ*_0_ and *τ*_0_ it is informative to calculate the age Θ_*f*_ (*p*), at which the total tissue cell numbers hit the fraction *fτ*_0_ of organ failure. As the distribution of tissue cells on classes with different levels of accumulated damage does not matter, the condition for organ failure can be calculated in the limit of no damage *γ* = 0. Starting from the analytical solution Eq. (2), the condition reads

13 $${{T}_{0}({{\rm{\Theta }}}_{f}(p))|}_{\gamma =0}=({\tau }_{0}-\frac{p{\sigma }_{0}}{\delta }){e}^{-\delta {{\rm{\Theta }}}_{f}(p)}+\frac{p{\sigma }_{0}}{\delta }\,=\,f{\tau }_{0},$$

which is solved by

14 $${{\rm{\Theta }}}_{f}(p)=\frac{1}{\delta }\,{\rm{l}}{\rm{n}}(\frac{{\tau }_{0}-\frac{p{\sigma }_{0}}{\delta }}{f{\tau }_{0}-\frac{p{\sigma }_{0}}{\delta }}).$$

Note that for larger division rates, a physiological solution does not exist in the sense of the argument of the logarithm becoming negative. This relationship is shown in Fig. 2 (red line).

### Treatment at particular ages

When the *k*-th treatment with young blood is initiated at a particular age *t*_*k*_ of the individual, the division rate *p* is changed at this particular age. This implies that the cell damage distribution ${D}_{k-1}=\{({S}_{i}({t}_{k}),{T}_{i}({t}_{k}))\,\,\,for\,i\in [0,\,m]\}$ calculated by the time *t*_*k*_ was used as initial condition for the solution Eq. (2). The development for times *t* > *t*_*k*_ are then calculated from Eq. (2) with initial condition *D*_*k*−1_, with *t*_0_ = *t*_*k*_, and with the changed *p*. When *p* is changed again, the same procedure has to be repeated. This remains true irrespective of whether the analytical solution Eq. (2) or the numerical solution with age-dependent *p* is used.

### Age-dependent stem cell division rate

Depending on the considered organ, stem cell replication is lost with increasing age. An age-dependent reduction of stem cell replication *p*(*t*) was introduced by replacing each occurrence of *p* in Eq. (1) by *p*(*t*). The solution Eq. (2) is not valid anymore and this system was solved numerically. A Hill-function was used to describe natural loss of stem cell replication as

15 $$p(t)={p}_{max}(1-\frac{{t}^{{n}_{{\rm{p}}}}}{{t}^{{n}_{{\rm{p}}}}+{K}_{{\rm{p}}}^{{n}_{{\rm{p}}}}}),$$

with the replication rate at birth *p*_max_ = *p*(*t* = 0) = *p*_juvenile_ determined from the assumption of perfect homeostasis Eq. (11). The Hill-coefficient is set to *n*_p_ = 2. The age of half replication *K*_p_ was chosen such that organ failure occurs at age Θ.

### Age-dependent cell damage rate

For the analytical solution Eq. (2) a constant damage rate *γ* was assumed. Cell damage by external factors like ionising radiation or mutations induced by cell replication are less efficiently repaired with increasing age^17–19^. This corresponds to an age-dependent damage rate

16 $$\gamma (t)={\gamma }_{{\rm{\max }}}\frac{{t}^{{n}_{\gamma }}}{{t}^{{n}_{\gamma }}+{K}_{\gamma }^{{n}_{\gamma }}},$$

where a Hill-coefficient *n*_*γ*_ = 2 and an age of half-maximum damage rate of *K*_*γ*_ = 50 were used^19^. *γ*_max_ is the maximum possible damage rate numerically determined by the condition that the cancer risk *ξ* is reached at the age Θ.

### Division-dependent cell damage rate

The cell damage rate might be associated with cell division. This reflects damage by mutations and higher division rates would also induce a higher damage rate. Assuming that the damage probability is the same in each division, the dependence on division is modelled proportional to the number of divisions and enters as a factor of the age-dependent damage rate

17 $$\gamma (t)\to \gamma (t)\frac{p(t)}{\overline{p}},$$

where $\overline{p}$ is the mean division rate over the life time, which is used to set the scale. The age-dependent functions *γ*(*t*) and *p*(*t*) are taken from Eqs (15) and (16), respectively.

One might consider to change the model Eq. (1) such that only the dividing stem cells would accumulate damage and the tissue cells would be treated with *γ*_Tissue_ = 0. This had only minor effects on the results, such that the model Eq. (1) was kept.

It has to be defined how the damage rate evolves during treatment, when the division rate is changed. Three possibilities were tested: (i) *γ* is determined by Eq. (17) based on the division rate *p* changed during treatment; (ii) *γ* is determined by Eq. (17) based on the division rate associated with the current age according to Eq. (15); (iii) *γ* is derived from Eq. (16) based on the age corresponding to the division rate during treatment according to Eq. (15).

### Comparison of mouse and human

The life expectancy of mice is in the range of two years^20^. With the organ turnover rates *δ* for humans (see Table 1), organ failure at the age of two years cannot be induced in the model (see Eq. (13)). Substantially higher turnover rates with *δ* > 0.4 per year are required to get a solution of the model with positive division rates *p*. At such high turnover rates, organ failure at the age of human life expectancy ${\rm{\Theta }}=80$ years is not achieved (see Eqs (6) and (13)).

### Robustness of the statements

The sensitivity of the results to changes in the parameter values used in Table 1 and in Eq. (15) is analysed.

The number of damage events per cell associated with induction of cancer *c* was chosen arbitrarily as *c* = 10. For very low *c* the system gets unstable. For larger *c* the behaviour and all described results remain qualitatively unchanged. There is strictly no impact of *c* on the age of organ failure. The impact of treatment on the age of cancer risk *ξ* is further reduced for larger values of *c* in all tested models (Supplementary Figures 6, 17, 29 and 41). The results based on *c* = 10 can be considered as an upper limit for the increase in cancer risk, as the number of damage events inducing cancer is likely not smaller than 10 but for extreme cases.

Larger steepness of the age-dependent division rate *n*_p_ in Eq. (15) reduces while smaller *n*_p_ increases the overall effect on both, the ages of organ failure and cancer risk *ξ*, in particular, for late treatments (see Supplementary Figure 7). This dependence on *n* is similar in all settings (Supplementary Figures 18, 30 and 42). This is consistent with the observation that in the extreme case of constant *p* the effect was larger as well (see Fig. 3). A determination of the stem cell activity in human organs over life would allow for a more concrete attribution of therapy effects *in silico* to human organs.

A corresponding analysis of the steepness of damage rate dependence on age *n*_*γ*_ with values of 1.5 and 3 shows a mild impact on the quantitative results (Supplementary Figures 19, 31 and 43). The same holds true for a variation of the age of half-maximum damage rate *K*_*γ*_ = 50 years, which was shifted to 30 and 70 years (Supplementary Figures 20, 32 and 44). Qualitative results are not affected.

A change in the relative proportion of stem cells *σ*_0_ and tissue cells *τ*_0_ has strictly no influence on the results (see Supplementary Figure 8). The stem cell division rate, by construction, compensates for a different fraction of stem cells. However, the association of the virtual turnover rates with real tissues turnover rates changes.

### Proof of the analytical solution

The ordinary differential equation system Eq. (1) can be solved analytically (see Eq. (2)). The solution can be proven by complete induction, which is sketched here. For the stem cell compartment *S*_*i*_ with $i\ge 0$ the base case is explicitly calculated. The solutions for *S*_0,1_ are trivial. Let us assume that the general solution Eq. (2) is valid for *S*_*i*_. Then

18 $$\frac{d{S}_{i+1}}{dt}=\gamma ({S}_{i}-{S}_{i+1}).$$

A differential equation of the form

19 $$\frac{dX}{dt}=b(t)+a(t)X$$

is solved by

20 $$X(t)=c{e}^{A(t)}+{e}^{A(t)}\int {e}^{-A(t^{\prime} )}b(t^{\prime} )dt^{\prime} ,$$

where *A* is a primitive of *a* and *c* is an integration constant. In the case of Eq. (18), we have

21 $$\begin{array}{ccc}b(t) & = & \gamma {S}_{i}(t)\\ a(t) & = & -\gamma \\ A(t) & = & -\gamma t\end{array}$$

where *S*_*i*_ is taken from the assumed solution Eq. (2). Then

22 $$\begin{array}{rcl}{S}_{i+1}(t) & = & c{e}^{-\gamma t}+{e}^{-\gamma t}\int {e}^{\gamma t^{\prime} }\sum _{k\mathrm{=0}}^{i}{\sigma }_{i-k}\frac{{\gamma }^{k+1}}{k!}{(t^{\prime} -{t}_{0})}^{k}{e}^{-\gamma (t^{\prime} -{t}_{0})}dt^{\prime} \\  & = & c{e}^{-\gamma t}+{e}^{-\gamma (t-{t}_{0})}\sum _{k\mathrm{=0}}^{i}{\sigma }_{i-k}\frac{{\gamma }^{k+1}}{(k+\mathrm{1)!}}{(t-{t}_{0})}^{k+1}.\end{array}$$

With the initial condition

23 $${S}_{i+1}({t}_{0})={\sigma }_{i+1}\,=\,c{e}^{-\gamma {t}_{0}}$$

the integration constant is determined as

24 $$c={\sigma }_{i+1}{e}^{\gamma {t}_{0}}.$$

Insertion into Eq. (22) after some algebra yields

25 $${S}_{i+1}(t)=\sum _{k\mathrm{=0}}^{i+1}{\sigma }_{i+1-k}\frac{{\gamma }^{k}}{k!}{(t-{t}_{0})}^{k}{e}^{-\gamma (t-{t}_{0})},$$

which is what needed to be shown.

For the tissue compartment the base case is calculated for *T*_0_:

26 $$\begin{array}{ccc}\frac{d{T}_{0}}{dt} & = & p{S}_{0}-(\delta +\gamma ){T}_{0}\\  & = & {\sigma }_{0}p{e}^{-\gamma (t-{t}_{0})}-(\delta +\gamma ){T}_{0},\end{array}$$

where the explicit solution for *S*_0_ was inserted. This equation is solved with Eq. (20) and the initial condition *T*_0_(*t*_0_) = *τ*_0_ is used to yield

27 $${T}_{0}(t)=({\tau }_{0}-\frac{{\sigma }_{0}p}{\delta }){e}^{-(\delta +\gamma )(t-{t}_{0})}+\frac{{\sigma }_{0}p}{\delta }{e}^{-\gamma (t-{t}_{0})}.$$

This shows that the explicit solution of *T*_0_ is consistent with the general solution Eq. (2). Now the solution Eq. (2) is assumed valid for *T*_*i*_. Then

28 $$\frac{d{T}_{i+1}}{dt}=p{S}_{i+1}+\gamma {T}_{i}-(\delta +\gamma ){T}_{i+1},$$

where *S*_*i* + 1_ is known from the already proven solution Eq. (2) and *T*_*i*_ can be inserted from the assumed solution. Again Eq. (20) can be applied with

29 $$\begin{array}{rcl}b(t) & = & p{S}_{i+1}(t)+\gamma {T}_{i}(t)\\ a(t) & = & -(\delta +\gamma )\\ A(t) & = & -(\delta +\gamma )t\end{array}$$

Together with

30 $$\begin{array}{rcl}\int {(t^{\prime} -{t}_{0})}^{k}dt^{\prime}  & = & \frac{1}{k+1}{(t-{t}_{0})}^{k+1}\\ \int {(t^{\prime} -{t}_{0})}^{k}{e}^{\delta t^{\prime} }dt^{\prime}  & = & \sum _{j\mathrm{=0}}^{k}{(-)}^{k-j}\frac{k!}{j!{\delta }^{k+1-j}}{(t-{t}_{0})}^{j}{e}^{\delta t}\end{array}$$

and some algebra, one finds

31 $$\begin{array}{ccl}{T}_{i+1}(t) & = & c{e}^{-(\delta +\gamma )t}+\sum _{k=0}^{i+1}\frac{p{\sigma }_{i+1-k}}{\delta k!}{\gamma }^{k}{(t-{t}_{0})}^{k}{e}^{-\gamma (t-{t}_{0})}\\  &  & +\sum _{k=0}^{i}({\tau }_{i-k}-\frac{p{\sigma }_{i-k}}{\delta })\frac{{\gamma }^{k+1}}{(k+1)!}{(t-{t}_{0})}^{k+1}{e}^{-(\delta +\gamma )(t-{t}_{0})}.\end{array}$$

With the initial condition *T*_*i* + 1_(*t*_0_) = *τ*_*i* + 1_ the integration constant is determined as

32 $$c=({\tau }_{i+1}-\frac{p{\sigma }_{i+1}}{\delta }){e}^{(\delta +\gamma )(t-{t}_{0})}.$$

Insertion in Eq. (31), resorting, and renumbering of the sum indices yields:

33 $$\begin{array}{ccc}{T}_{i+1}(t) & = & \sum _{k=0}^{i+1}\frac{p{\sigma }_{i+1-k}}{\delta k!}{\gamma }^{k}{(t-{t}_{0})}^{k}{e}^{-\gamma (t-{t}_{0})}\\  &  & +\sum _{k=0}^{i+1}({\tau }_{i+1-k}-\frac{p{\sigma }_{i+1-k}}{\delta })\frac{{\gamma }^{k}}{k!}{(t-{t}_{0})}^{k}{e}^{-(\delta +\gamma )(t-{t}_{0})},\end{array}$$

which proves the solution Eq. (2) for all *i*.

## Electronic supplementary material

Supplementary Information
