# Supplementary material for: Estimation of the cancer risk induced by therapies targeting stem cell replication and treatment recommendations
Source: Sci Rep. 2018 Aug 6;8:11776. doi: 10.1038/s41598-018-29967-6 (PMC6078959; doi:10.1038/s41598-018-29967-6)
Supplement: Supplementary file 1 — Supplementary Information [file 41598_2018_29967_MOESM1_ESM.pdf]

Supplementary material to
Estimation of the cancer risk induced by therapies targeting stem cell replication and treatment recommendations

Michael Meyer-Hermann[1,2,3]

[1]Department of Systems Immunology and Braunschweig Integrated Centre of Systems Biology, Helmholtz Centre for Infection Research, Rebenring 56, 38106 Braunschweig, Germany

[2]Centre for Individualised Infection Medicine (CIIM), Hannover, Germany

[3]Institute for Biochemistry, Biotechnology and Bioinformatics, Technische Universität Braunschweig, Braunschweig, Germany

Correspondence: mmh@theoretical-biology.de

Supplementary figures

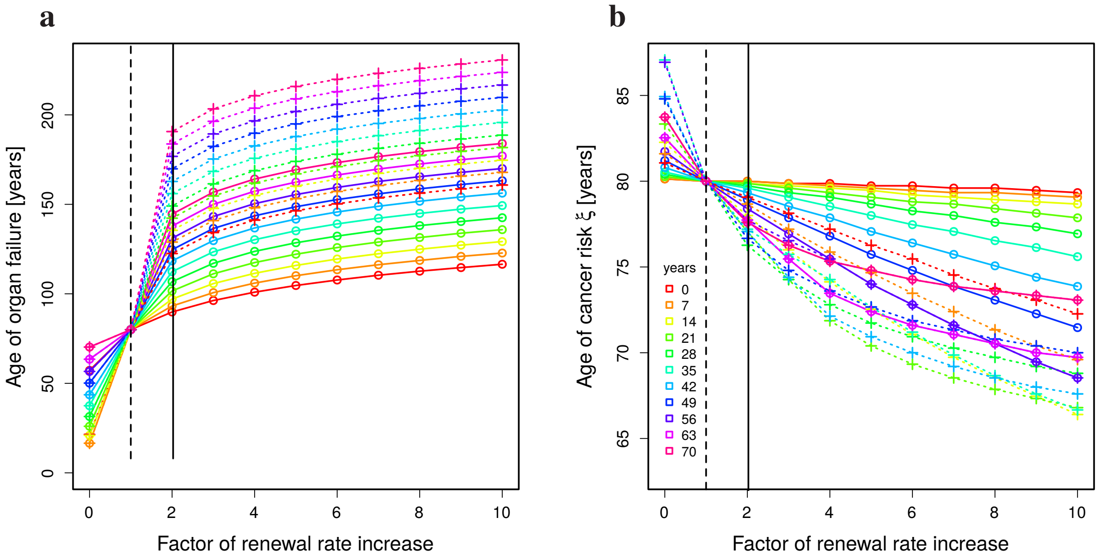

Supplementary Figure 1: Constant renewal with intermediate turnover rates.
Same analysis and representation as in Figure 3 but for organs with intermediate turnover (see Table 1).

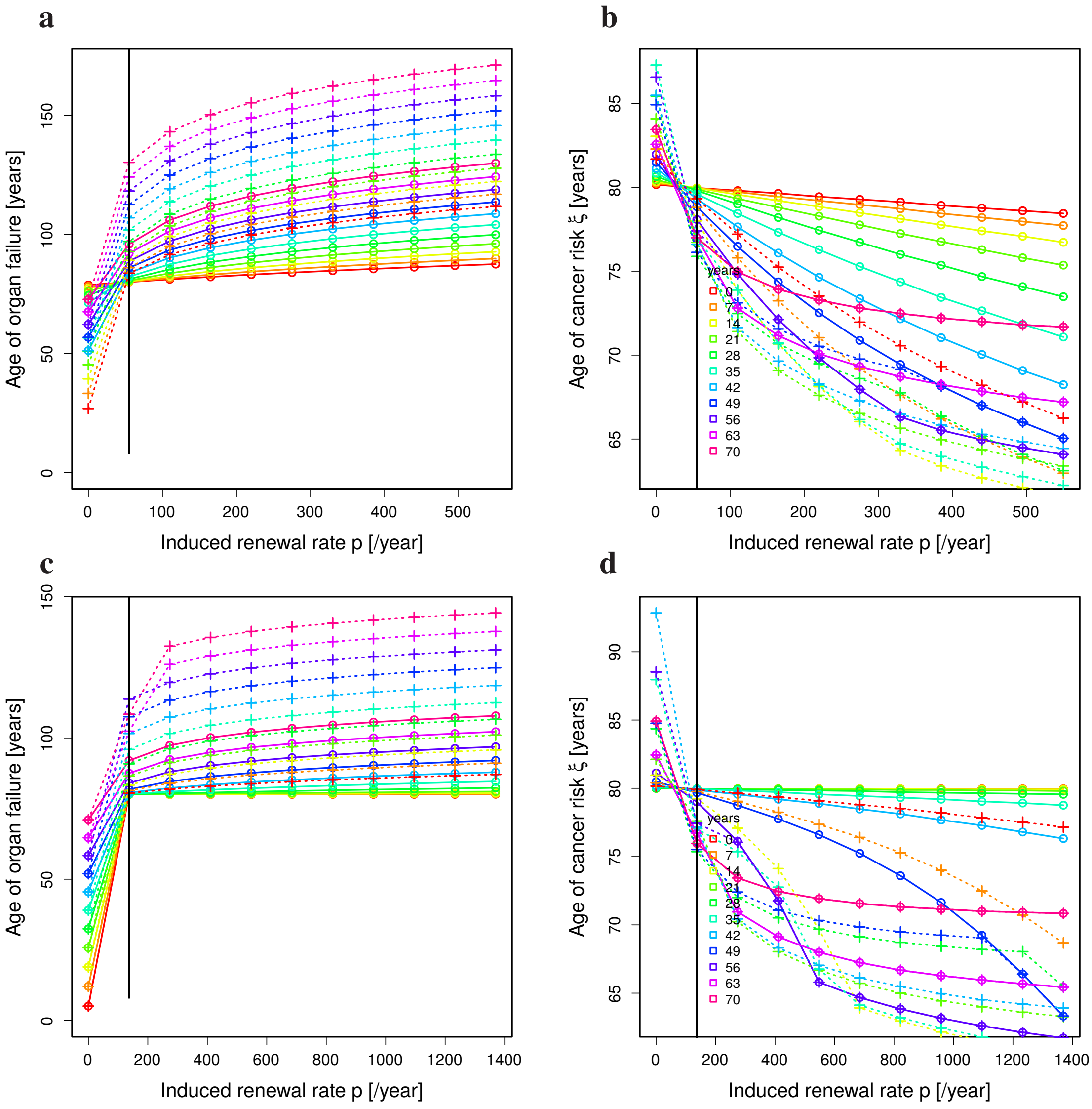

Supplementary Figure 2: Age-dependent renewal rate for higher turnover organs (absolute p). Same analysis (i.e. inducing absolute renewal rates by the treatment) and representation as in Figure 5a,b but for organs with intermediate **(a,b)** or high **(c,d)** turnover (see Table 1).

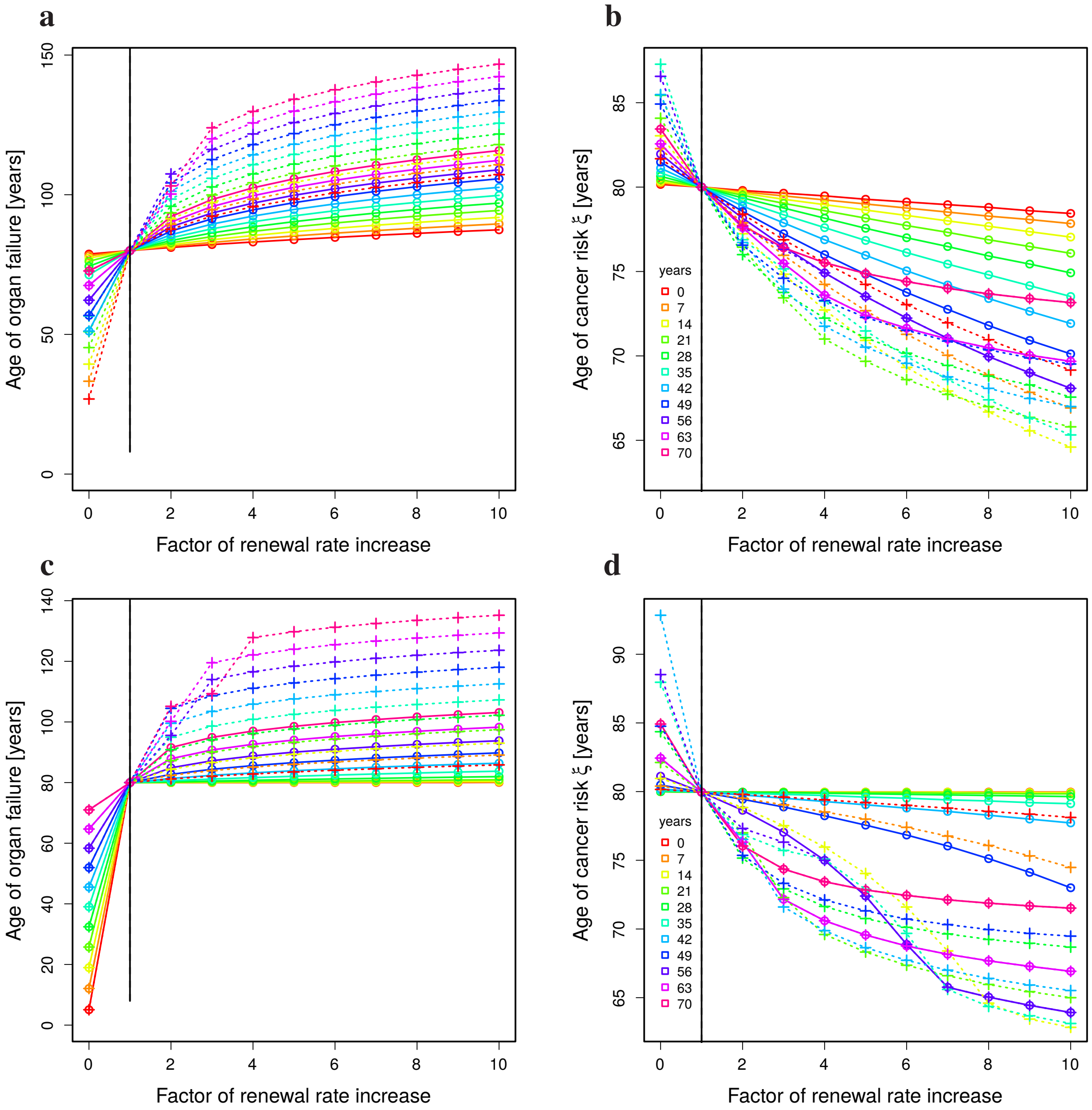

Supplementary Figure 3: Age-dependent renewal rate for higher turnover organs (relative p). Same analysis (i.e. inducing relative improvements of the renewal rate by the treatment) and representation as in Figure 5c,d but for organs with intermediate **(a,b)** or high **(c,d)** turnover (see Table 1). The hierarchy, that organ failure happens the later the older the treated individual is restored **(a,c)**. Cancer risk analysis **(b,d)** is consistent with the case of constant replication rate p (compare Figure 3).

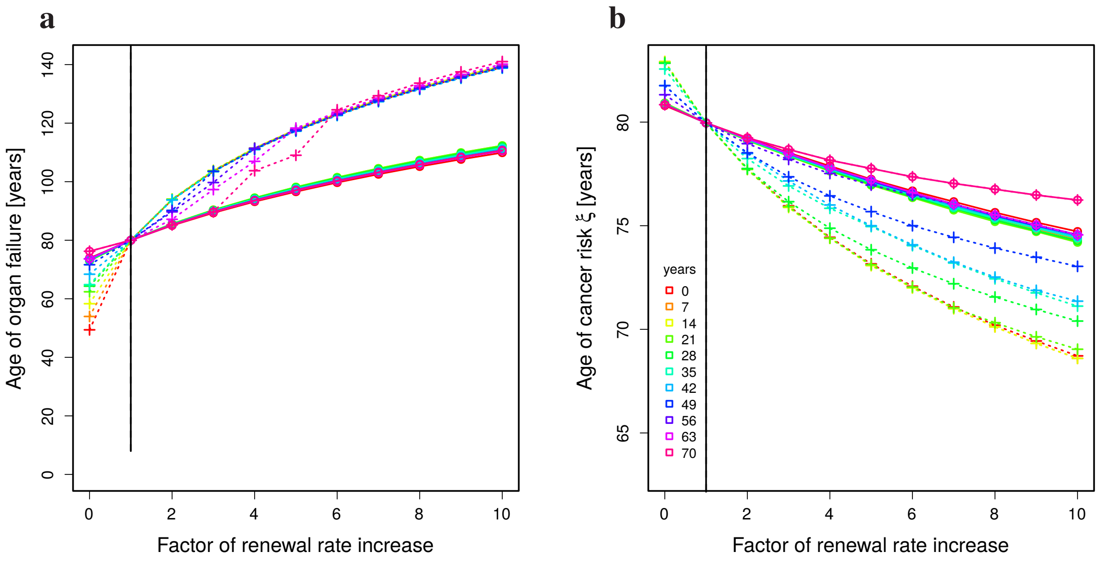

Supplementary Figure 4: Organ turnover rate of age-independent treatment success. Same analysis and representation as in Figure 5 with the critical death rate $\delta = 0.023$ per year, at which the age of first treatment becomes irrelevant for the retardation of organ failure. Relative impact of treatment on stem cell replication.

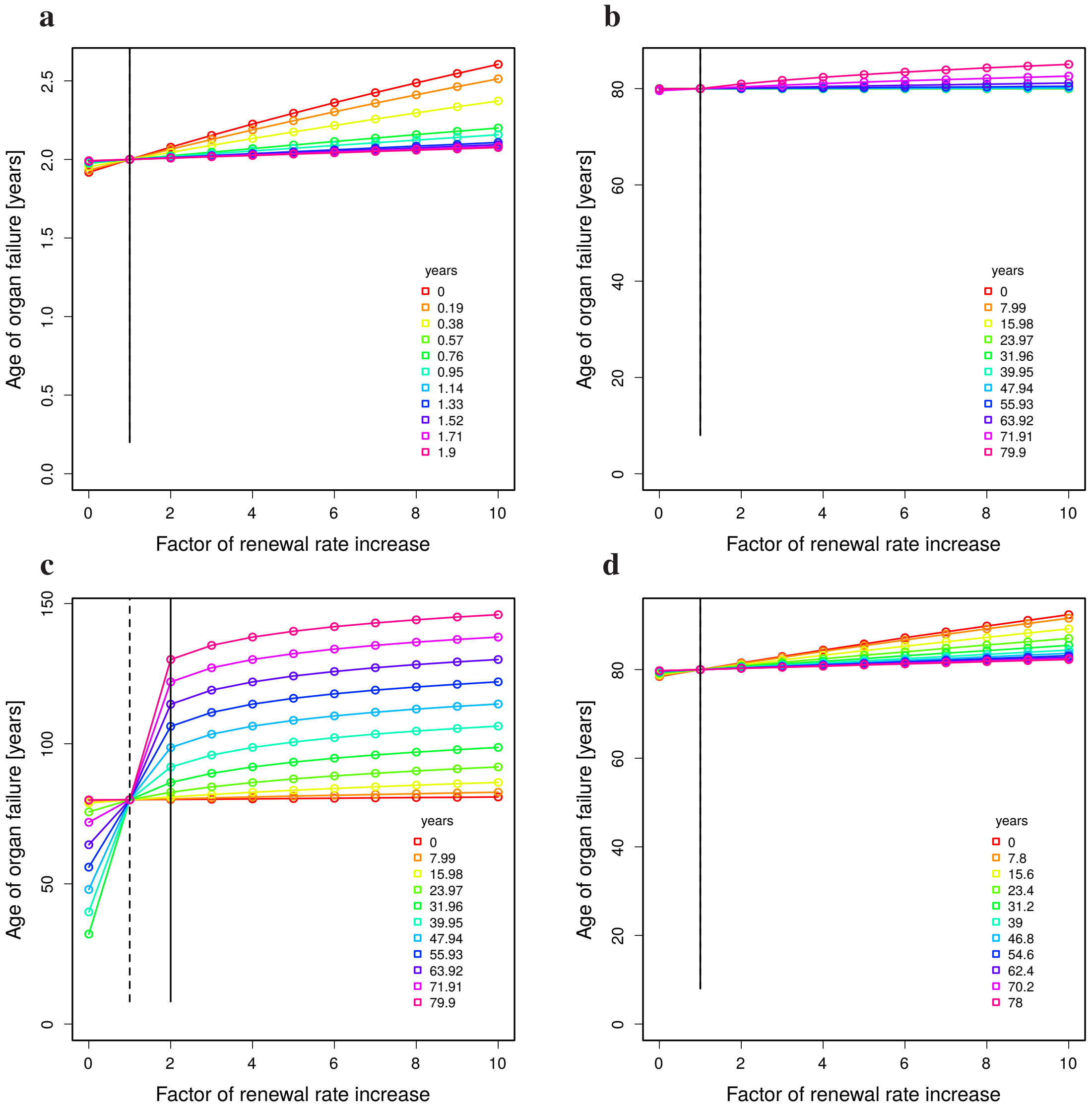

Supplementary Figure 5: Rejuvenation treatment in mouse and human.
Same experiment as in Figure 7 but for relative induced stem cell replication rates (horizontal axes). **(a)** Mouse, age-dependent renewal, treated 5 weeks. **(b)** Human, age-dependent renewal, high turnover organ, treated 5 weeks. **(c)** Human, age-independent renewal, high turnover organ, treated 5 weeks. **(d)** Human, age-dependent renewal, low turnover organ, treated 2 years.

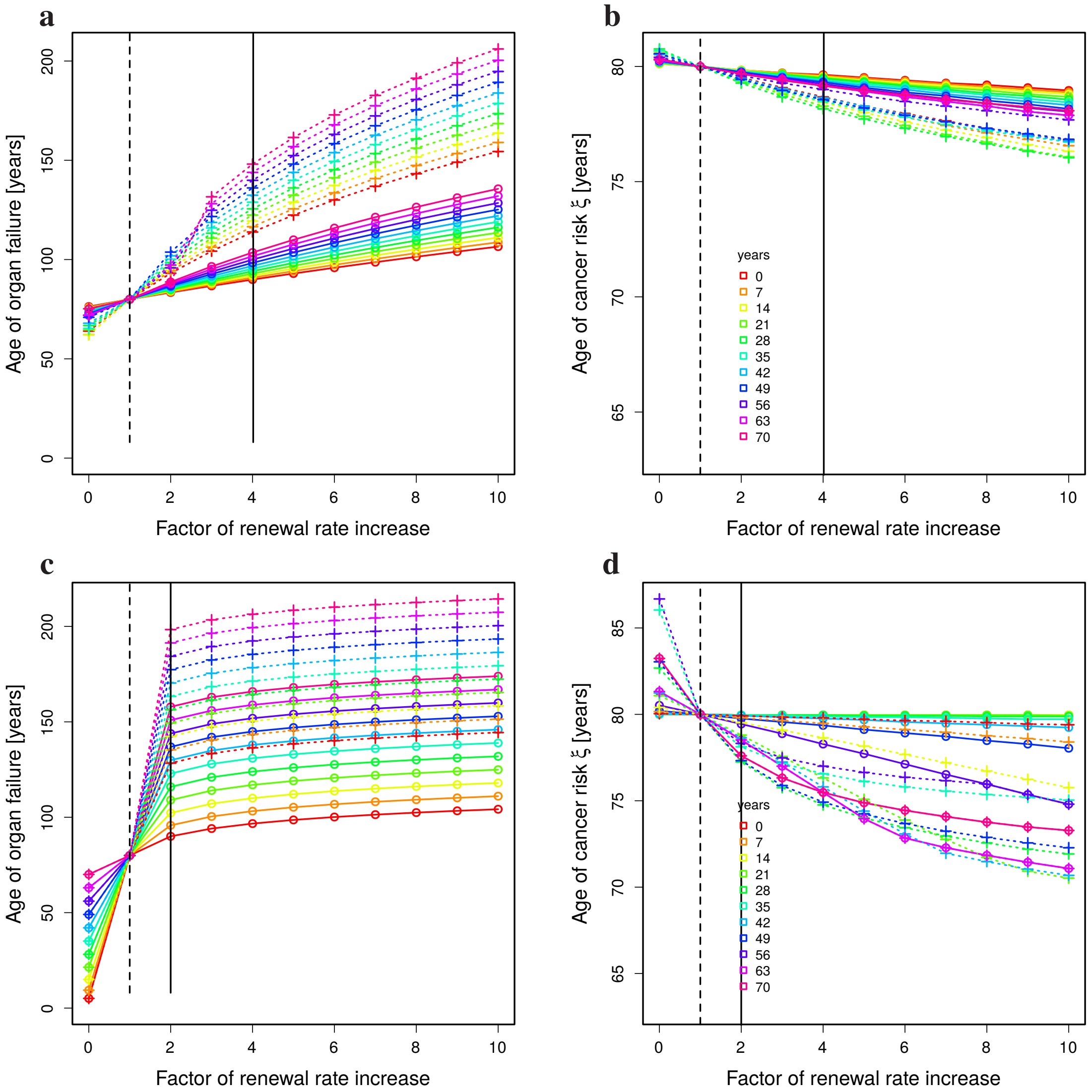

Supplementary Figure 6: Constant renewal rate with cancer induced at $c = 20$ cell damages. Same analysis and representation as in Figure 3 with low **(a,b)** and high **(c,d)** turnover rates (see Table 1).

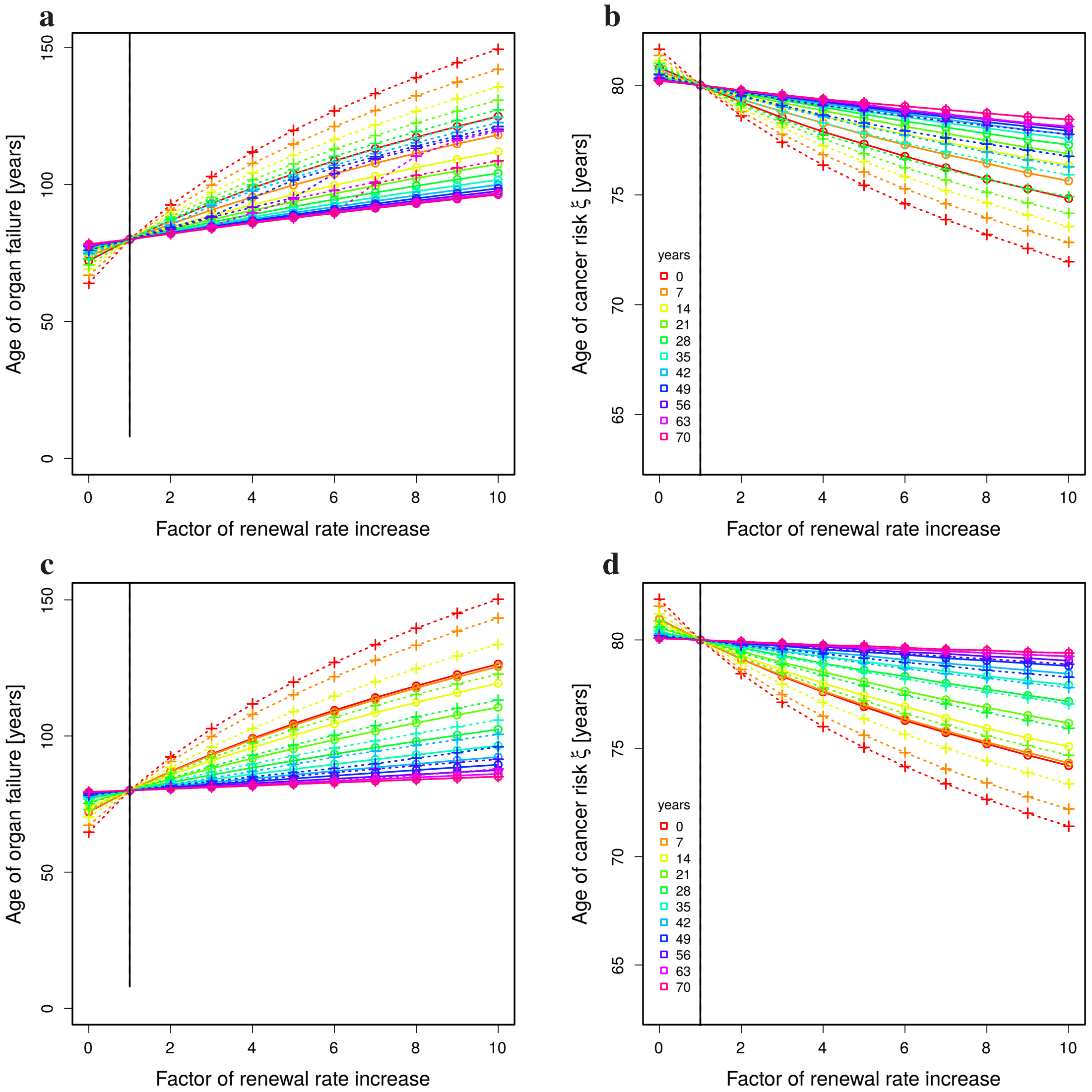

Supplementary Figure 7: Steep or flat age-dependence of the renewal rate.
Same analysis and representation as in Figure 5c,d with flat ($n_{\mathrm{p}} = 1.5$, **a,b**) and steep ($n_{\mathrm{p}} = 3$, **c,d**) age-dependence of the division rate p in Eq. (15). Parameters of low turnover rates (see Table 1).

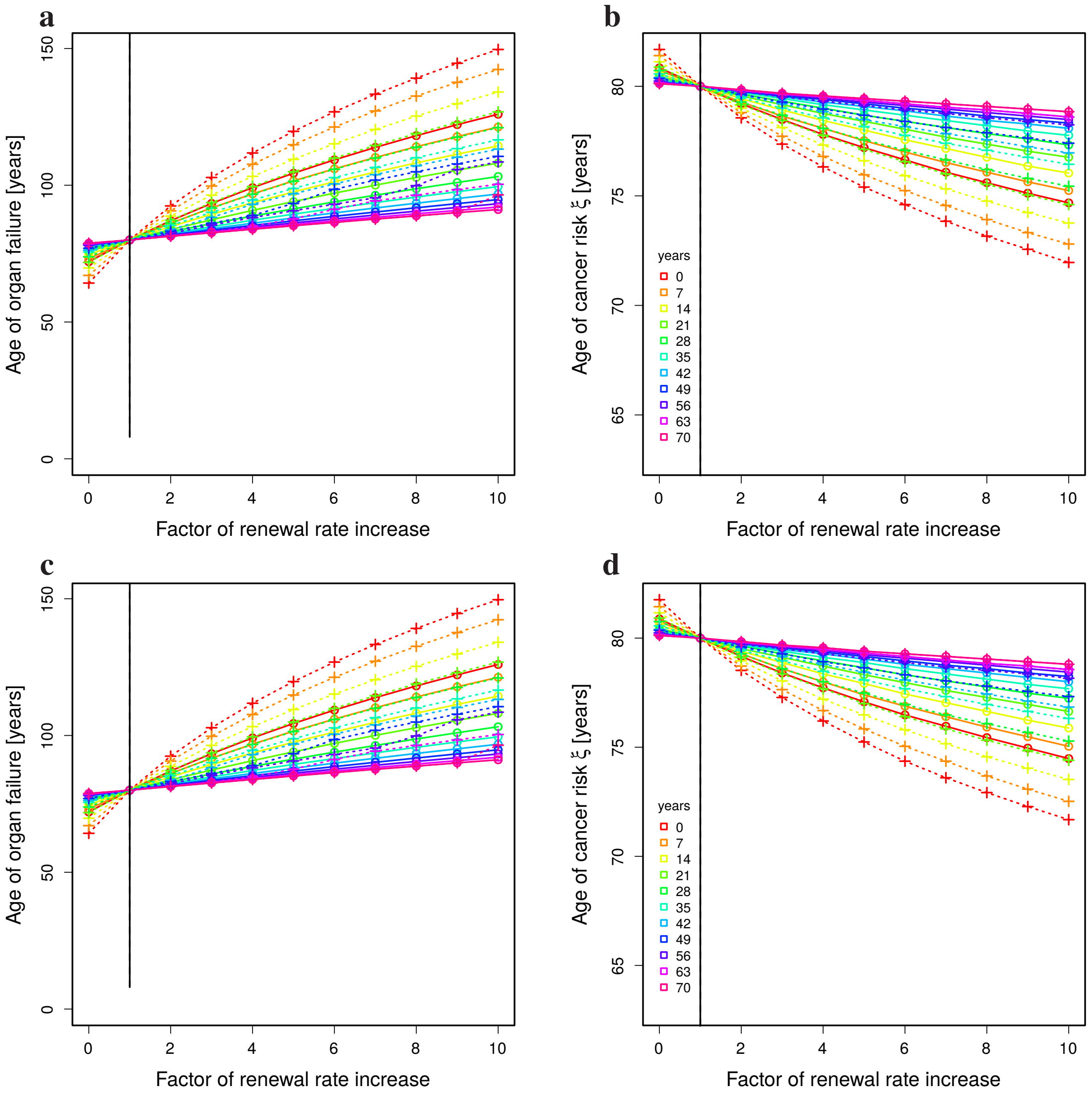

Supplementary Figure 8: Increased total cell numbers.
Same analysis and representation as in Figure 5c,d with 1000 times more cells **(a,b)** and 10 times more stem cells **(c,d)**. There is no impact by construction. Using age-dependent renewal rate and relative improvement in 10 years treatments. Parameters of low turnover rates (see Table 1).

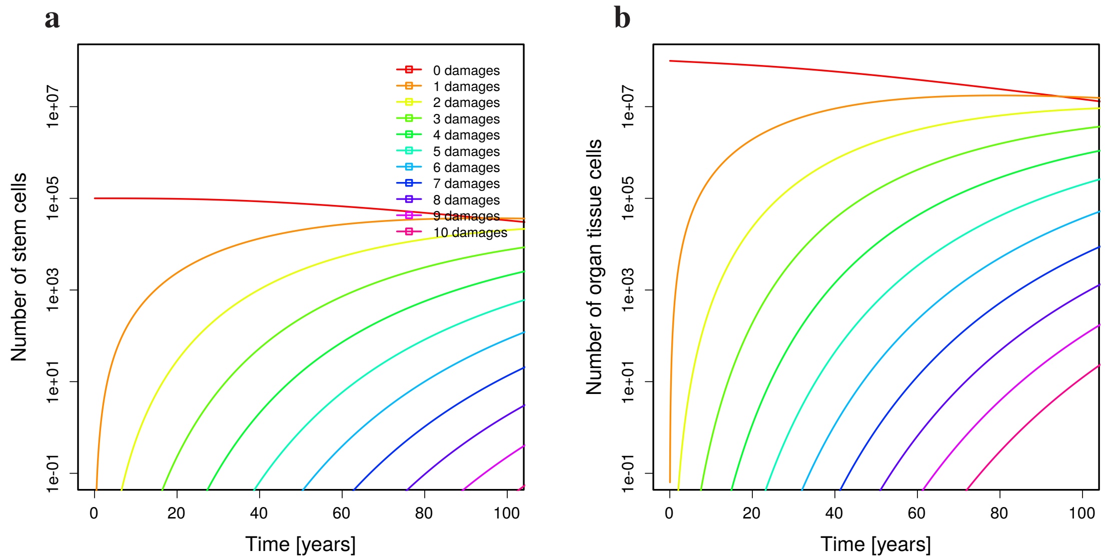

Supplementary Figure 9: Figure 1 with age-dependent damage rate Eq. (16). Low turnover parameter in Table 1.

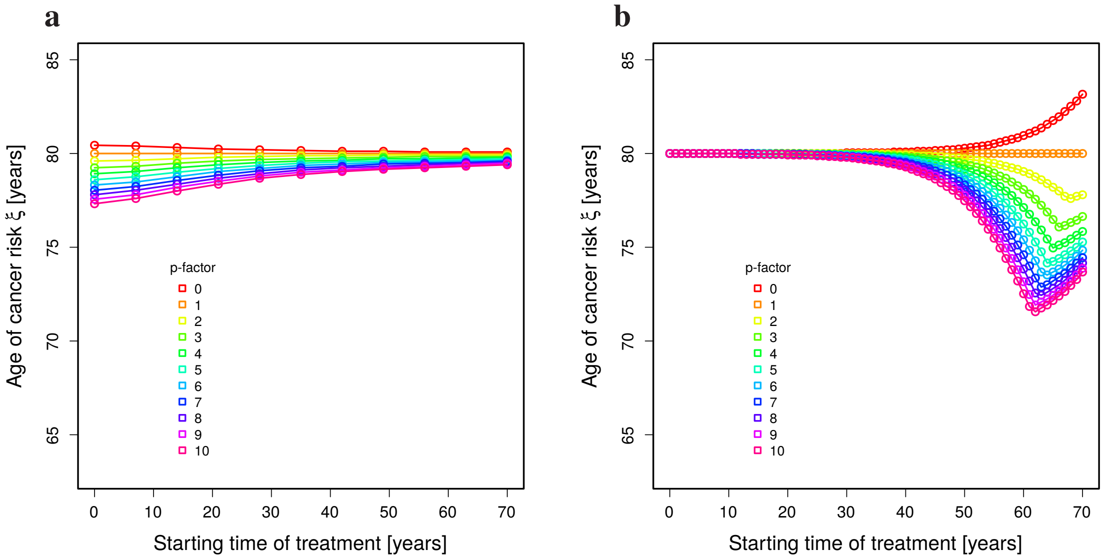

Supplementary Figure 10: Figure 4c,d with age-dependent damage rate Eq. (16). Low (a) and high (b) turnover parameter in Table 1.

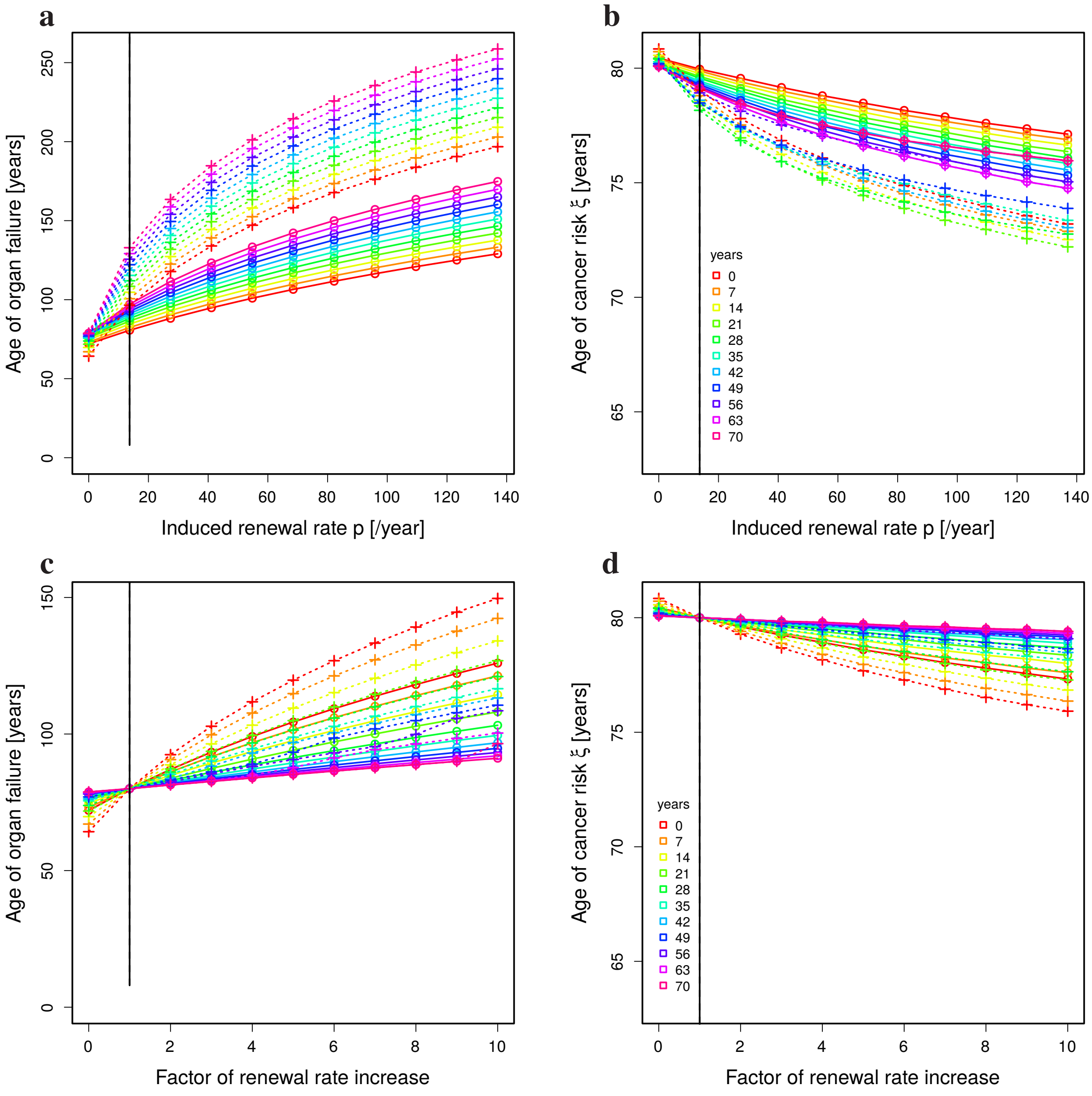

Supplementary Figure 11: Figure 5 with age-dependent damage rate Eq. (16). Low turnover parameter in Table 1. Absolute **(a,b)** or relative **(c,d)** improvement of the renewal rate during treatment.

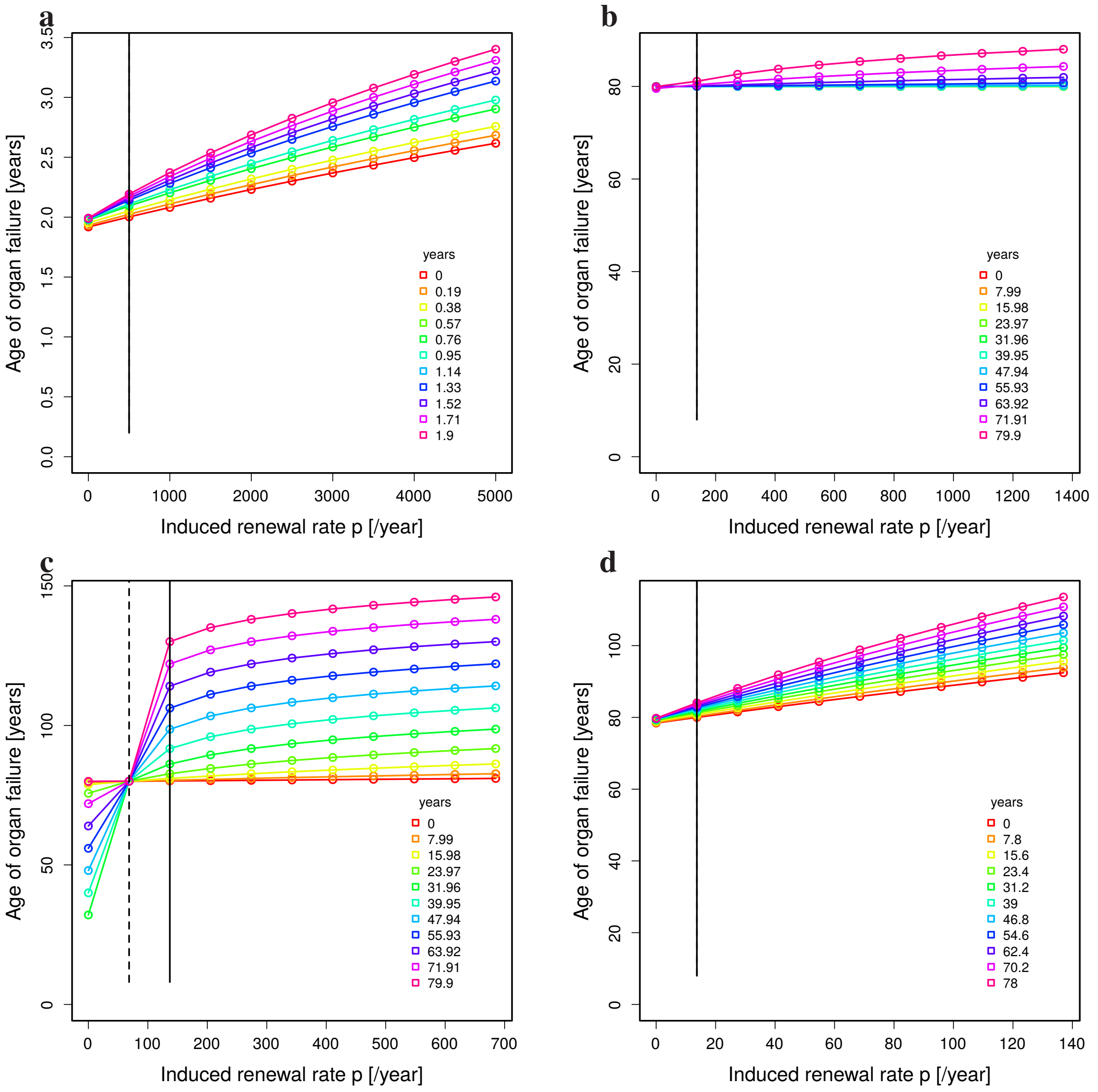

Supplementary Figure 12: Figure 7 with age-dependent damage rate Eq. (16). **(a)** Mouse, age-dependent renewal, treated 5 weeks. **(b)** Human, age-dependent renewal, high turnover organ, treated 5 weeks. **(c)** Human, age-independent renewal, high turnover organ, treated 5 weeks. **(d)** Human, age-dependent renewal, low turnover organ, treated 2 years. Absolute improvement of the renewal rate during treatment.

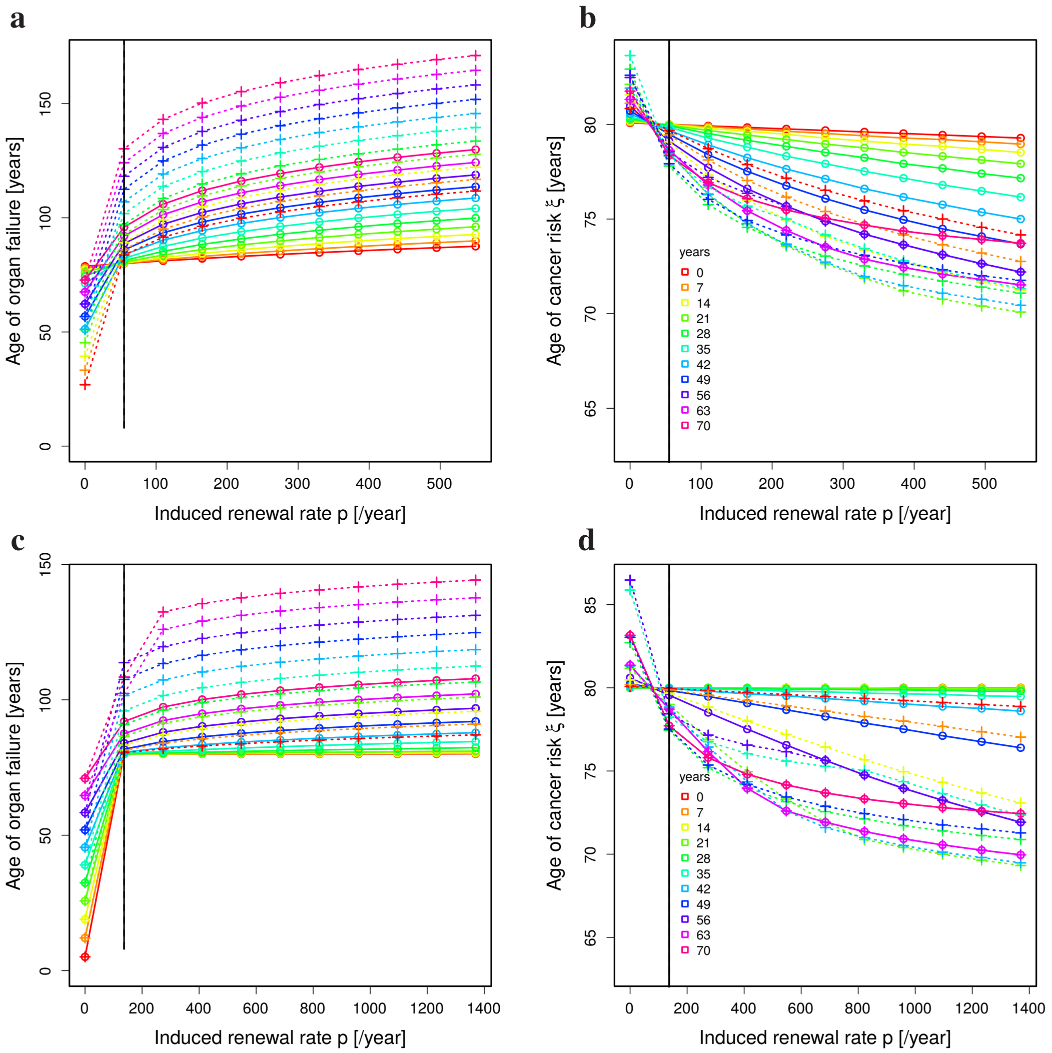

Supplementary Figure 13: Supplementary Figure 2 with age-dependent damage rate Eq. (16). Intermediate **(a,b)** or high **(c,d)** turnover parameter in Table 1. Absolute improvement of renewal rate during treatment.

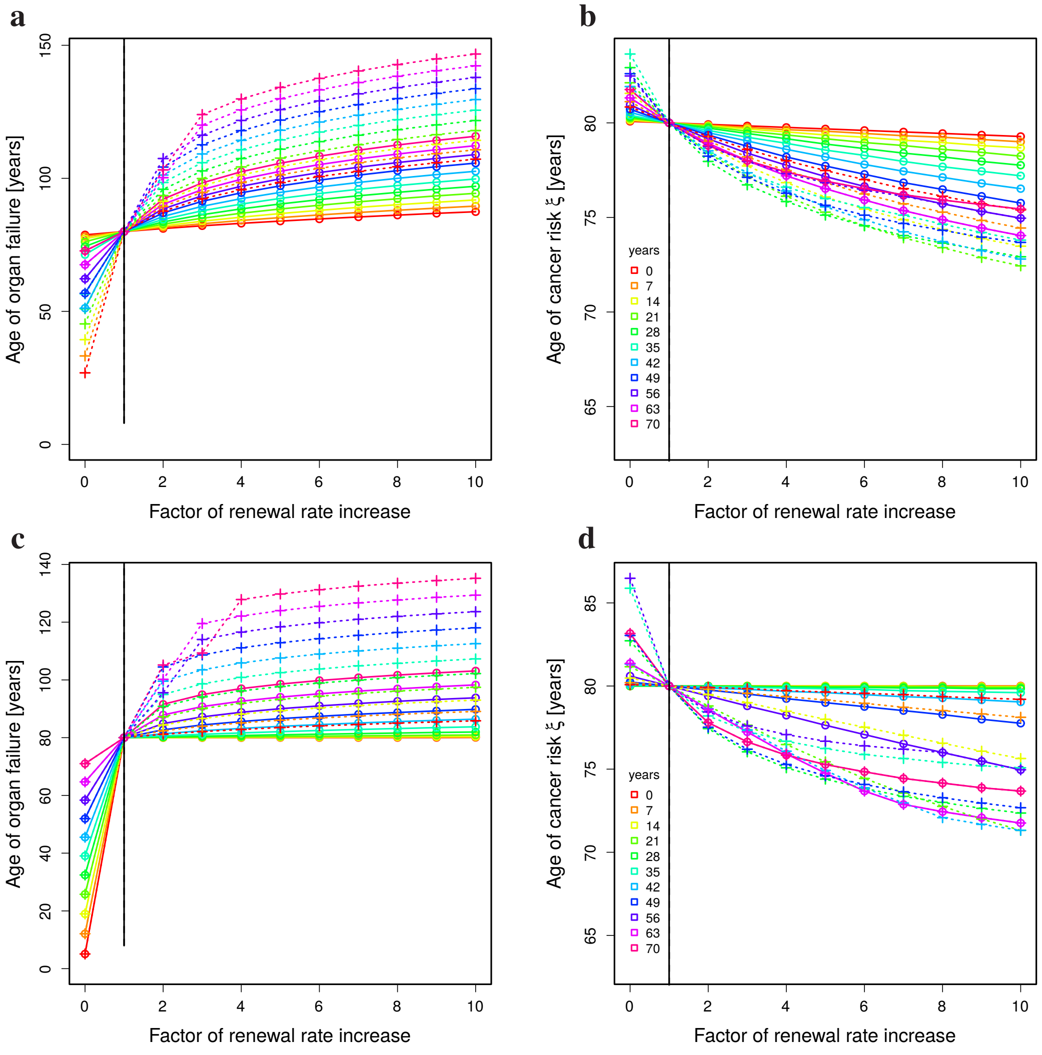

Supplementary Figure 14: Supplementary Figure 3 with age-dependent damage rate Eq. (16). Intermediate **(a,b)** or high **(c,d)** turnover parameter in Table 1. Relative improvement of renewal rate during treatment.

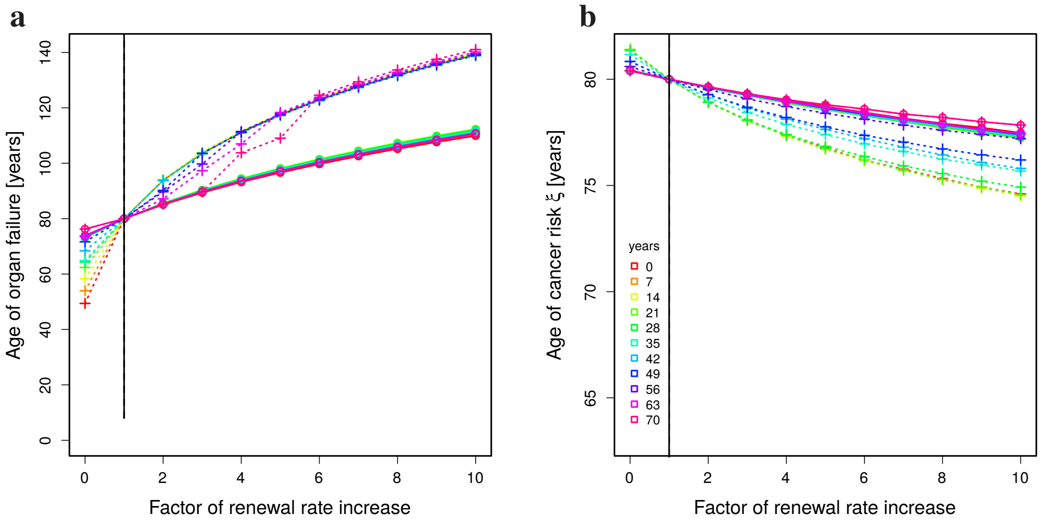

Supplementary Figure 15: Supplementary Figure 4 with age-dependent damage rate Eq. (16). Organ turnover rate $\delta = 0.023$/year of age-independent treatment success. Relative improvement of renewal rate during treatment.

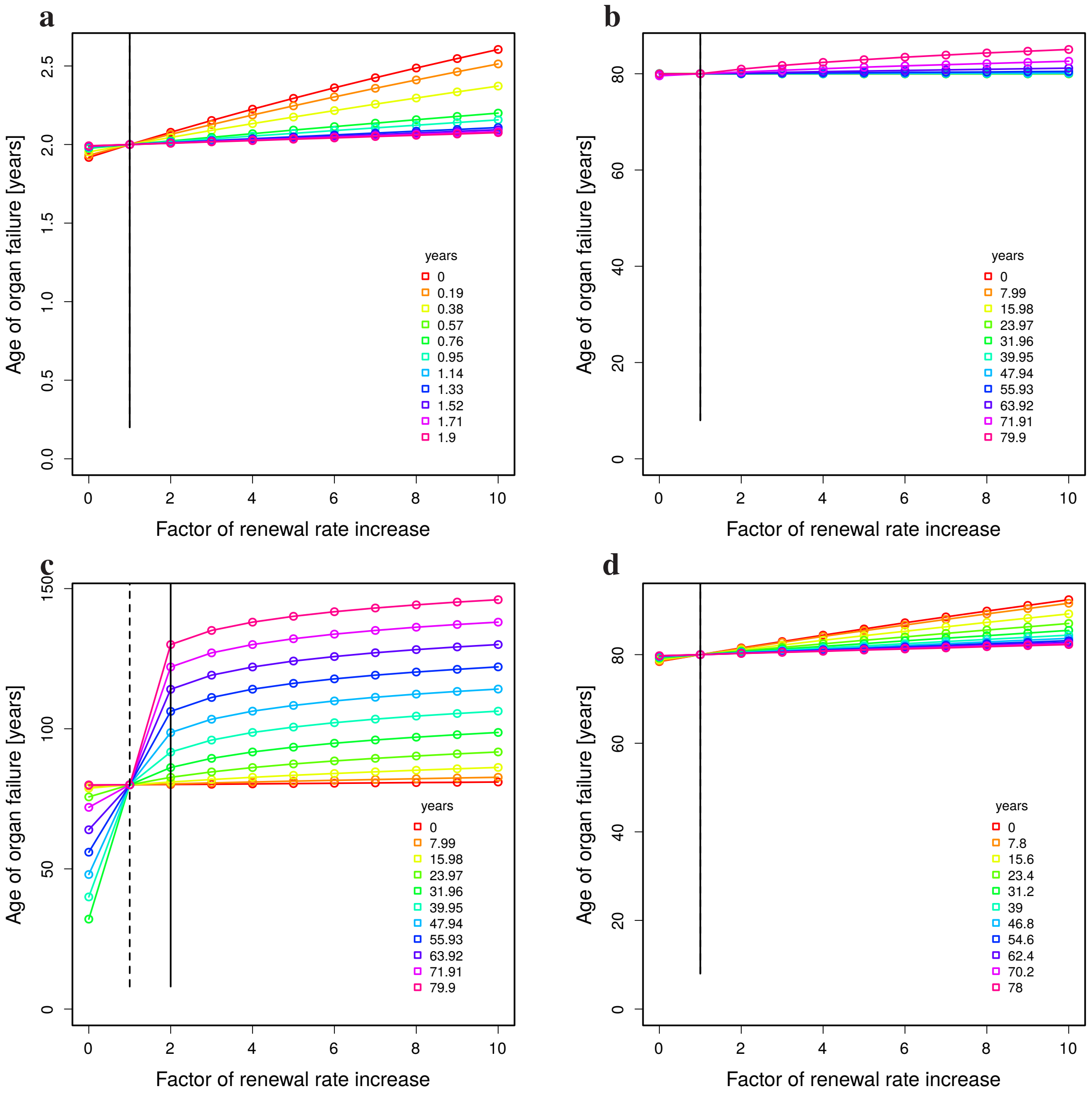

Supplementary Figure 16: Supplementary Figure 5 with age-dependent damage rate Eq. (16). **(a)** Mouse, age-dependent renewal, treated 5 weeks. **(b)** Human, age-dependent renewal, high turnover organ, treated 5 weeks. **(c)** Human, age-independent renewal, high turnover organ, treated 5 weeks. **(d)** Human, age-dependent renewal, low turnover organ, treated 2 years. Relative improvement of the renewal rate during treatment.

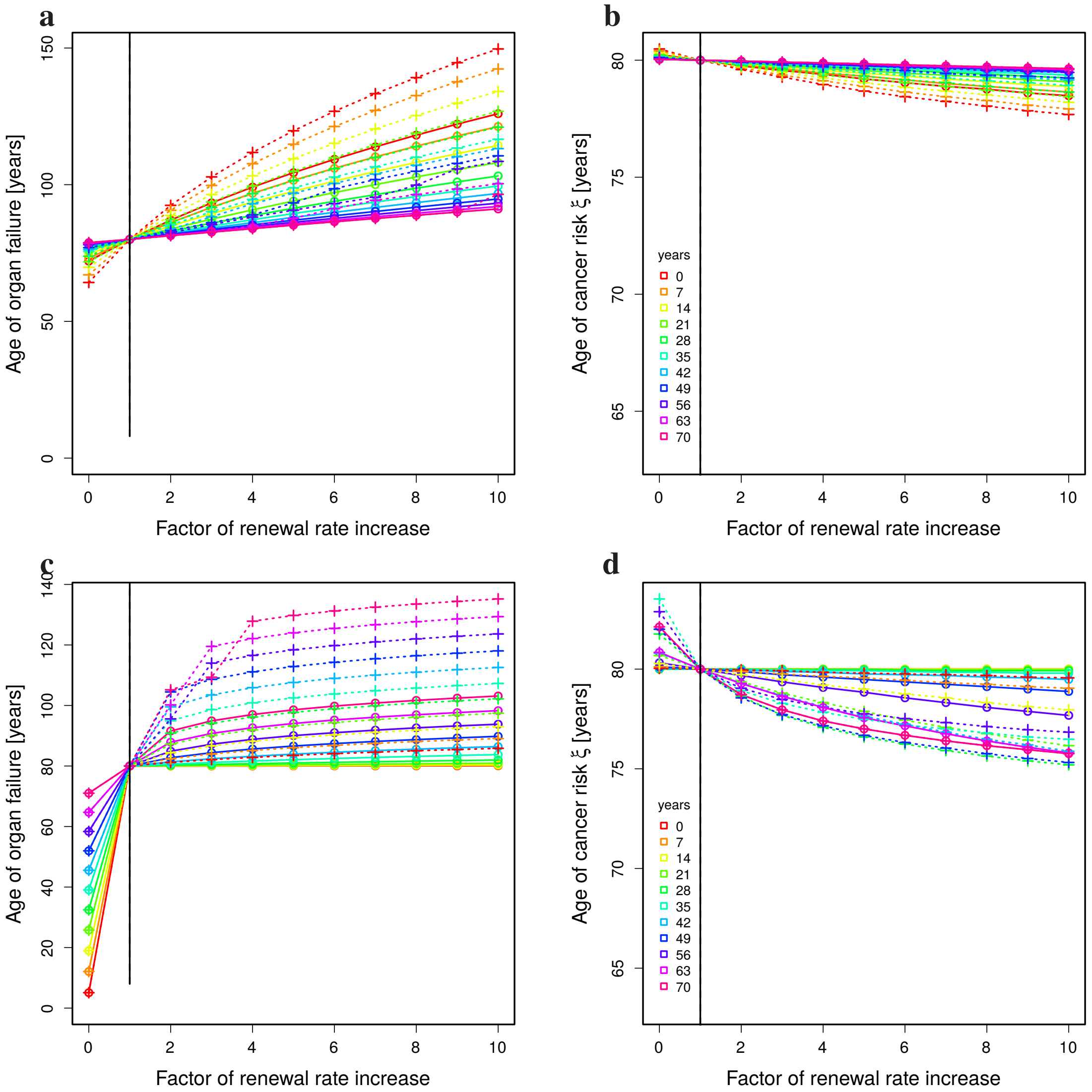

Supplementary Figure 17: Age-dependent damage rate Eq. (16). Same analysis and representation as in Figure 11c,d for low **(a,b)** and in Figure 14c,d for high **(c,d)** turnover organs with a cancer-inducing number of damage events of $c = 20$.

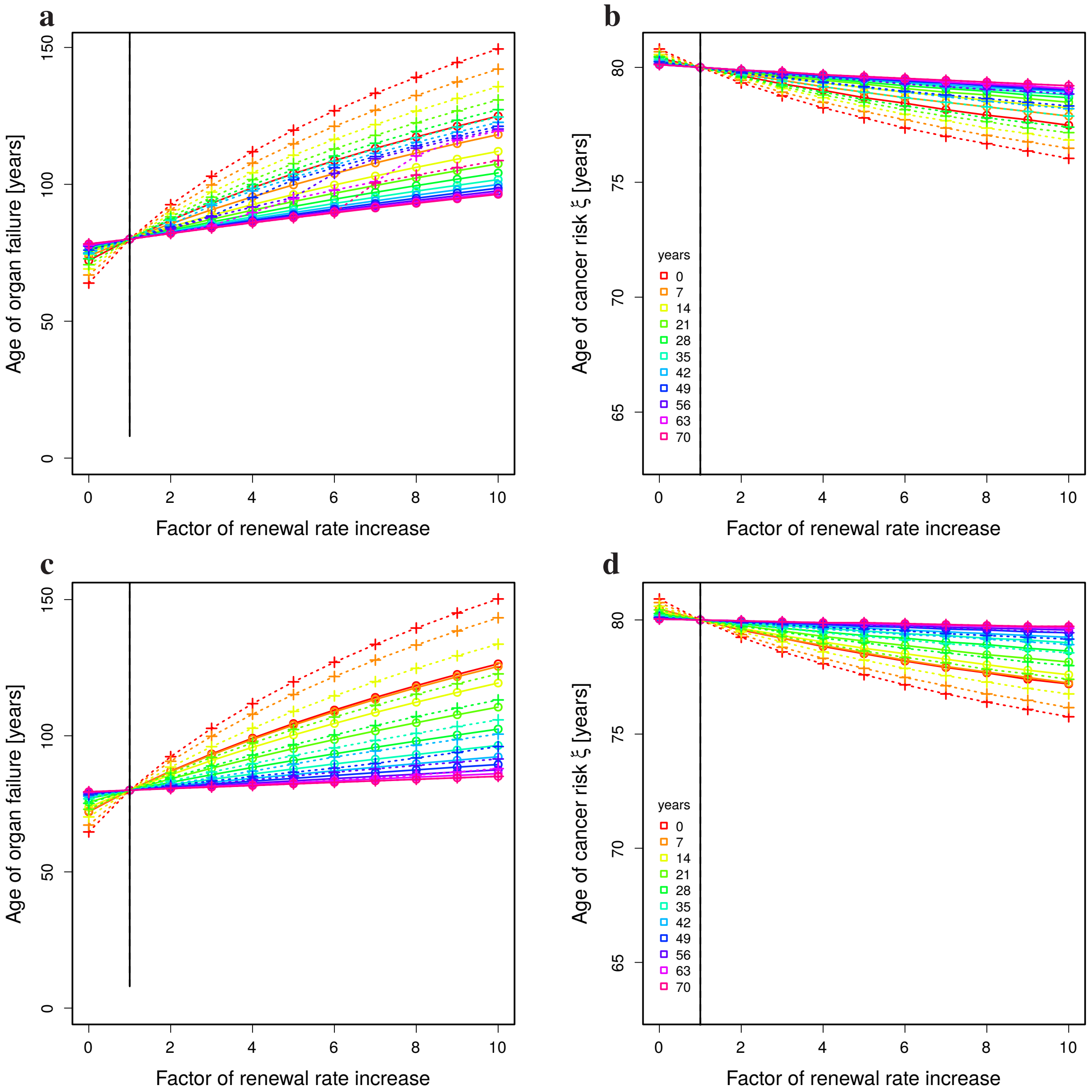

Supplementary Figure 18: Age-dependent damage rate Eq. (16). Same analysis and representation as in Figure 11c,d with flat ($n_\mathrm{p} = 1.5$, **a,b**) and steep ($n_\mathrm{p} = 3$, **c,d**) age-dependence of the division rate in Eq. (15). Parameters of low turnover rates (see Table 1).

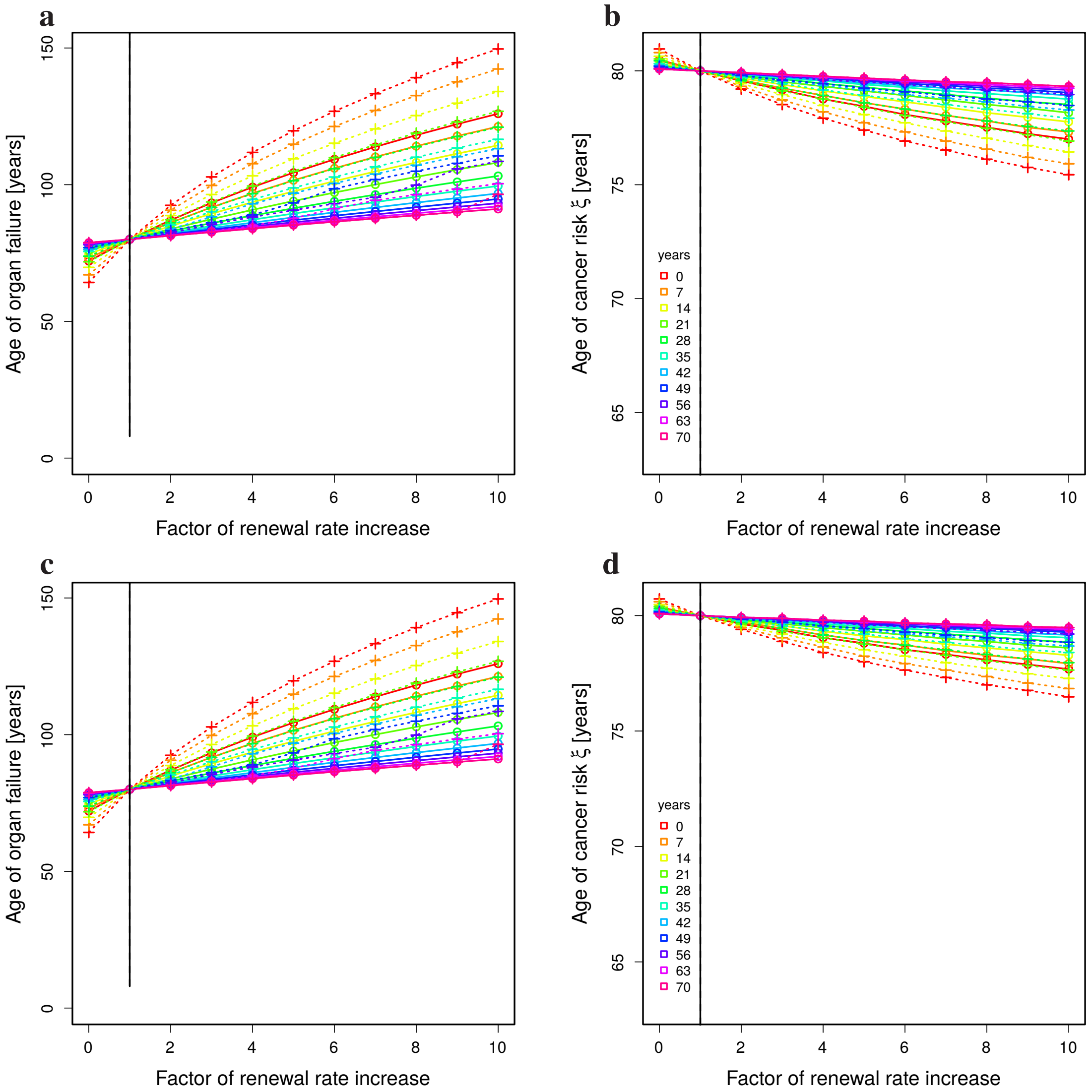

Supplementary Figure 19: Age-dependent damage rate Eq. (16). Same analysis and representation as in Figure 11c,d with flat ($n_\gamma = 1.5$, **a,b**) and steep ($n_\gamma = 3$, **c,d**) age-dependence of the damage rate in Eq. (16). Parameters of low turnover rates (see Table 1).

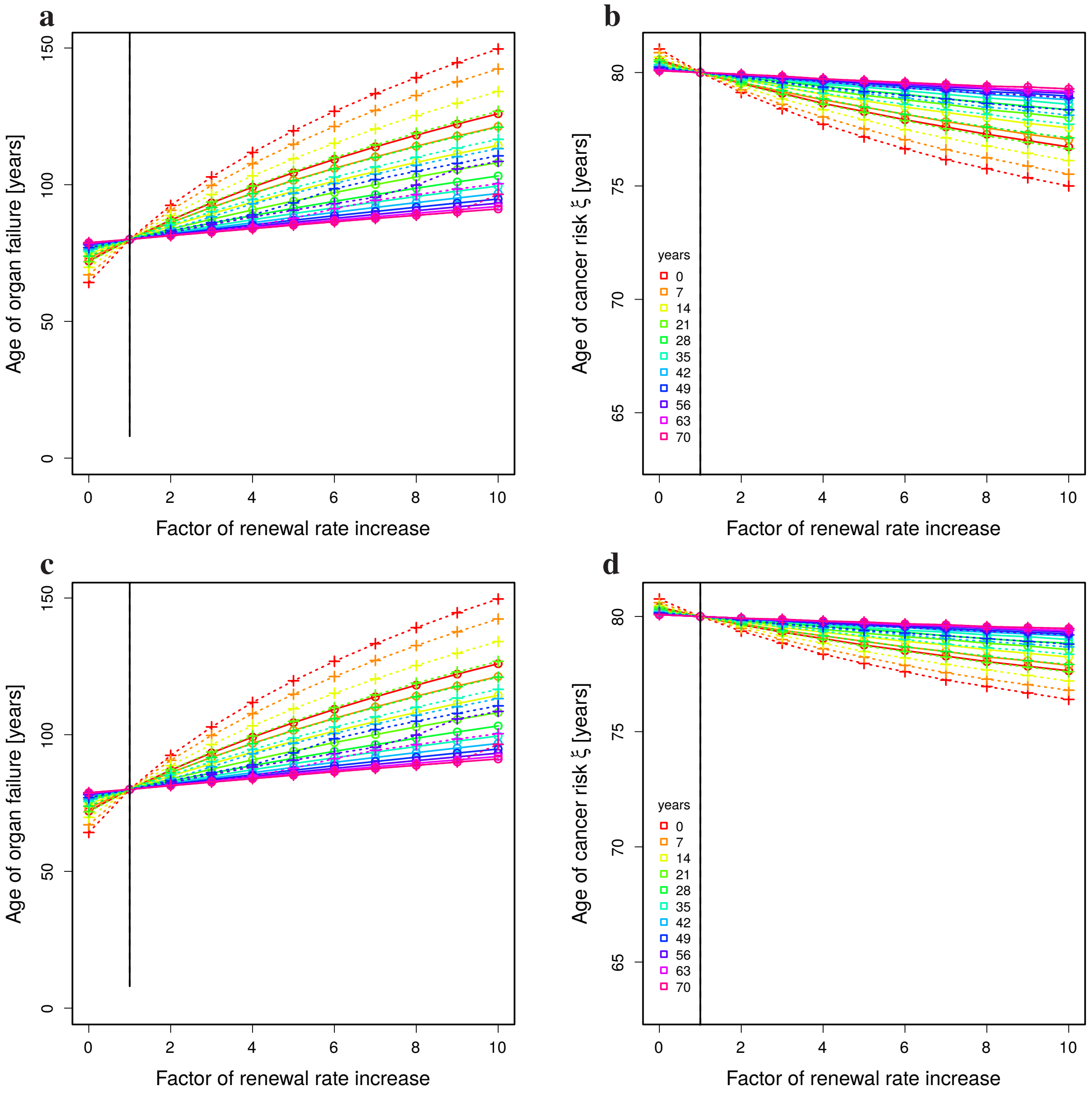

Supplementary Figure 20: Age-dependent damage rate Eq. (16). Same analysis and representation as in Figure 11c,d with young ($K_\gamma = 30$, **a,b**) and old ($K_\gamma = 70$, **c,d**) age for half maximum damage rate in Eq. (16). Parameters of low turnover rates (see Table 1).

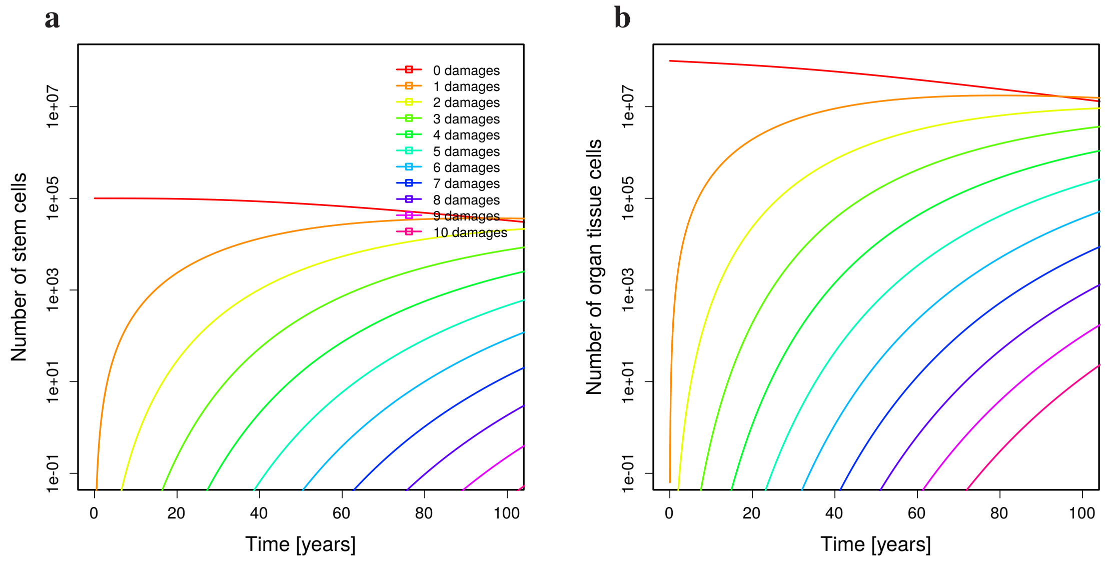

Supplementary Figure 21: Figure 1 with age- and division-dependent damage rate Eqs. (16) and (17). Low turnover parameter in Table 1.

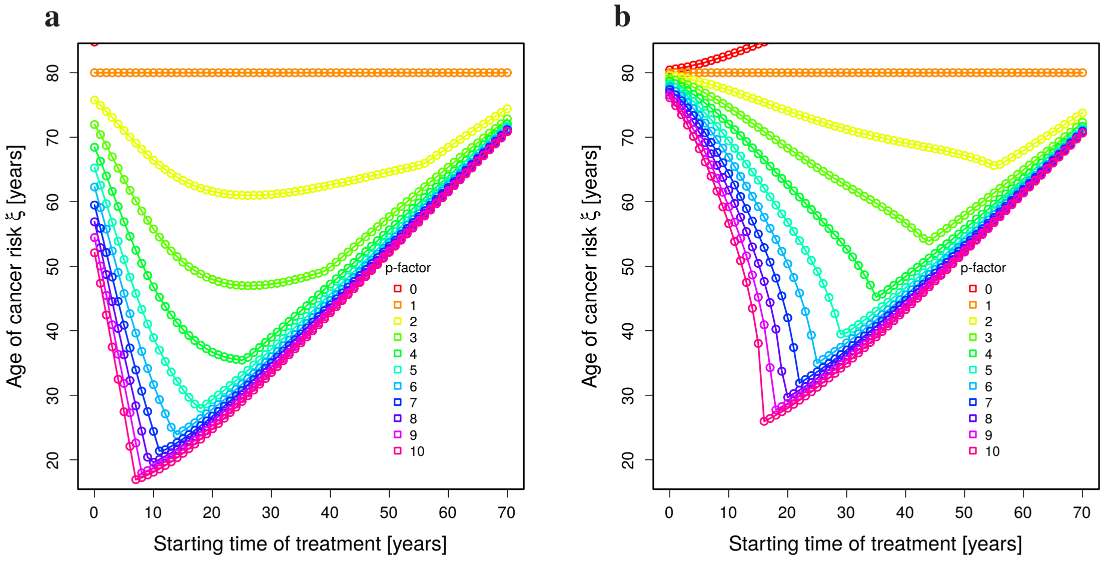

Supplementary Figure 22: Figure 4c,d with age- and division-dependent damage rate Eqs. (16) and (17). Low (a) and high (b) turnover parameter in Table 1.

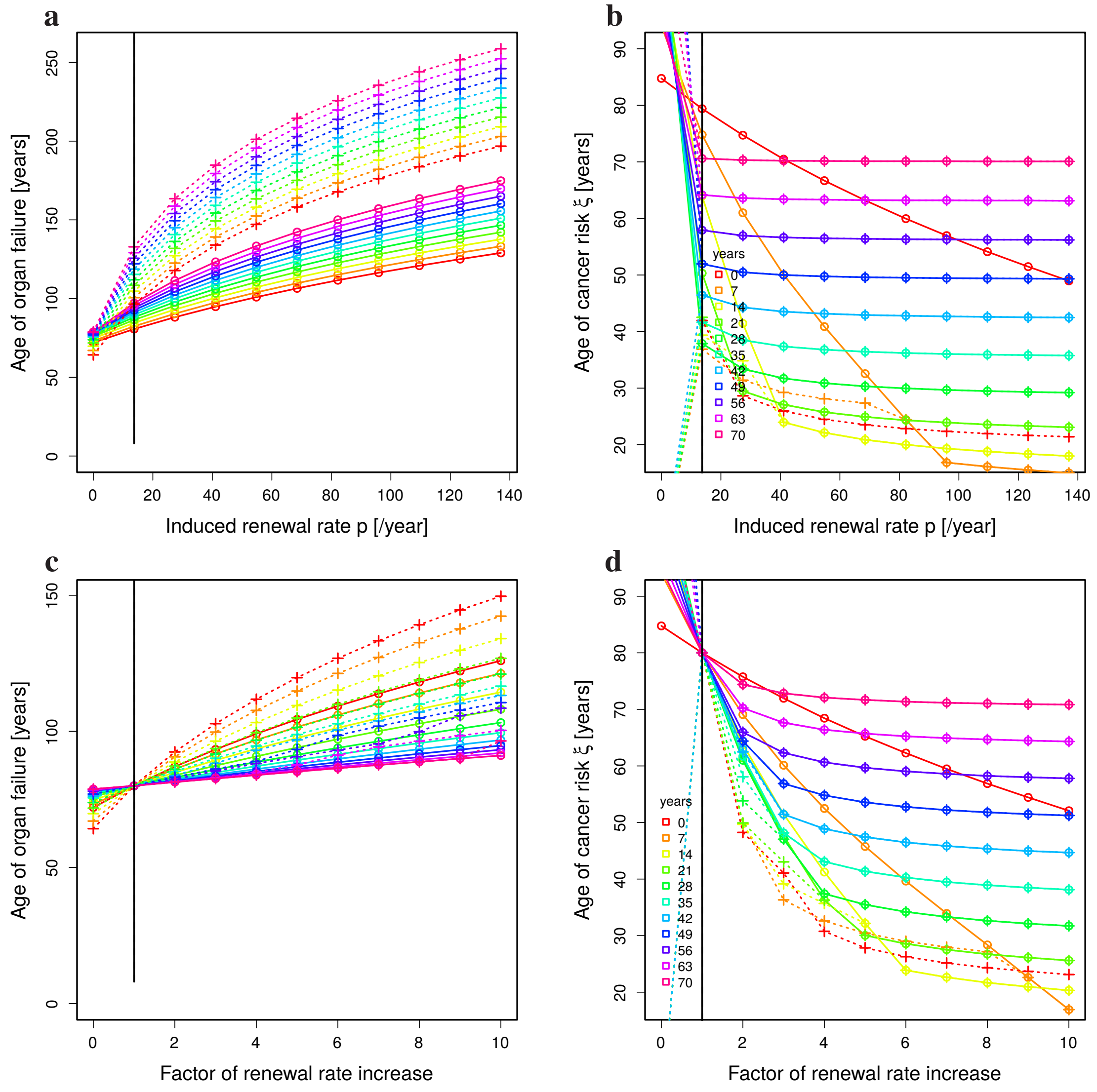

Supplementary Figure 23: Figure 5 with age- and division-dependent damage rate Eqs. (16) and (17). Low turnover parameter in Table 1. Absolute **(a,b)** or relative **(c,d)** improvement of the renewal rate during treatment.

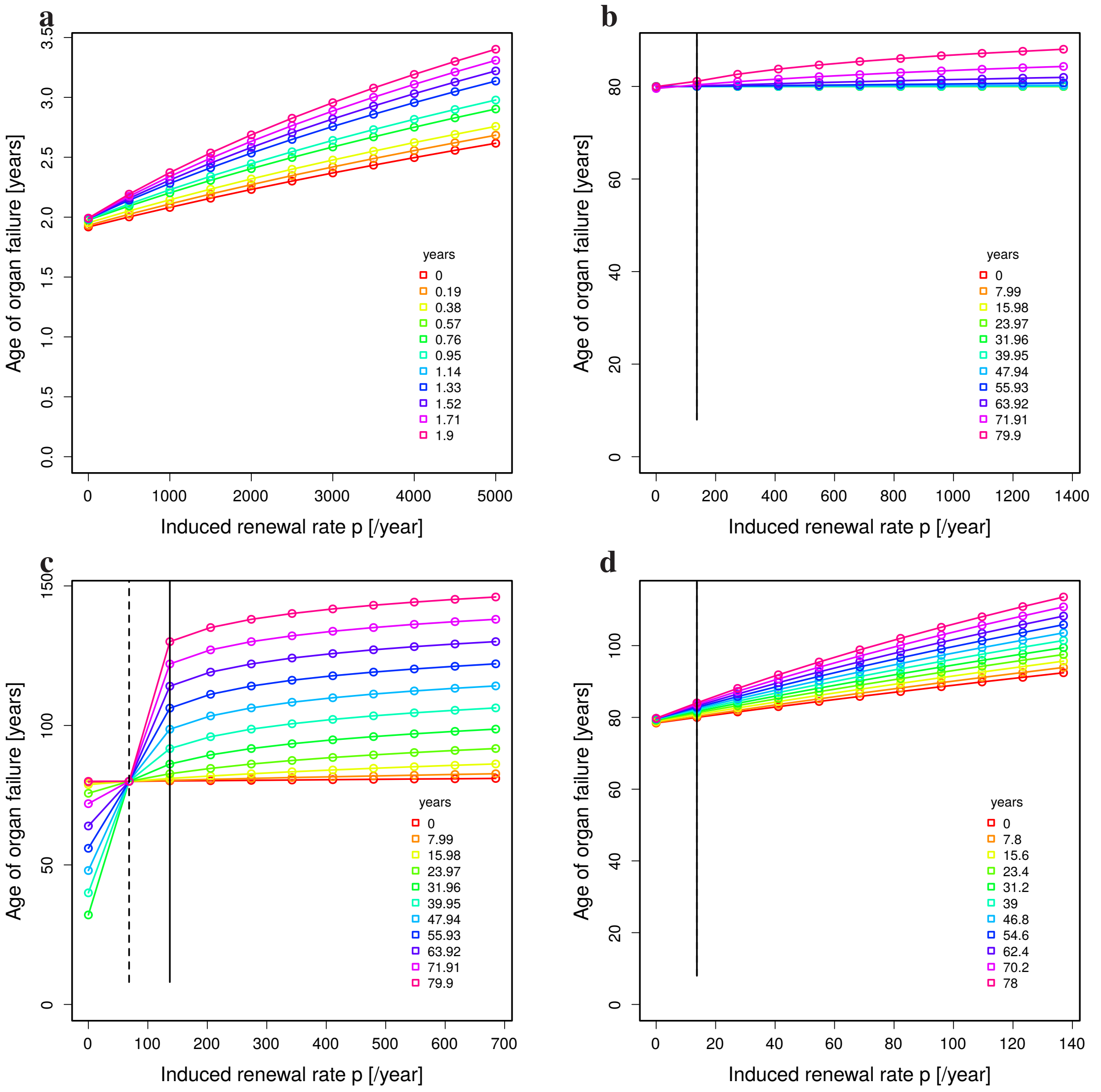

Supplementary Figure 24: Figure 7 with age- and division-dependent damage rate Eqs. (16) and (17). **(a)** Mouse, age-dependent renewal, treated 5 weeks. **(b)** Human, age-dependent renewal, high turnover organ, treated 5 weeks. **(c)** Human, age-independent renewal, high turnover organ, treated 5 weeks. **(d)** Human, age-dependent renewal, low turnover organ, treated 2 years. Absolute improvement of the renewal rate during treatment.

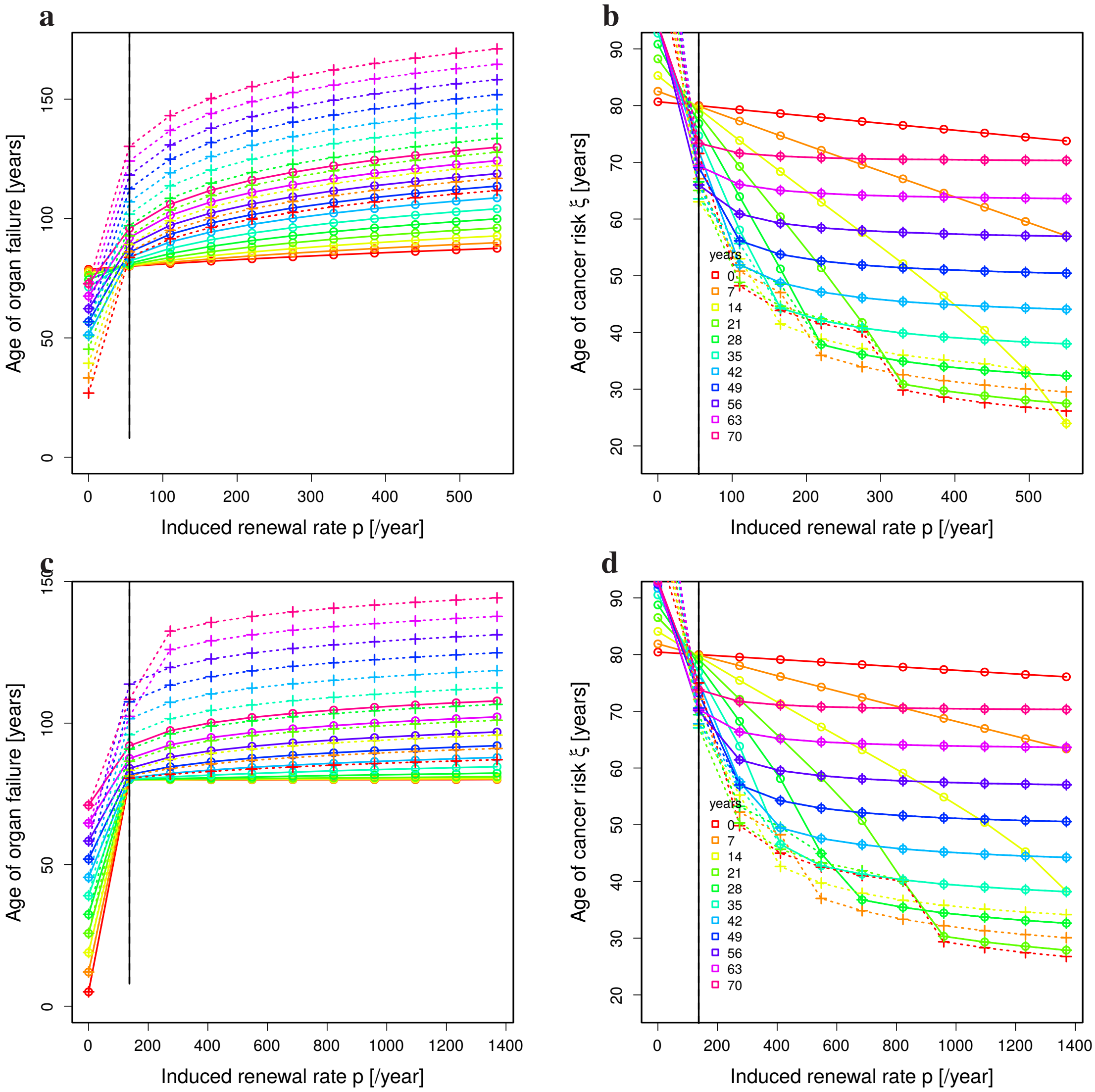

Supplementary Figure 25: Supplementary Figure 2 with age- and division-dependent damage rate Eqs. (16) and (17). Intermediate **(a,b)** or high **(c,d)** turnover parameter in Table 1. Absolute improvement of renewal rate during treatment.

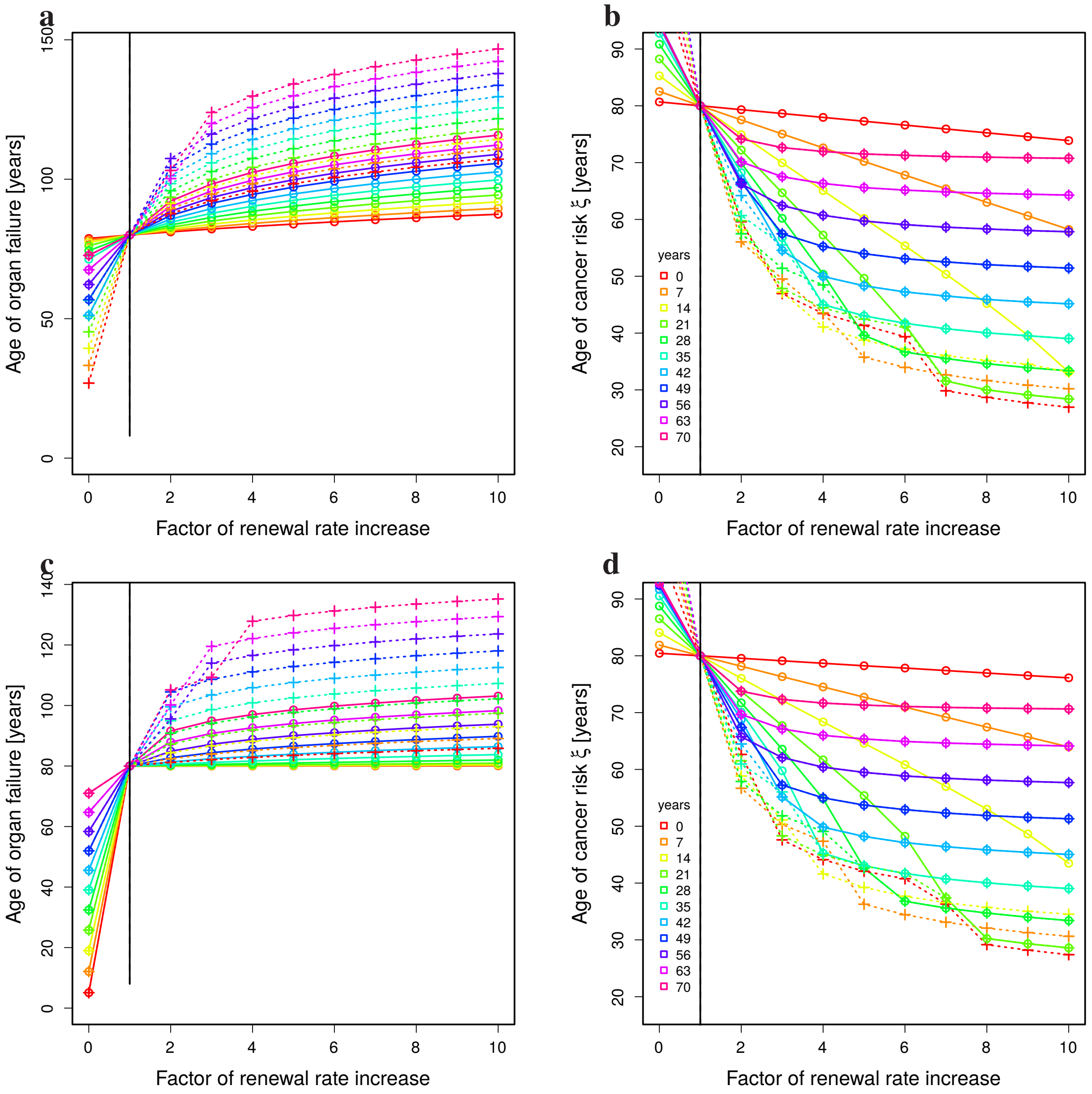

Supplementary Figure 26: Supplementary Figure 3 with age- and division-dependent damage rate Eqs. (16) and (17). Intermediate **(a,b)** or high **(c,d)** turnover parameter in Table 1. Relative improvement of renewal rate during treatment.

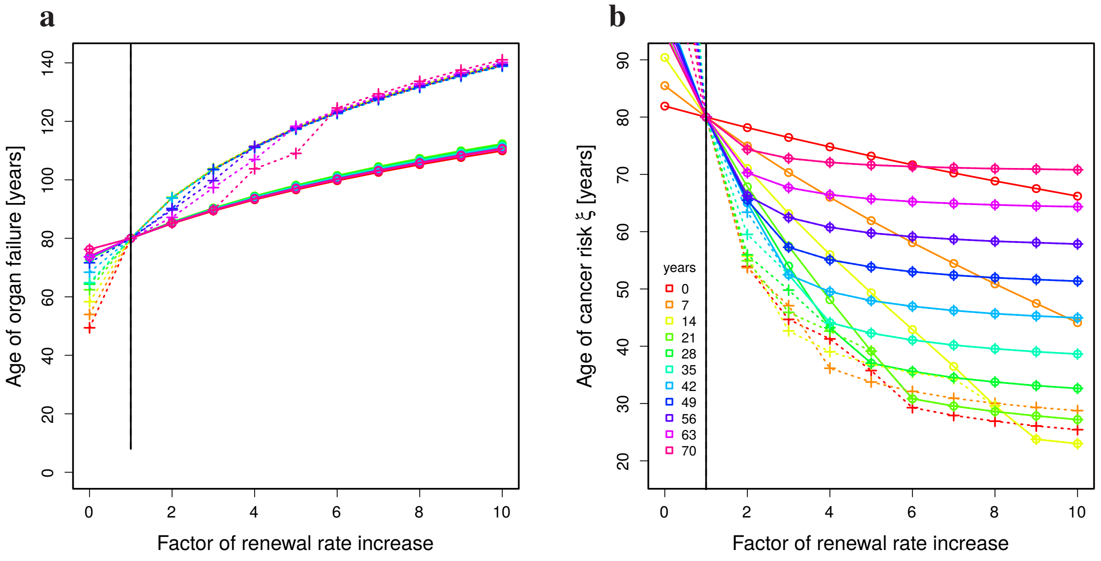

Supplementary Figure 27: Supplementary Figure 4 with age- and division-dependent damage rate Eqs. (16) and (17). Organ turnover rate $\delta = 0.023$/year of age-independent treatment success. Relative improvement of renewal rate during treatment.

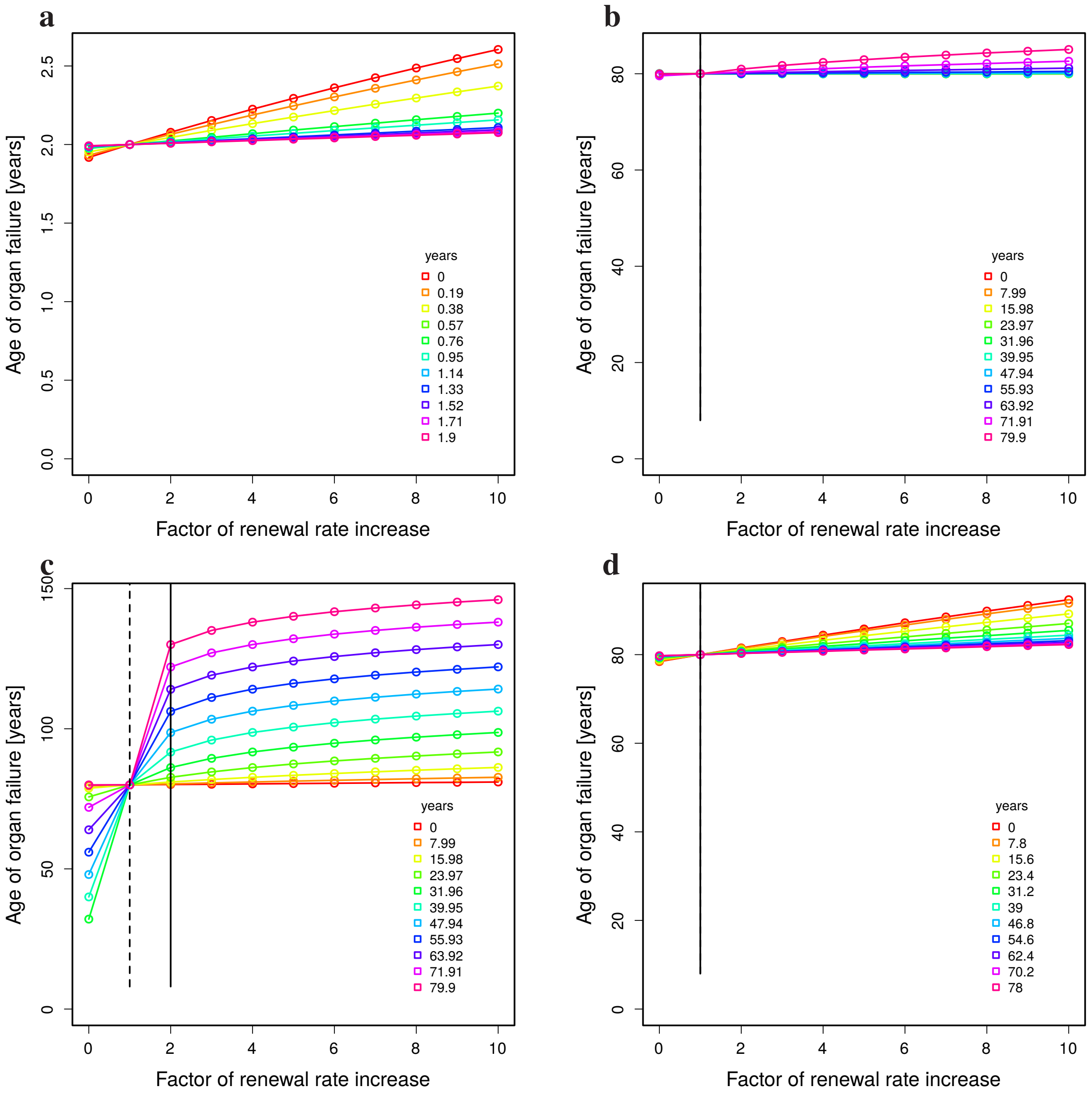

Supplementary Figure 28: Supplementary Figure 5 with age- and division-dependent damage rate Eqs. (16) and (17). **(a)** Mouse, age-dependent renewal, treated 5 weeks. **(b)** Human, age-dependent renewal, high turnover organ, treated 5 weeks. **(c)** Human, age-independent renewal, high turnover organ, treated 5 weeks. **(d)** Human, age-dependent renewal, low turnover organ, treated 2 years. Relative improvement of the renewal rate during treatment.

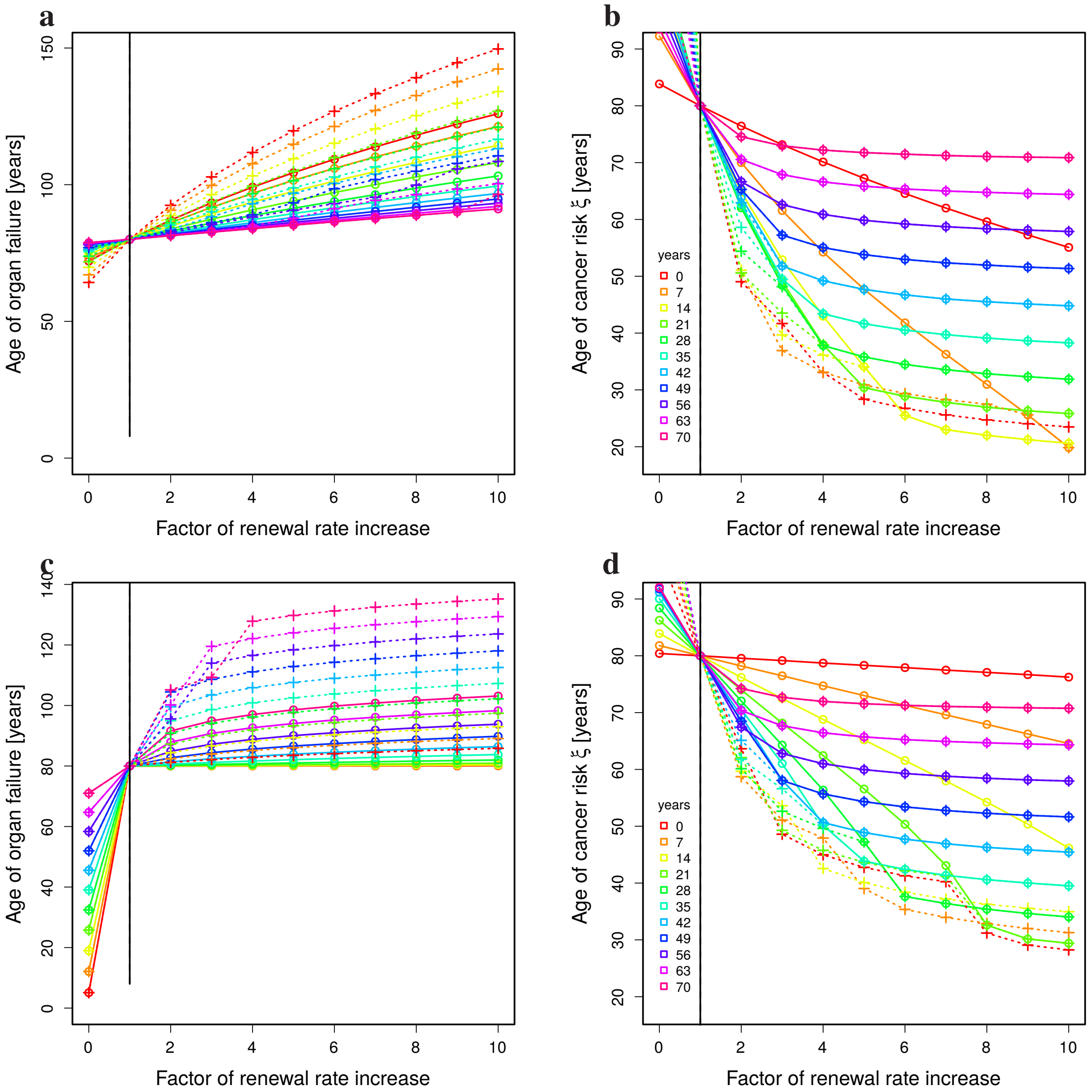

Supplementary Figure 29: Age- and division-dependent damage rate Eqs. (16) and (17). Same analysis and representation as in Figure 23c,d for low **(a,b)** and in Figure 26c,d for high **(c,d)** turnover organs with a cancer-inducing number of damage events of $c = 20$.

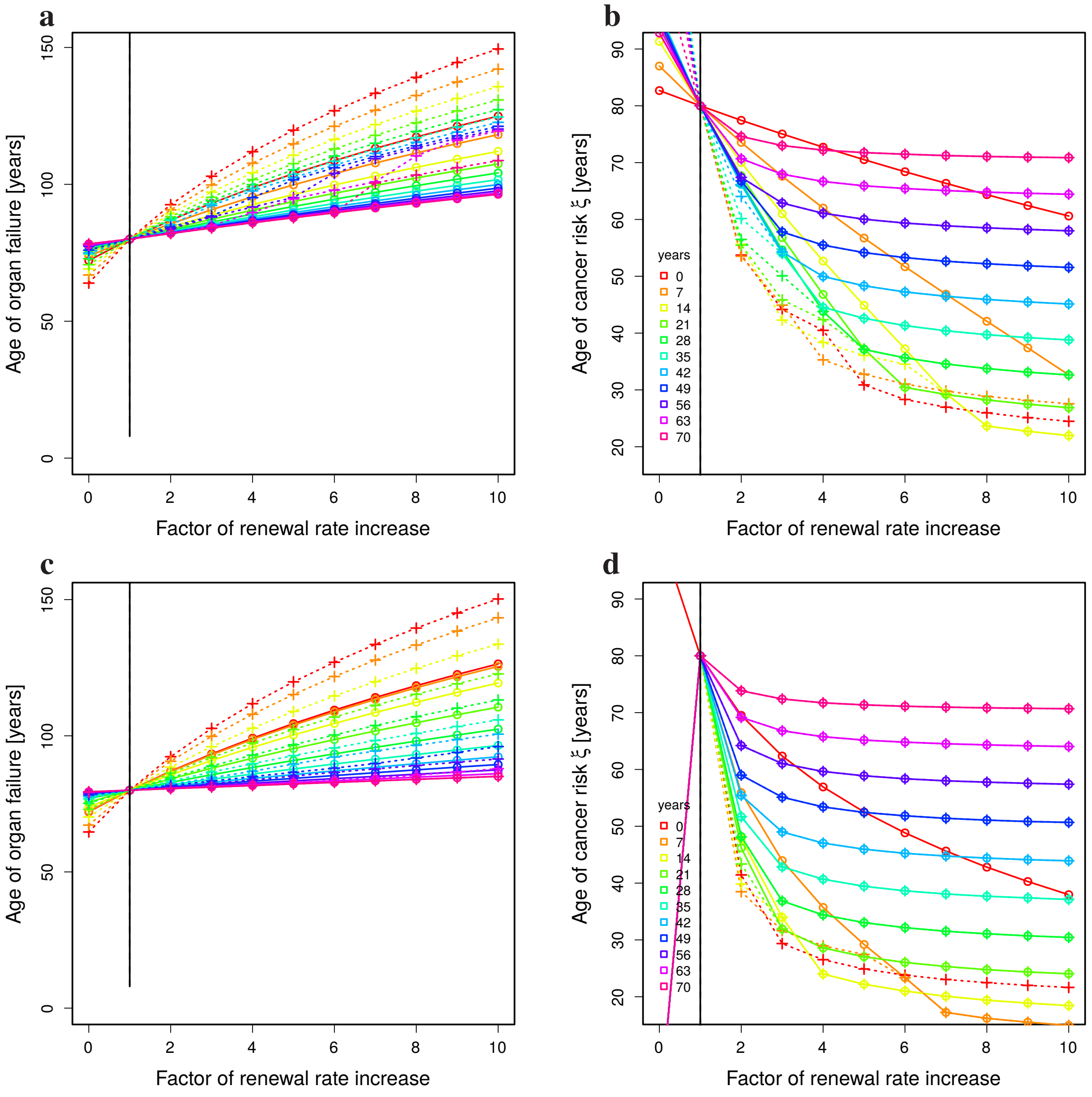

Supplementary Figure 30: Age- and division-dependent damage rate Eqs. (16) and (17). Same analysis and representation as in Figure 23c,d with flat ($n_\mathrm{p} = 1.5$, **a,b**) and steep ($n_\mathrm{p} = 3$, **c,d**) age-dependence of the division rate in Eq. (15). Parameters of low turnover rates (see Table 1).

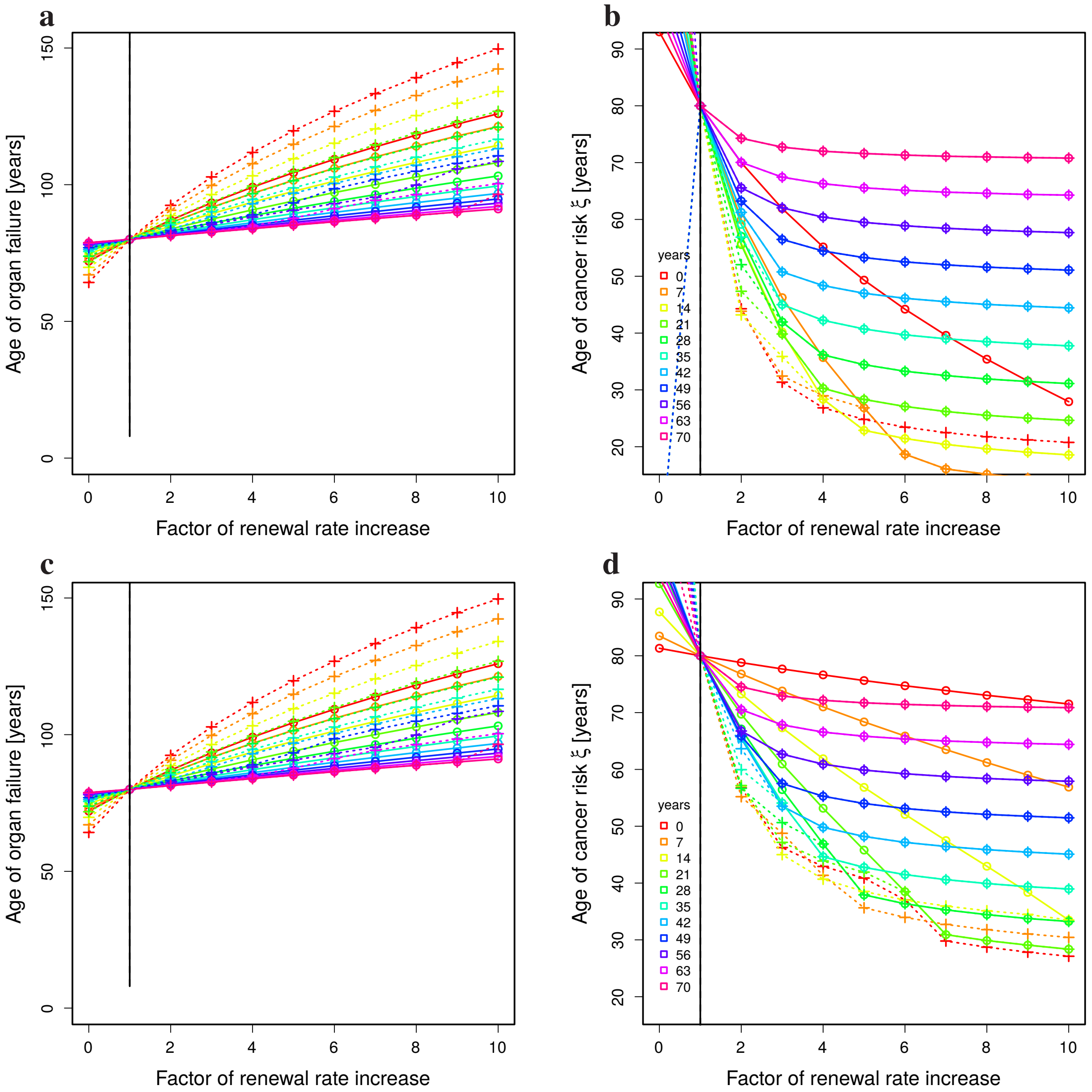

Supplementary Figure 31: Age- and division-dependent damage rate Eqs. (16) and (17). Same analysis and representation as in Figure 23c,d with flat ($n_\gamma = 1.5$, **a,b**) and steep ($n_\gamma = 3$, **c,d**) age-dependence of the damage rate in Eq. (16). Parameters of low turnover rates (see Table 1).

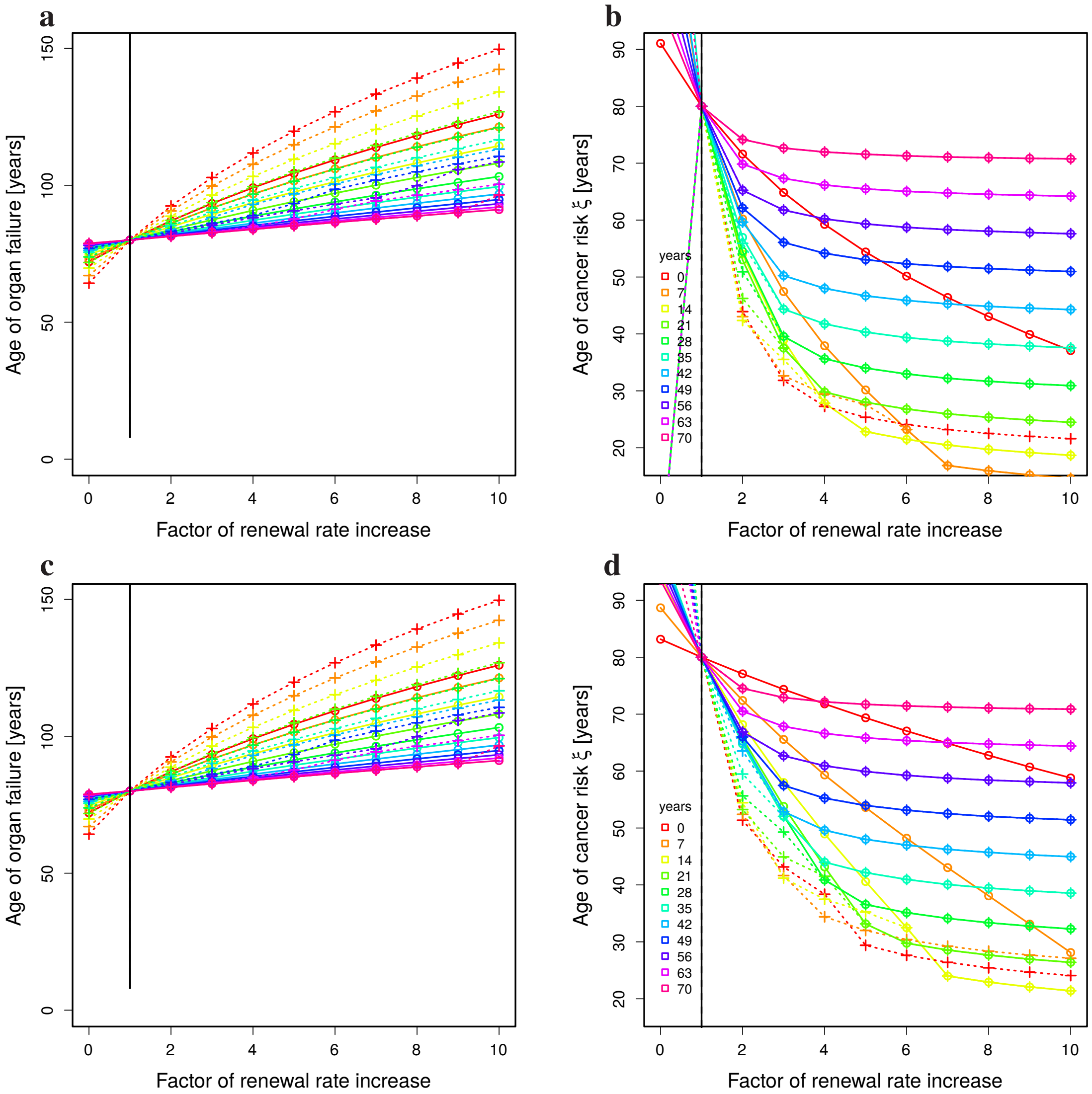

Supplementary Figure 32: Age- and division-dependent damage rate Eqs. (16) and (17). Same analysis and representation as in Figure 23c,d with young ($K_\gamma = 30$, **a,b**) and old ($K_\gamma = 70$, **c,d**) age for half maximum damage rate in Eq. (16). Parameters of low turnover rates (see Table 1).

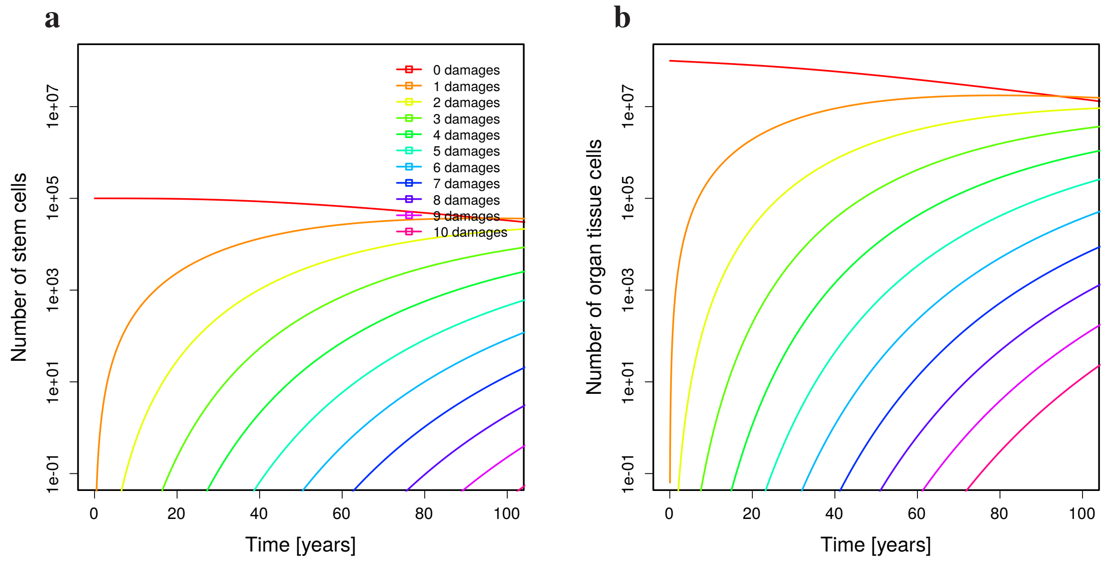

Supplementary Figure 33: Figure 1 with age- and division-dependent damage rate and damage rate kept age-specific during treatment Eqs. (16) and (17). Low turnover parameter in Table 1.

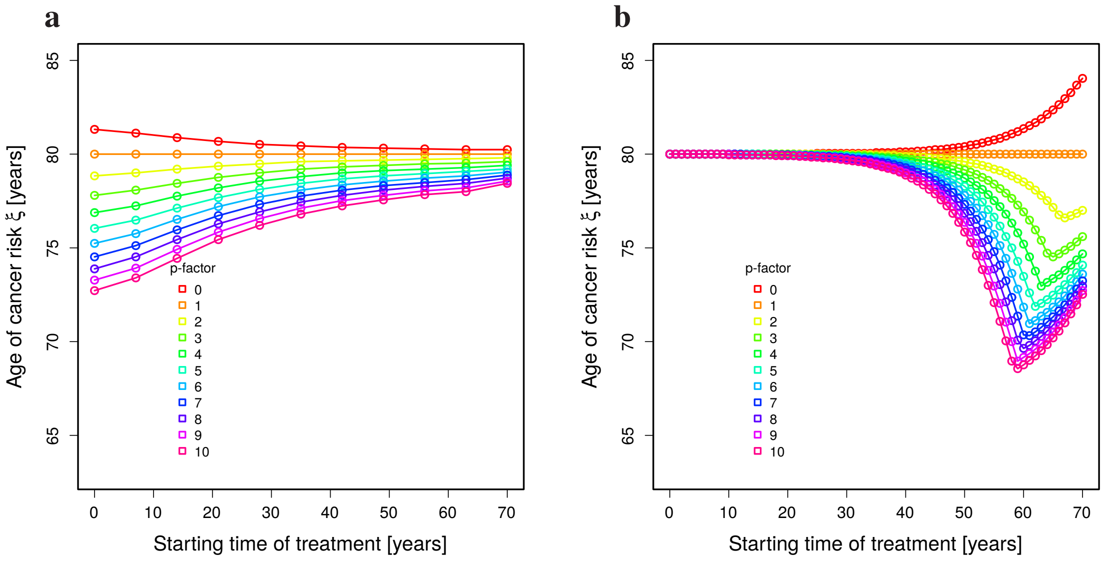

Supplementary Figure 34: Figure 4c,d with age- and division-dependent damage rate and damage rate kept age-specific during treatment Eqs. (16) and (17). Low (a) and high (b) turnover parameter in Table 1.

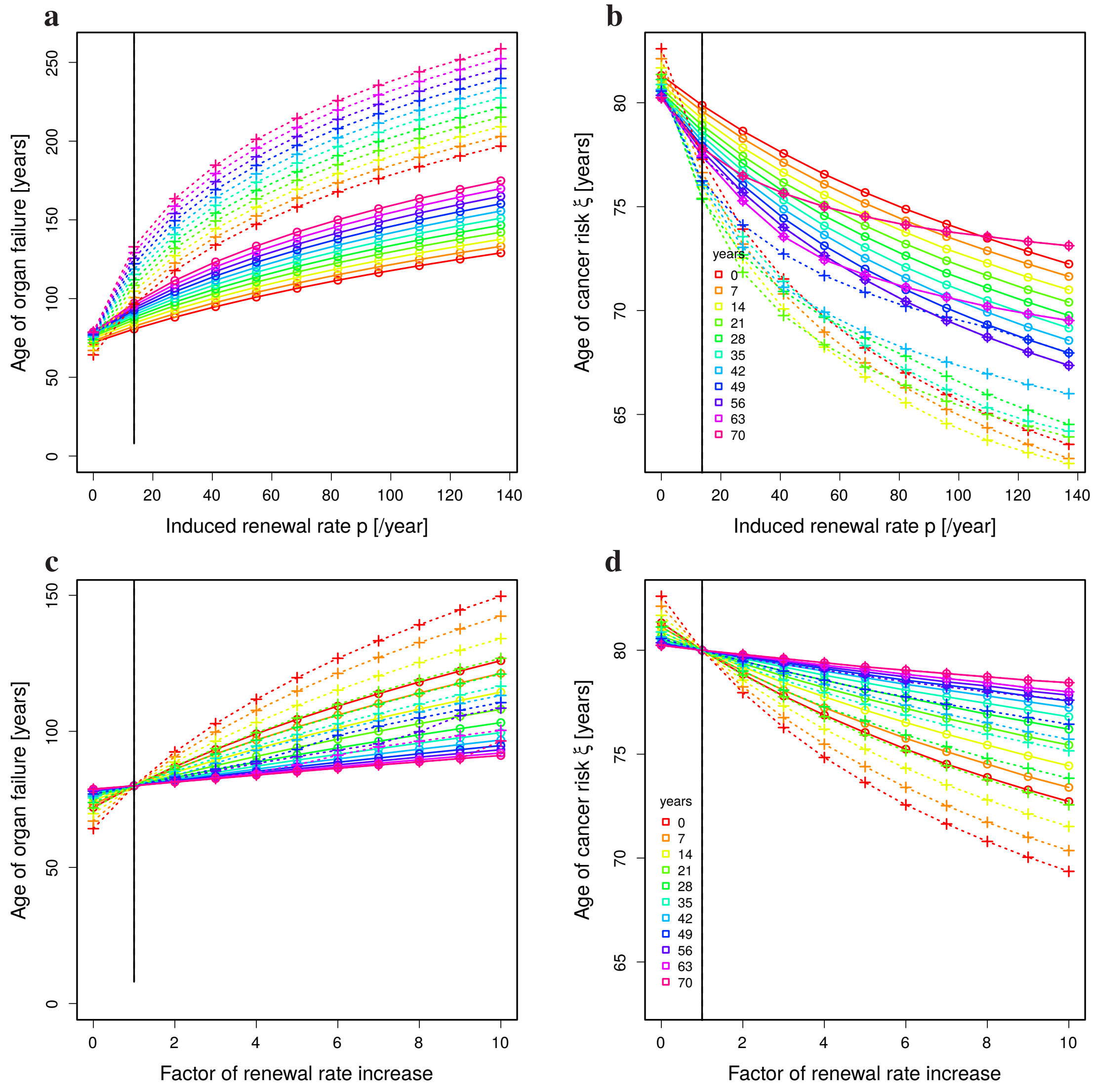

Supplementary Figure 35: Figure 5 with age- and division-dependent damage rate and damage rate kept age-specific during treatment Eqs. (16) and (17). Low turnover parameter in Table 1. Absolute **(a,b)** or relative **(c,d)** improvement of the renewal rate during treatment.

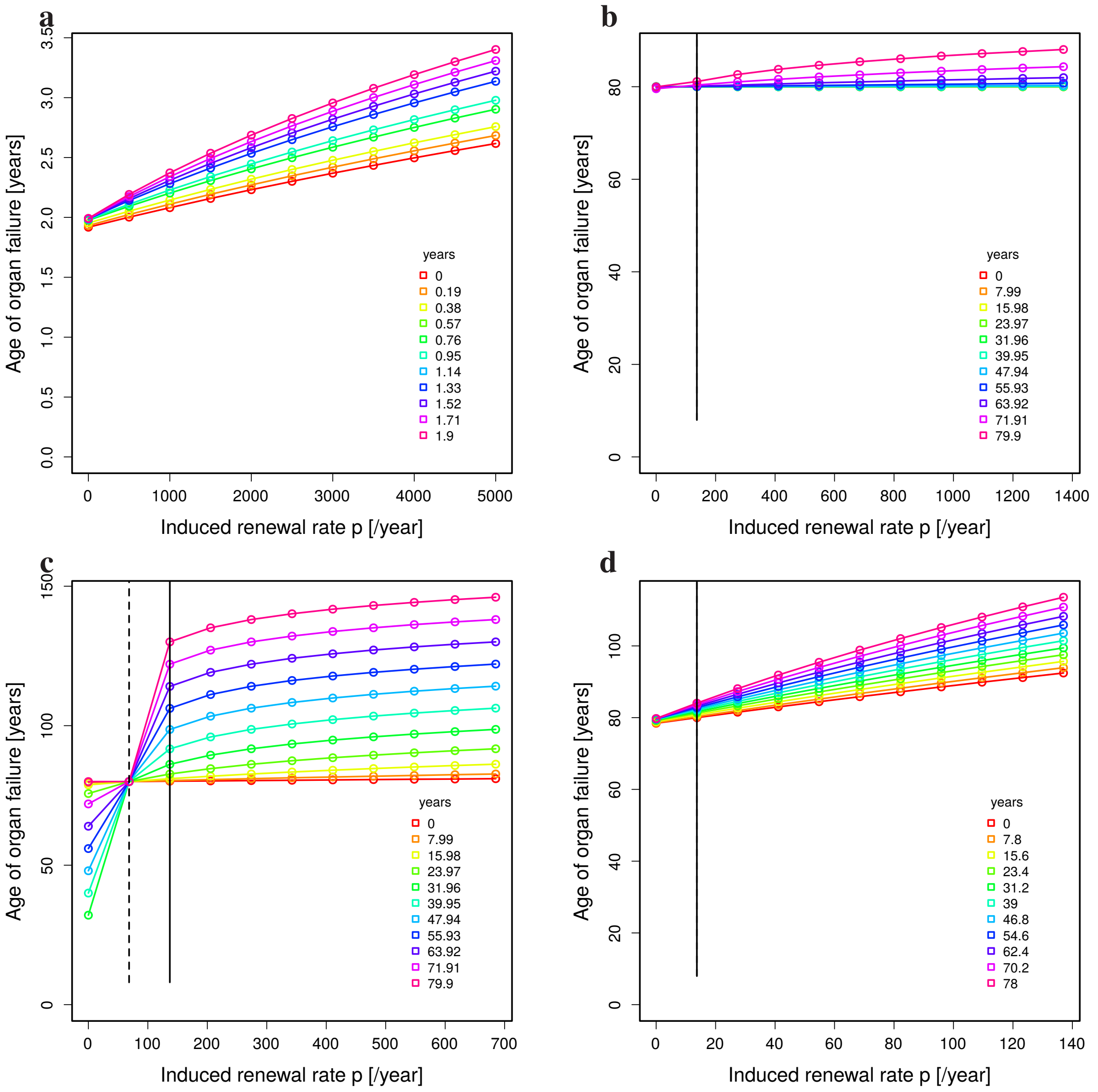

Supplementary Figure 36: Figure 7 with age- and division-dependent damage rate and damage rate kept age-specific during treatment Eqs. (16) and (17). **(a)** Mouse, age-dependent renewal, treated 5 weeks. **(b)** Human, age-dependent renewal, high turnover organ, treated 5 weeks. **(c)** Human, age-independent renewal, high turnover organ, treated 5 weeks. **(d)** Human, age-dependent renewal, low turnover organ, treated 2 years. Absolute improvement of the renewal rate during treatment.

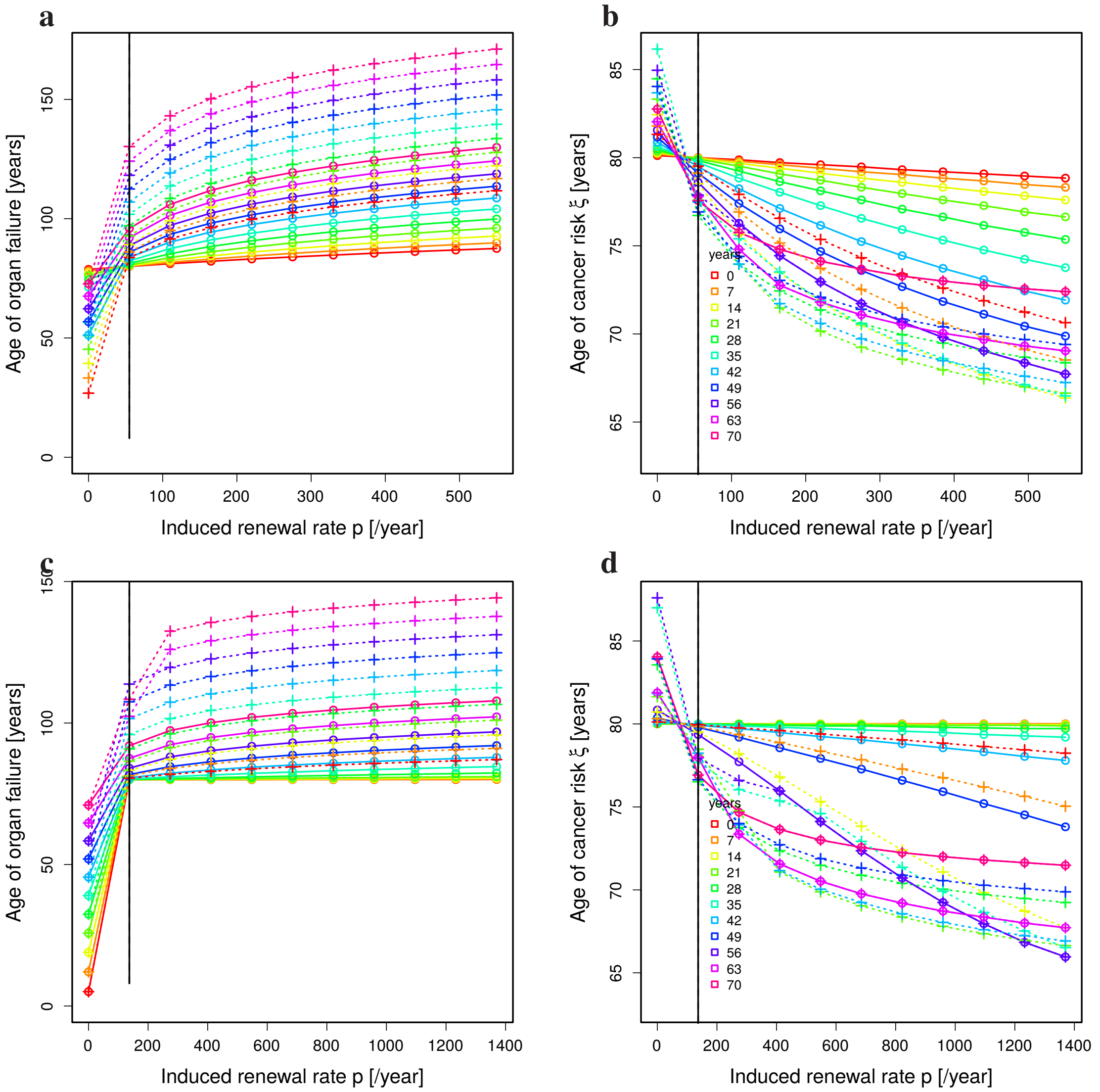

Supplementary Figure 37: Supplementary Figure 2 with age- and division-dependent damage rate and damage rate kept age-specific during treatment Eqs. (16) and (17). Intermediate **(a,b)** or high **(c,d)** turnover parameter in Table 1. Absolute improvement of renewal rate during treatment.

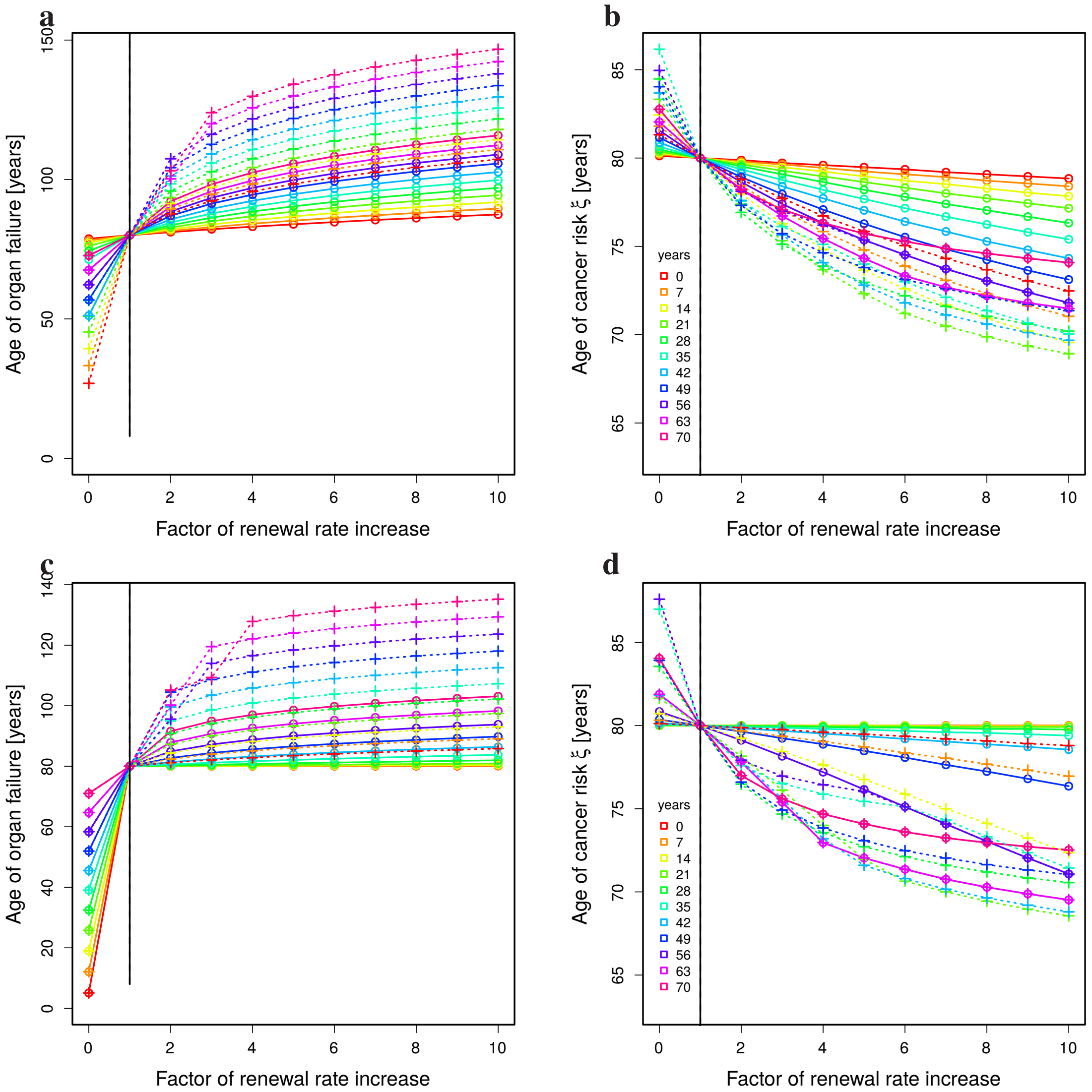

Supplementary Figure 38: Supplementary Figure 3 with age- and division-dependent damage rate and damage rate kept age-specific during treatment Eqs. (16) and (17). Intermediate **(a,b)** or high **(c,d)** turnover parameter in Table 1. Relative improvement of renewal rate during treatment.

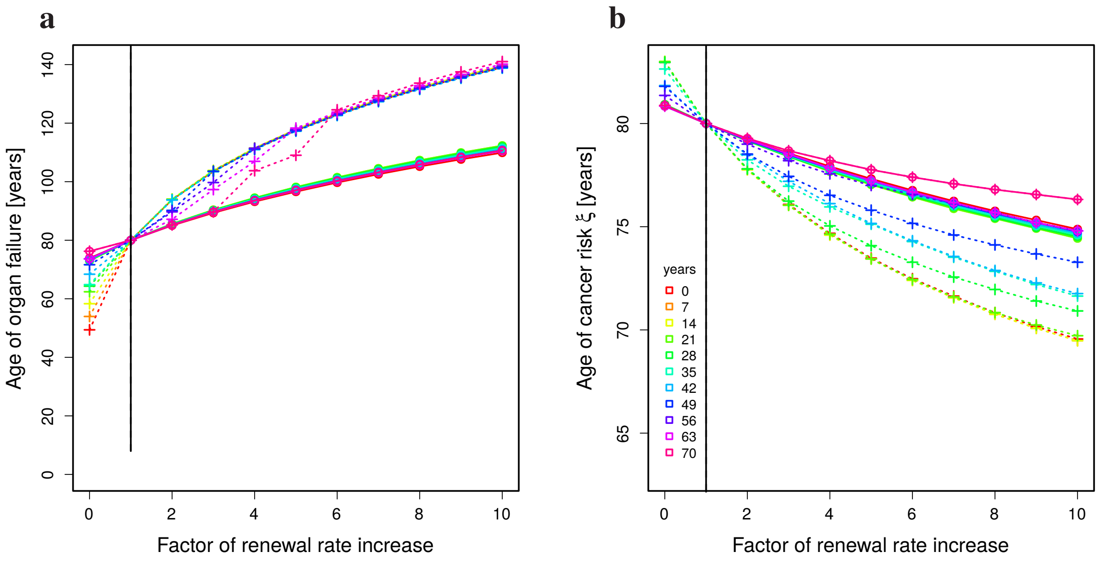

Supplementary Figure 39: Supplementary Figure 4 with age- and division-dependent damage rate and damage rate kept age-specific during treatment Eqs. (16) and (17). Organ turnover rate $\delta = 0.023$/year of age-independent treatment success. Relative improvement of renewal rate during treatment.

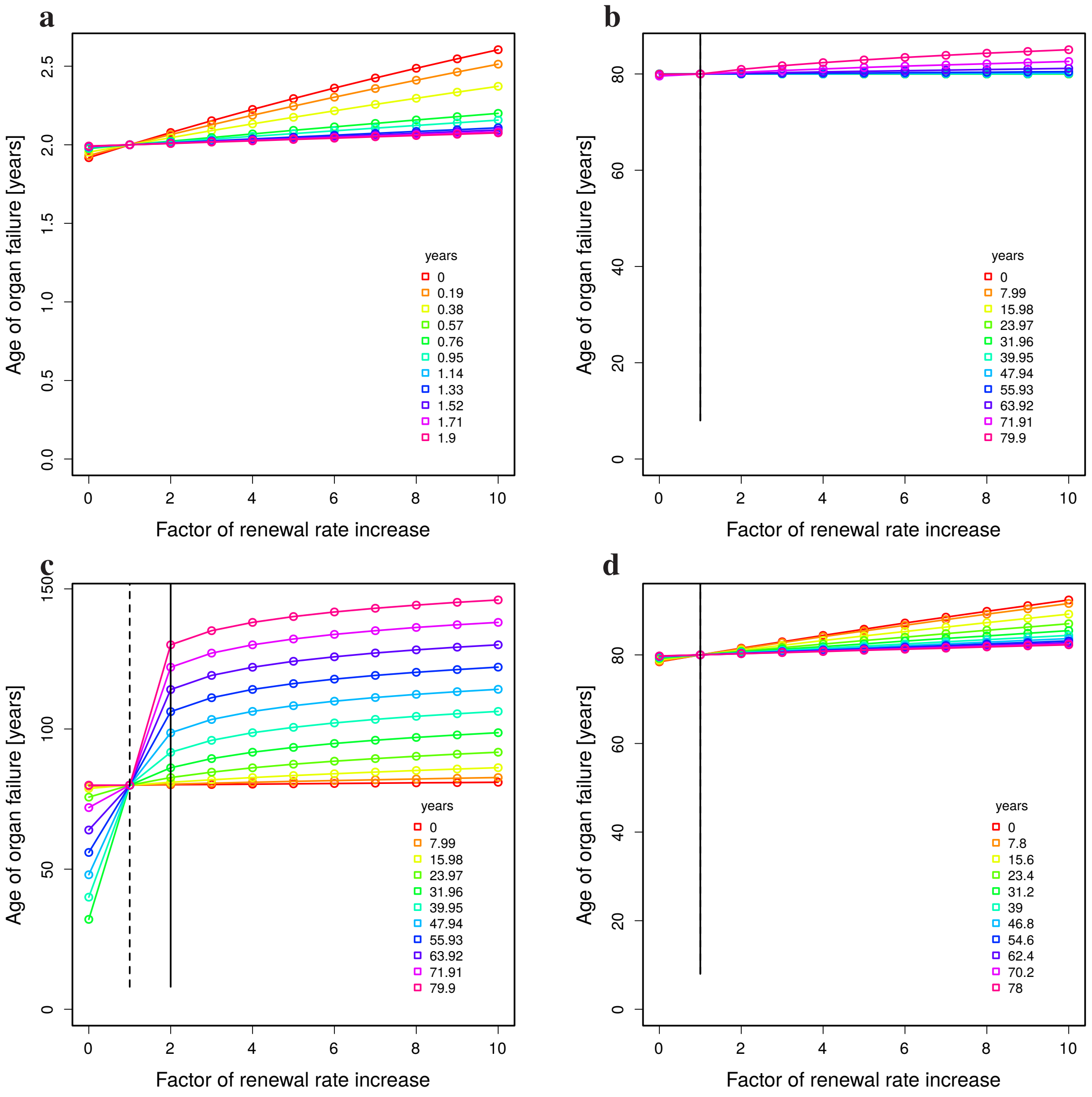

Supplementary Figure 40: Supplementary Figure 5 with age- and division-dependent damage rate and damage rate kept age-specific during treatment Eqs. (16) and (17). **(a)** Mouse, age-dependent renewal, treated 5 weeks. **(b)** Human, age-dependent renewal, high turnover organ, treated 5 weeks. **(c)** Human, age-independent renewal, high turnover organ, treated 5 weeks. **(d)** Human, age-dependent renewal, low turnover organ, treated 2 years. Relative improvement of the renewal rate during treatment.

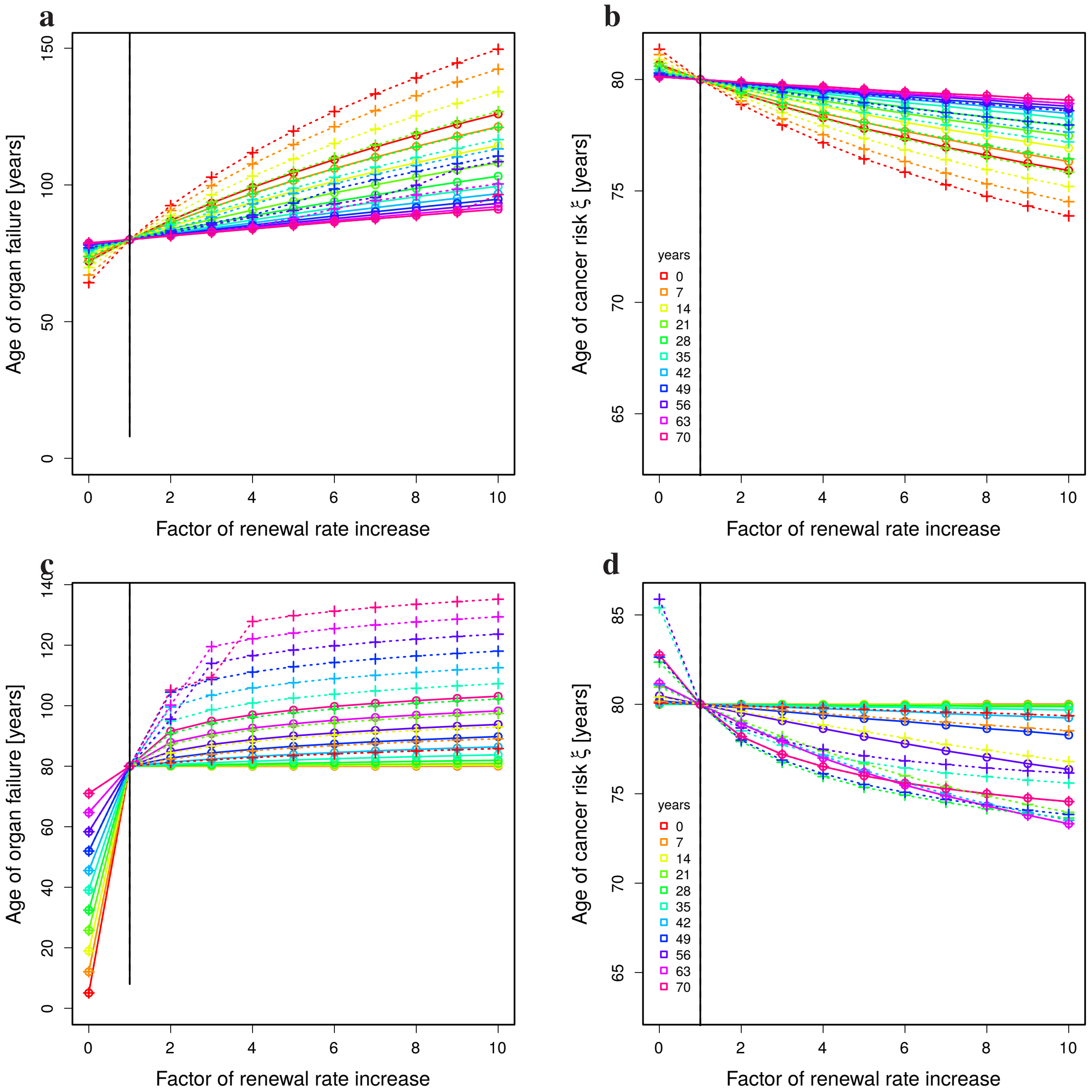

Supplementary Figure 41: Age- and division-dependent damage rate and damage rate kept age-specific during treatment Eqs. (16) and (17). Same analysis and representation as in Figure 35c,d for low **(a,b)** and in Figure 38c,d for high **(c,d)** turnover organs with a cancer-inducing number of damage events of $c = 20$.

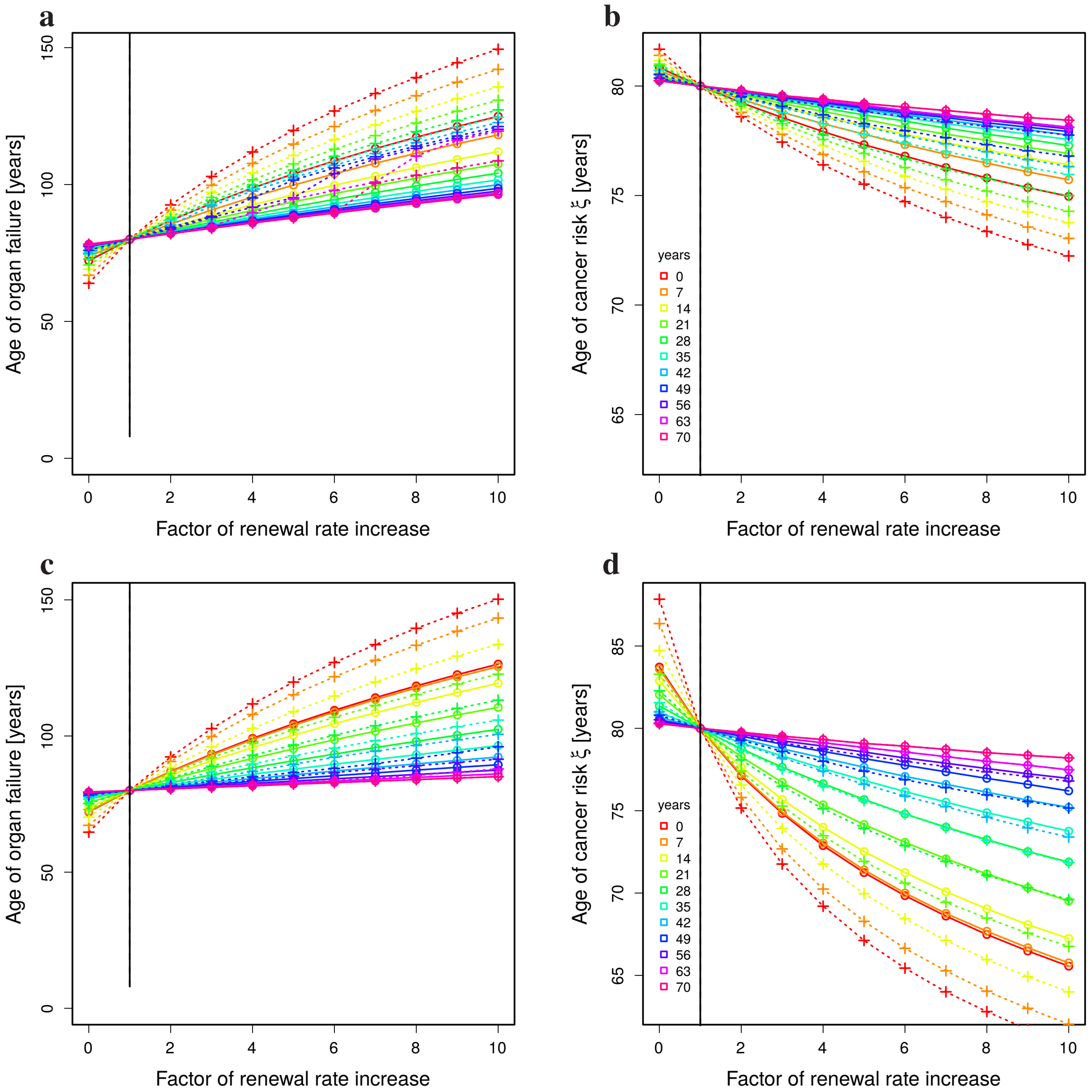

Supplementary Figure 42: Age- and division-dependent damage rate and damage rate kept age-specific during treatment Eqs. (16) and (17). Same analysis and representation as in Figure 35c,d with flat ($n_\mathrm{p} = 1.5$, **a,b**) and steep ($n_\mathrm{p} = 3$, **c,d**) age-dependence of the division rate in Eq. (15). Parameters of low turnover rates (see Table 1).

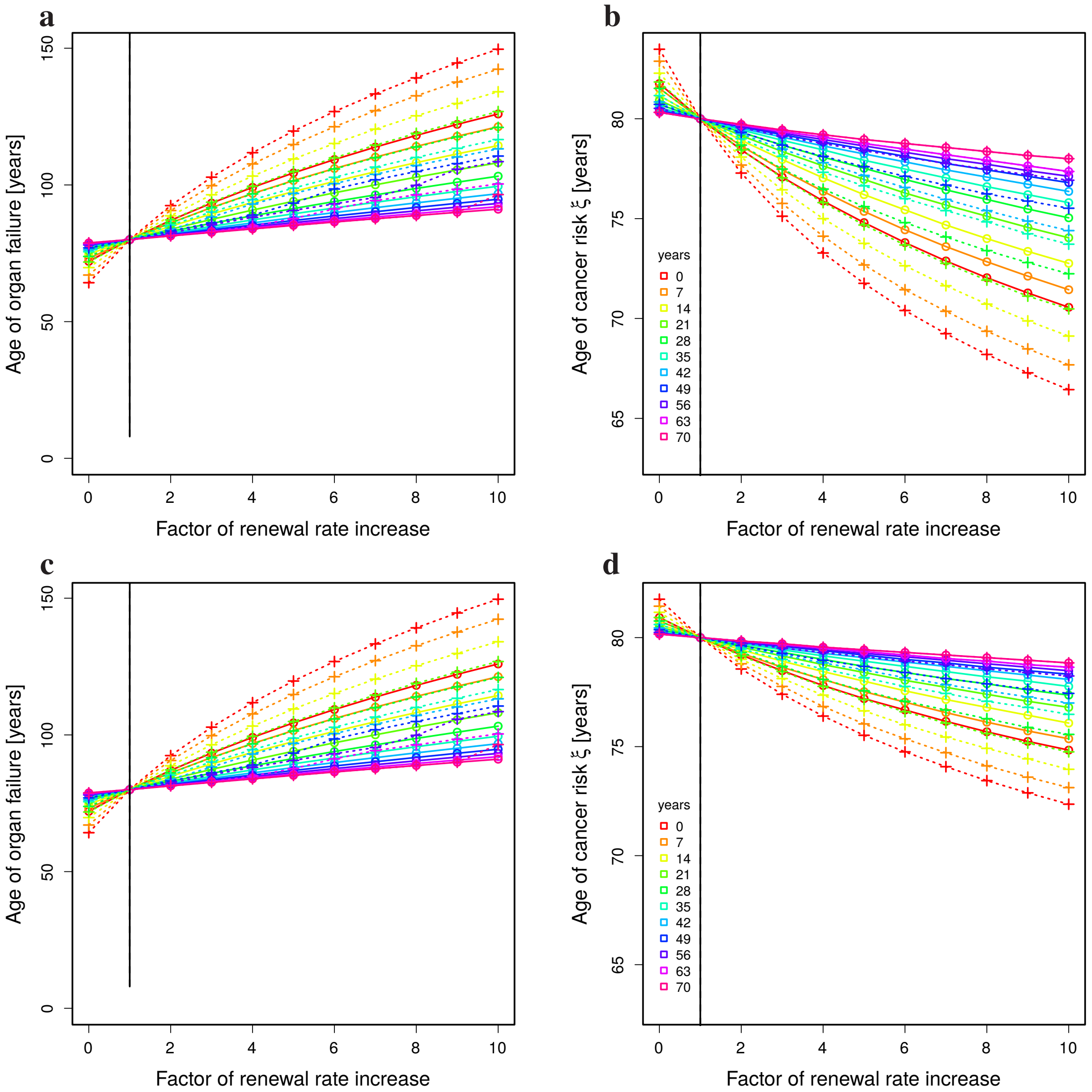

Supplementary Figure 43: Age- and division-dependent damage rate and damage rate kept age-specific during treatment Eqs. (16) and (17). Same analysis and representation as in Figure 35c,d with flat ($n_\gamma = 1.5$, **a,b**) and steep ($n_\gamma = 3$, **c,d**) age-dependence of the damage rate in Eq. (16). Parameters of low turnover rates (see Table 1).

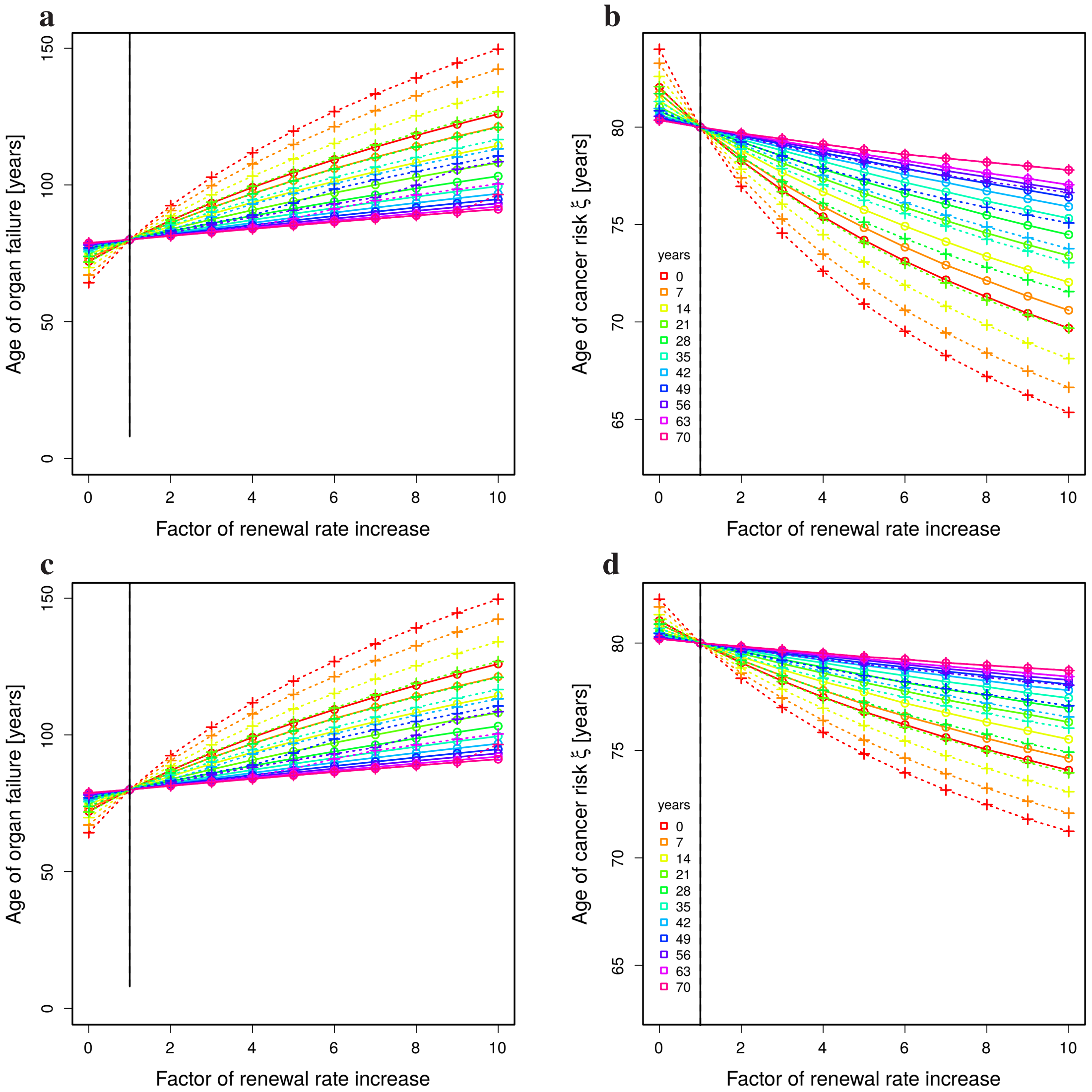

Supplementary Figure 44: Age- and division-dependent damage rate and damage rate kept age-specific during treatment Eqs. (16) and (17). Same analysis and representation as in Figure 35c,d with young ($K_\gamma = 30$, **a,b**) and old ($K_\gamma = 70$, **c,d**) age for half maximum damage rate in Eq. (16). Parameters of low turnover rates (see Table 1).
